# Association between Parkinson’s Disease and Cigarette Smoking, Rural Living, Well-Water Consumption, Farming and Pesticide Use: Systematic Review and Meta-Analysis

**DOI:** 10.1371/journal.pone.0151841

**Published:** 2016-04-07

**Authors:** Charles B. Breckenridge, Colin Berry, Ellen T. Chang, Robert L. Sielken, Jack S. Mandel

**Affiliations:** 1 Syngenta Crop Protection, LLC, Greensboro, NC, 27419, United States of America; 2 Queen Mary, Univ. of London, London, E1 4NS, United Kingdom; 3 Exponent, Inc., Menlo Park, CA, 94025, United States of America; 4 Sielken & Associates Consulting, Inc., College Station, TX, 77845, United States of America; Federal University of Viçosa, BRAZIL

## Abstract

**Objective:**

Bradford Hill’s viewpoints were used to conduct a weight-of-the-evidence assessment of the association between Parkinson’s disease (PD) and rural living, farming and pesticide use. The results were compared with an assessment based upon meta-analysis. For comparison, we also evaluated the association between PD and cigarette smoking as a “positive control” because a strong inverse association has been described consistently in the literature.

**Methods:**

PubMed was searched systematically to identify all published epidemiological studies that evaluated associations between Parkinson’s disease (PD) and cigarette smoking, rural living, well-water consumption, farming and the use of pesticides, herbicides, insecticides, fungicides or paraquat. Studies were categorized into two study quality groups (Tier 1 or Tier 2); data were abstracted and a forest plot of relative risks (RRs) was developed for each risk factor. In addition, when available, RRs were tabulated for more highly exposed individuals compared with the unexposed. Summary RRs for each risk factor were calculated by meta-analysis of Tier 1, Tier 2 and all studies combined, with sensitivity analyses stratified by other study characteristics. Indices of between-study heterogeneity and evidence of reporting bias were assessed. Bradford Hill’s viewpoints were used to determine if a causal relationship between PD and each risk factor was supported by the weight of the evidence.

**Findings:**

There was a consistent inverse (negative) association between current cigarette smoking and PD risk. In contrast, associations between PD and rural living, well-water consumption, farming and the use of pesticides, herbicides, insecticides, fungicides or paraquat were less consistent when assessed quantitatively or qualitatively.

**Conclusion:**

The weight of the evidence and meta-analysis support the conclusion that there is a causal relationship between PD risk and cigarette smoking, or some unknown factor correlated with cigarette smoking. There may be risk factors associated with rural living, farming, pesticide use or well-water consumption that are causally related to PD, but the studies to date have not identified such factors. To overcome the limitations of research in this area, future studies will have to better characterize the onset of PD and its relationship to rural living, farming and exposure to pesticides.

## Introduction

In 1817, James Parkinson identified “the shaking palsy,” later named Parkinson’s disease (PD), as a unique clinical entity in patients who presented four cardinal symptoms: tremor, bradykinesia, rigor and postural instability [1]. The disease is a major cause of morbidity in the elderly and its progression can impose a distressing burden of ill health. Death of dopaminergic (DA) neurons in the substantia nigra pars compacta (SNpc) and degeneration of its projections to the basal ganglia are the major, but not exclusive, findings in PD. Non-motor symptoms have been attributed to early changes in the olfactory tract and hindbrain, and late-stage changes have been described in subcortical and cortical structures [2]. Both the incidence and prevalence of PD increase with age, more so in men than in women [3].

Substantial advances have been made in understanding the role of gene mutations in familial PD [4, 5]. Studies on kindred subgroups have established that selected mutations of a number of parkin genes are linked to early- [6, 7] and late-onset [8] PD. Mutation-based impairment of chaperone proteins [9], as well as proteins involved with the ubiquitin proteasomal [10, 11] and the autophagy lysosomal [12, 13] systems, has been described. Mutation of α-synuclein [14], the protein aggregate present in Lewy bodies [15], has been characterized. In addition, proteins that play a role in mitochondrial function [16] and in the cellular response to oxidative stress [17] have been implicated in PD.

For idiopathic PD, where none of the established causal factors of parkinsonism have been identified, it is assumed that the combination of individual genetic susceptibility factors and environmental exposure to chemicals, pathogens or other factors may trigger or accelerate the onset of the disease. A major difficulty in investigating risk factors for PD is that some studies rely on self-reported PD [18–20], rather than including only patients whose PD diagnosis has been clinically confirmed. Litvan et al. emphasized that “this limitation strongly affects epidemiologic studies” [21]; also see Burn et al. [22]. Patients with multiple system atrophy (MSA), progressive supranuclear palsy, cortico-basal ganglionic degeneration, Lewy body disease or vascular disease may display parkinsonism. Even where diagnosis of PD is certain, there appear to be several PD subtypes that may have different etiologies [23].

Barbeau et al. [24, 25] were among the first researchers to report a positive correlation between PD prevalence in nine hydrographic basins in Quebec and the amount of pesticide sold within each basin. They discussed several limitations or “sources of error” in their study, including diagnostic accuracy, coding accuracy, accessibility to medical or specialist care, reporting completeness, migration of the patients, variation in prescription medicine doses, mean age of the populations and incomplete reporting of PD on death certificates. Many or all of these limitations also exist in epidemiologic studies of PD conducted by other researchers in subsequent years. Barbeau et al. [25] reported an approximate 20 percent difference in the number of prevalent PD cases ascertained between indirect methods (medical records of the province of Quebec or records of L-dopa sales) and direct diagnosis based on neurological examination. PD prevalence was greater in urban communities (cities with a population >50,000) than in the nine hydrographic basins, based on the state reporting system and L-dopa sales, but not when the cases were diagnosed by neurological examination.

Since Barbeau’s seminal work, numerous studies have evaluated the association between PD and rural living, well-water consumption, farming and pesticide use [26–30]. In the current study, we conducted a comprehensive assessment of the association between agriculture-related risk factors and PD because there is evidence in the literature to suggest that some component of the agricultural lifestyle contributes to the occurrence of PD. In order to contextualize the results from the analysis of agriculture-related factors, we also evaluated the association between PD and cigarette smoking because a consistent, strong inverse association has been reported in the published literature.

A systematic review of the published English epidemiological literature was done using standard methods for study identification, data retrieval and selection [31, 32]. In contrast to recent studies [28–30], this study used three complementary approaches to assess the potential causal relationship between PD, rural living and agricultural practices in any population. The narrative assessment approach utilized by Li et al. [26], Brown et al. [27], Wirdefeldt et al. [33], Friere and Koifman [34], and Moretto and Colosio [35] was augmented by a qualitative assessment of the weight of the evidence using the Bradford Hill viewpoints. The results from qualitative assessments were compared with those based on published meta-analyses [28–30, 36–38]. To judge the relative strength of associations with PD for a number of factors related to rural living and agricultural practices, the results were compared with those from studies assessing the relationship between PD and cigarette smoking, which consistently has shown an inverse association with PD [33, 37, 39, 40]. By using this analogy, we hoped to provide the reader with a reference point for judging whether the strength and consistency of the findings described in the present study are sufficient to conclude that causal relationships exist.

## Materials and Methods

A comprehensive search of the English-language literature was conducted to identify studies that evaluated associations between PD and a number of potential risk factors: cigarette smoking, rural living, well-water consumption, farming, any pesticide use and use of herbicides, fungicides, insecticides or paraquat (see S1 Appendix for search terms). In addition, bibliographies in recently published reviews and meta-analyses of pesticide use [26–29, 33] were evaluated for papers that fit the categories of interest.

Three searches were conducted in PubMed to identify articles describing smoking, rural (or associated agricultural) factors or paraquat in relation to PD; these searches identified a combined total of 1,145 potentially relevant articles, including 252 duplicates, leaving 893 unique articles. For each eligible study included in the analysis, the numbers of PD cases and comparison subjects with the potential risk factor were determined within the total number of subjects evaluated. All data were extracted and entered on a standardized form by one investigator, and independently confirmed by a second investigator. The estimated relative risk (RR), or odds ratio (OR) for case-control studies (hereafter both are referred to as RR), and the 95 percent CI were recorded, and forest plots were constructed for each risk factor. The herbicide paraquat was included in this analysis because it has been identified in some epidemiological studies as a potential risk factor for PD [41]. The organochlorine insecticide DDT and its metabolite dichlorodiphenyldichloroethylene (DDE) [33]; the dithiocarbamate fungicides maneb, ziram and mancozeb [42, 43]; and the natural insecticide rotenone [44], which have been reported as potential risk factors for PD, were not included because there were limited epidemiological data. To evaluate whether results were consistent with an exposure-response trend, RRs for heavier or longer-term cigarette smoking (e.g., more packs, years or pack-years smoked) and greater exposure to herbicides, fungicides, insecticides and paraquat (e.g., higher frequency or longer duration of use) were recorded separately.

When both unadjusted and adjusted RRs were reported in a study, the adjusted RR was selected. If adjusted RRs were not reported, crude RRs were included as reported or calculated from available raw data. If data for males and females were combined, this RR was selected. In some cases where stratified RRs were reported, weighted average RRs were calculated to combine stratum-specific RRs. When multiple RRs for related but distinct exposures were reported in a study, the RR judged to be closest to the exposure category of interest or to entail the highest level of exposure was selected. In some cases, more than one informative RR was selected from a given study (or from overlapping study populations described in multiple publications), but only non-overlapping RRs were included in the meta-analysis.

Evidence of bias in the reporting of associations with PD was evaluated by fitting a regression to the selected RRs using the unweighted least squares method described by Egger et al. [45]. We conducted a Monte Carlo simulation that showed that the unweighted least squares method was superior to weighted least squares analyses described by Egger et al. [45]. Funnel plots were used to appraise the asymmetry in the distribution of RRs. For each risk factor, an overall RR and 95% CI were calculated using the fixed effects meta-analysis procedure described by Egger et al. [31].

Publication bias was evaluated by testing the null hypothesis that publication bias does not exist, using the Egger et al. [45] regression test of unweighted ordinary least squares. According to Egger et al. [45], the null hypothesis is rejected if the p-value is less than 0.10. However, as a sensitivity analysis, we reported meta-analysis RRs and 95% CIs for Tier 1 studies (explained below), both with and without correction for publication bias for the fixed and random effects models, irrespective of whether Egger’s publication bias test statistic had a p-value of less than 0.10.

Publication bias was estimated and corrected for each Tier 1 (explained below) study dataset by using the trim-and-fill procedure employed by Pezzoli and Cereda [30], and described by Duval and Tweedie [46]. Accordingly, an iterative mathematical procedure was used, based upon a rank-based data augmentation technique, to estimate the number of missing studies and to fill in the missing RRs. These filled-in RRs might be either above or below the mean RR calculated from the original published studies. For example, for heavy/long-term smoking (p = 0.01), there were ten Tier 1 studies (fixed effects RR = 0.64; 95% CI = 0.58–0.71). After three iterations, using the L_0_^+^ estimator described by Duval and Tweedie [46], the procedure estimated that six studies were missing. The estimated RRs for these “missing studies” were all assigned values by the procedure that were greater that the RRs reported for the ten Tier 1 studies. In particular, the six filled-in RR values were as far above the mean as the six lowest published RRs were below the mean. In this example, the corrected meta-analysis RR and 95% CI were 0.70 and 0.64–0.76, respectively.

The percentage of the between-study variance in RRs attributable to study heterogeneity (I^2^) was calculated by replacing the Mantel-Haenszel measure of central tendency with the measure of central tendency based on inverse-variance, as described by Higgins et al. [47]. This method was used because it required only knowledge of the RRs and standard errors (SEs). The natural logarithm of the RR estimate [ln(RR)] was weighted proportionally to the reciprocal of its estimated variance, which was calculated as the square of the estimated SE. Statistically significant heterogeneity was indicated when the probability of obtaining the observed I^2^ was less than 0.05 (two-sided test).

To aid in the evaluation of heterogeneity in the observed associations, we classified all studies for each risk factor into two tiers according to their methodological rigor. Tier 1, indicating higher study quality, included studies with incident PD cases classified according to clinical data (e.g., medical records, physician confirmation and/or neurological examination) for diagnostic confirmation, as well as individual-level exposure assessment. Tier 2 included all other studies, i.e., those with prevalent PD cases, PD classified based on self-reporting or death certificates only, and/or ecologic (group-level) exposure assessment. We adopted this methodology because it was successfully used in a previous systematic review [48] based on the premise that causality cannot be inferred unless there is a clear determination that 1) individuals within studies were actually exposed to the risk factor; 2) diagnosis of PD in cases was confirmed by a PD specialist; 3) a suitable latency period existed between initial exposure to the risk factor and the occurrence of PD; and 4) the non-cases were confirmed to be disease-free. Furthermore, to reduce the potential for selection bias, we put more weight on studies that reported incident cases discovered during the study, rather than relying on cases that existed (prevalent cases) at the time of study commencement.

We conducted a sensitivity analysis to determine whether study characteristics, other than those used for the Tier 1/Tier 2 classification described above, affected meta-analysis results. We reviewed study attributes described in the Newcastle-Ottawa Scale [49] and the Research Triangle Institute Item Data Bank [50], and selected the following five additional attributes to categorize studies: 1) whether information on exposure was collected by personal interview or by some other means, such as a self-administered questionnaire; 2) for studies that assessed pesticide exposure, whether it was based on self-reported recall or was assigned by the investigator using a job-exposure matrix, a geographical model or some other method; 3) whether the study source population was based in a general population (i.e., population-based) or another setting; 4) for case-control studies, whether controls were population- or cohort-based (i.e., selected from an existing study cohort) or selected from another source (e.g., hospital-based or friend-based); and 5) whether the investigators adjusted for confounding by at least age, gender and cigarette smoking, or adjusted for fewer factors. These study characteristics were extracted from each publication and entered on a standardized form by one investigator, and were independently confirmed by a second investigator. We did not combine these attributes into a single scale, as others have done, because we could not be certain that any specific study feature would necessarily result in a “better-quality study.” Furthermore, we found that increasing the number of criteria used to categorize the studies resulted in few studies that fulfilled all the criteria and hence the subsequent meta-analysis was of limited utility.

Heterogeneity among studies was also judged by comparing the overall RR and 95% CI calculated using the fixed effects model to the RR and 95% CI calculated using the random effects model described by DerSimonian and Laird [51]. In a fixed effects model, all of the studies are assumed to be identical (i.e., with no heterogeneity) in the sense that they have an identical design aimed at estimating the magnitude of a fixed effect of treatment [52, 53]. In this situation, the weight assigned to an individual study for calculating the weighted average meta-analysis RR is proportional to the study’s precision (i.e., inversely proportional to its variance). The assumption that observational studies have identical design is rarely true, so as an alternative, a random effects model was also used. In the random effects model, because the individual studies may vary in design and conduct, it is assumed that each study represents a random sample from a distribution of all potential studies. In meta-analysis calculations based on the random effects model, the variance of ln(RR) is the sum of the variance (σ^2^) of the study ln(RR) around the study’s underlying mean ln(RR) (i.e., within-study variability) plus the variance (τ^2^) of that study mean around the underlying mean of the population of study means from which the particular study mean is randomly selected (i.e., between-study variability). Thus the weight assigned to a study in the random effects model is dependent on its precision and the between-study variance. The results of the fixed effects model were compared with those from the random effects model overall and within each tier of study quality or stratum of study characteristics in order to estimate the contribution of between-study heterogeneity to the calculated meta-analysis RR. The measure of between-study variability based on the random effects model (τ^2^) was also considered as an indicator of study heterogeneity. Overall, we adapted the guidance developed by Higgins and Green [53] on how to conduct meta-analysis for intervention studies to the present set of observational epidemiological studies.

In addition to these statistical analyses, a qualitative assessment of the weight of evidence for a causal relationship between each risk factor and PD was conducted. Bradford Hill’s “viewpoints”, as summarized in Table 1, were used to assess whether the studies supported a causal exposure-disease association. These viewpoints include the strength of the association, the consistency of results among studies, evidence of a biological gradient, evidence that “exposure” preceded the occurrence of disease with an appropriate latency period, the specificity of the risk factor being evaluated, the coherence of the relationship, the biological plausibility that the risk factor could cause PD and whether analogies exist to other exposure-disease associations that are interpreted to be causal. There were no experimental studies among those identified (Table 1). In this assessment, higher-quality studies, as defined previously, were given more weight than were studies of lower quality.

**Table 1:** Bradford Hill viewpoints.

**Strength**	The larger the relative risk (RR), the more likely the association is to be causal.
**Consistency**	Consistent associations between exposure and disease observed in independent studies indicate that the observed associations are more likely to be causal.
**Specificity**	An association that is specific for a particular group of individuals or for a disease is more likely to be causal.
**Temporality**	For a factor to be causal, it must precede the disease. This is the only condition that is considered essential.
**Biological Gradient**	If the risk of disease increases with increasing dose (exposure), then the association is more likely to be causal.
**Plausibility**	If it is plausible that the agent could cause the disease, based upon current knowledge, then the association is more likely to be causal.
**Coherence**	If the findings are consistent with other data, for example, the known distribution of the disease in the population, then the association is more likely to be causal.
**Experiment**	A causal inference is more likely if it is supported by experimentation. For example, if the implementation of a preventive measure such as reducing exposure results in reduced risk, then the association between exposure and disease is more likely to be causal.
**Analogy**	Analogy of the current association with another exposure-disease relationship can be used to support an inference of causality.

## Results

### Study Identification and Eligibility

Three searches were conducted in PubMed to identify articles on smoking, rural living (or associated agricultural factors) or paraquat in relation to PD; these searches identified a combined total of 1,145 potentially relevant articles, including 252 duplicates, leaving 893 unique articles (S2 Appendix). An additional 30 potentially relevant articles were identified from published bibliographies. Based on a review of the titles and abstracts of all 923 articles, we excluded 677 articles without pertinent information, leaving 246 articles for full-text review. After reviewing these articles, we further excluded 133 that were not relevant or did not contain sufficient data for meta-analysis (i.e., RRs and 95% CIs, or raw frequencies), leaving 113 articles eligible for meta-analysis. Eight of these reported sets of results completely overlapped with results from another eligible article and were ultimately excluded. Overall, 105 articles were included in meta-analyses of any of the 14 exposures considered. Many of these articles contained data relating to multiple risk factors.

### Study Classification

Key information used to categorize each study as either a Tier 1 or Tier 2 study is summarized in Tables A-I in S3 File. Overall, approximately 20 percent of the studies were in Tier 1 (Table 2). Thirty-three percent of studies on current cigarette smoking (11 studies) were Tier 1 studies, whereas rural living, well-water consumption and farming had 14 percent (four studies), 14 percent (five studies) and 17 percent (eight studies) Tier 1 studies, respectively. The percentage of studies on pesticides that were Tier 1 ranged from eight percent for studies on paraquat (one study) to 30 percent for studies on fungicides (three studies). There were 11 Tier 1 studies (22%) that reported any pesticide use. Among studies that evaluated high pesticide use, there were either no Tier 1 studies (paraquat) or only one Tier 1 study (herbicide, fungicide or insecticide use).

**Table 2:** Number of studies assigned to each category (Tier 1, Tier 2, exposure interview technique, pesticide exposure assessment, source population, type of controls and adjustment for confounders) for each environmental or lifestyle risk factor.

Risk Factor*	All Studies	Study Tier		Exposure Interview		Pesticide Exposure†		Source Population		Controls‡		Confounder Adjustment	
		Tier 1	Tier 2	In-Person	Other	Self-Reported	Assigned	Pop.- Based	Other	Cohort/Pop.-Based	Other	A,G,S^#^	Other
**Current Cigarette Smoking**	33	11	22	15	18	—	—	8	25	8	17	27	6
**Heavy/Long-Term Cigarette Smoking**	37	10	27	14	23	—	—	7	30	9	21	33	4
**Rural Living**	29	4	25	18	11	—	—	1	28	1	28	7	22
**Well-Water Consumption**	35	5	30	23	12	—	—	2	33	4	31	12	23
**Farming**	48	8	40	25	23	—	—	16	32	14	26	19	29
**Pesticide Use**	49	11	38	25	24	40	9	15	34	14	29	19	30
**Herbicide Use**	18	4	14	9	9	17	1	4	14	6	12	10	8
**Fungicide Use**	10	3	7	5	5	9	1	3	7	4	6	6	4
**Insecticide Use**	17	4	13	9	8	16	1	5	12	6	11	8	9
**Paraquat Use**	13	1	12	6	7	10	3	2	11	5	6	8	5
**High Herbicide Use**	10	1	9	4	6	10	0	2	8	4	4	4	6
**High Fungicide Use**	5	1	4	0	5	5	0	1	4	2	3	3	2
**High Insecticide Use**	8	1	7	2	6	8	0	2	6	3	5	4	4
**High Paraquat Use**	4	0	4	1	3	3	1	0	4	2	2	3	1
**Total Number of RRs Evaluated**	316	64	252	156	160	118	16	68	248	82	201	163	153
**Percent of Total**		20	80	49	51	88	12	22	78	29	71	52	48

*Two independent relative risks (RRs) from [54] were counted for rural living; two independent RRs each from [55] were counted for high herbicide use, high fungicide use and high insecticide use; and two independent RRs each from [56] and [57] were counted for pesticide use.

†Classified only with respect to pesticide exposure assessment.

‡Classified only for case-control studies.

# A = age; G = gender; S = smoking; studies of current and heavy/long-term cigarette smoking were classified in this category if they adjusted for age and gender.

Most of the studies (88%) of pesticide exposure relied on self-reported pesticide use obtained either through personal interviews (49%) or by some other method. Twenty-two percent of all included studies were population-based and 29 percent of case-control studies used population- or cohort-based controls. Fifty-two percent of all studies adjusted the RRs for age, gender and cigarette smoking, with fewer adjustments being made in the other 48 percent of the studies.

### Heterogeneity of Study Results

The heterogeneity of results for Tier 1 and Tier 2 studies combined, as measured by I^2^, was statistically significant for all risk factors examined except fungicides (Table 3). Using the random effects model, the between-study variances (τ^2^) of the individual study RRs for Tier 1, Tier 2 and Tier 1 & Tier 2 studies combined were calculated (Table 4, Figs A-J in S1 File). The results indicate that τ^2^ tended to be larger (i.e., the study RRs were more heterogeneous) when all studies were combined than when τ^2^ was calculated for Tier 1 studies. However, τ^2^ was greater in Tier 1 studies on current cigarette smoking and rural living than it was in either Tier 2 studies or across all studies combined.

**Table 3:** Fixed and random effects meta-analysis relative risk (RR) estimates and their 95% confidence intervals (CIs) for the association between Parkinson’s disease and each risk factor based on Tier 1 studies and all studies combined.

Risk Factor	All Studies RR (95% CI)		Tier 1 Studies RR (95% CI)		Heterogeneity^a^ I^2^, N, p-value		Publication Bias^b^ (p value)	Meta-Analysis RR (95% CI) after Trim-&-Fill Adjustment for Publication Bias^c^ Tier 1 Studies	
	Fixed Effects	Random Effects	Fixed Effects	Random Effects	All Studies	Tier 1 Studies	Tier 1 Studies	Fixed Effects	Random Effects
Current Smoking vs. Never Smoking (Fig 1)	0.46*(0.42, 0.51)	0.41* (0.34, 0.48)	0.41* (0.35, 0.48)	0.35* (0.25, 0.49)	65.2% (N = 33) p < 0.0001	73.6% (N = 11) p < 0.0001	p = 0.16	0.54* (0.47, 0.62)	0.55* (0.39, 0.78)
Heavy/Long-Term Smoking vs. Never Smoking (Fig 2)	0.60* (0.56, 0.65)	0.49* (0.43, 0.57)	0.64* (0.58, 0.71)	0.55* (0.45, 0.67)	63.8% (N = 37) p < 0.0001	70.4% (N = 10) p = 0.0004	p = 0.01^b^	0.70* (0.64, 0.76)	0.69* (0.57, 0.83)
Rural Living vs. Non-Rural Living (Fig 3)	1.17* (1.10, 1.24)	1.43* (1.22, 1.69)	1.28* (1.02, 1.62)	1.52 (0.85, 2.71)	75.5% (N = 29) p < 0.0001	80.2% (N = 4) p = 0.0017	p = 0.34	1.28* (1.02, 1.62)	1.52 (0.85, 2.71)
Well-Water vs. Non-Well-Water Consumption (Fig 4)	1.02 (0.98, 1.07)	1.30* (1.12, 1.51)	1.50* (1.20, 1.87)	1.48* (1.02, 2.15)	78.2% (N = 35) p < 0.0001	61.1% (N = 5) p = 0.0360	p = 0.82	1.50* (1.20, 1.87)	1.48* (1.02, 2.15)
Farming vs. Non-Farming (Fig 5)	1.08* (1.04, 1.11)	1.24* (1.12, 1.37)	1.28* (1.05, 1.57)	1.28 (0.99, 1.66)	73.9% (N = 48) p < 0.0001	33.1% (N = 8) p = 0.1637	p = 0.33	1.28* (1.05, 1.57)	1.28 (0.99, 1.66)
Pesticide Use vs. Non-Use (Fig 6)	1.22* (1.18, 1.27)	1.56* (1.37, 1.77)	1.32* (1.16, 1.52)	1.40* (1.06, 1.85)	77.4% (N = 49) p < 0.0001	64.6% (N = 11) p = 0.0016	p = 0.41	1.14* (1.01, 1.29)	1.11 (0.82, 1.50)
Herbicide Use vs. Non-Use (Fig 7A)	1.20* (1.06, 1.36)	1.20* (1.00, 1.43)	1.30* (1.01, 1.68)	1.30* (1.01, 1.68)	41.2% (N = 18) p = 0.0352	0.0% (N = 4) p = 0.8647	p = 0.42	1.26 (0.99, 1.60)	1.26 (0.99, 1.60)
High Herbicide Use vs. Non-Use (Fig 8A)	1.49* (1.08, 2.06)	1.65* (1.02, 2.66)	—	—	49.8% (N = 10)p = 0.0360	N = 1	—	—	—
Fungicide Use vs. Non-Use (Fig 7B)	0.94 (0.75, 1.19)	0.96 (0.74, 1.25)	0.85 (0.48, 1.49)	0.85 (0.48, 1.49)	12.8% (N = 10) p = 0.3256	0.0% (N = 3) p = 0.3702	p = 0.96	0.85 (0.48, 1.49)	0.85 (0.48, 1.49)
High Fungicide Use vs. Non-Use (Fig 8B)	1.39 (0.88, 2.19)	1.53 (0.74, 3.16)	—	—	53.9% (N = 5) p = 0.0698	N = 1	—	—	—
Insecticide Use vs. Non-Use (Fig 7C)	1.32* (1.14, 1.52)	1.46* (1.01, 2.11)	1.04 (0.83, 1.31)	1.36 (0.76, 2.45)	81.0% (17) p < 0.0001	64.3% (N = 4) p = 0.0385	p = 0.22	1.04 (0.83, 1.31)	1.36 (0.76, 2.45)
High Insecticide Use vs. Non-Use (Fig 8C)	2.18* (1.55, 3.06)	2.59* (1.49, 4.49)	—	—	55.1% (N = 8) p = 0.0293	N = 1	—	—	—
Paraquat Use vs. Non-Use (Fig 9A)	1.69* (1.44, 1.98)	1.47* (1.01, 2.13)	—	—	69.9% (N = 13) p = 0.0001	N = 1	—	—	—
High Paraquat Use vs. Non-Use (Fig 9B)	1.75* (1.19, 2.57)	1.99 (0.84, 4.71)	—	—	78.9% (N = 4) p < 0.0026	N = 0	——	—	—

^a^ The heterogeneity test statistic is analogous to the Mantel-Haenszel I^2^, except the measure of central tendency is based on an inverse-variance method.

^b^ The null hypothesis of no reporting bias is rejected at the 10% significance level. Egger et al. [45] regression test, unweighted ordinary least squares.

^c^ The trim-and-fill procedure is described by Duval and Tweedie [46].

* RR is statistically significantly different from 1.0 at the 5% significance level.

**Table 4:** Heterogeneity estimates for each risk factor based on between-study variance (τ^2^) among Tier 1, Tier 2 or Tiers 1&2 studies combined.

Risk Factor	(A) Estimated Variance in Population from Which Tiers 1&2 Studies Combined Were Drawn	(B) Estimated Variance in Population from Which Tier 1 Studies Were Drawn	(C) Estimated Variance in Population From Which Tier 2 Studies Were Drawn	(D) Relative Heterogeneity in Tier 1 Studies vs. Tiers 1&2 Studies Combined: 100%×(B)/(A)	(E) Relative Heterogeneity in Tier 1 Studies vs. Tier 2 Studies:100%×(B)/(C)
Current Smoking vs. Never Smoking–Fig 1	0.15	0.19	0.14	126%	139%
Heavy/Long-Term Smoking vs. Never Smoking–Fig 2	0.09	0.05	0.15	56%	35%
Rural Living vs. Non-Rural Living–Fig 3	0.12	0.27	0.12	225%	245%
Well-Water vs. Non-Well-Water Consumption–Fig 4	0.12	0.11	0.12	87%	90%
Farming vs. Non-Farming–Fig 5	0.06	0.04	0.06	78%	77%
Pesticide Use vs. Never Use–Fig 6	0.11	0.11	0.12	104%	94%
Herbicide Use vs. Non-Use–Fig 7A	0.06	0	0.09	0%	0%
Fungicide Use vs. Non-Use–Fig 7B	0.02	0	0.05	0%	0%
Insecticide Use vs. Non-Use–Fig 7C	0.42	0.21	0.57	50%	37%
Paraquat Use vs. Non-Use–Fig 9A	0.23	NA^1^	0.24	NA	NA

^1^ NA: not available; variance could not be estimated because there was only one Tier 1 study in this category.

Between-study variance of RRs was lowest for fungicide use, independent of study classification, and greatest for insecticide use, rural living and paraquat use (Table 4). For insecticide use, the Tier 2 study by Das et al. [58] was an “outlier” study that contributed the most to the Tier 2 between-study heterogeneity; removal of this study resulted in a 49 percent reduction in τ^2^ from 0.57 to 0.29. As another example, the Tier 2 study by Liou et al. [59] contributed the most to the Tier 2 between-study variability for paraquat; when this study was removed, τ^2^ was reduced almost 10-fold, from 0.24 to 0.03.

### Publication Bias

A statistically significant regression test statistic for publication bias in Tier 1 studies (p = 0.01) was observed for heavy/long-term smoking, but not for any other potential risk factor (Table 3). Correction for publication bias for heavy/long-term smoking did not substantially change the magnitude of the RR or the range of the 95% CI (Table 3, columns 4 and 5 vs. columns 9 and 10). For other possible risk factors, the meta-analysis RR based on Tier 1 studies was no longer statistically significant after publication bias correction for pesticide use (random effects model) and for herbicide use (random and fixed effects models); however, all changes were small in magnitude and largely inconsequential.

### Cigarette Smoking

**Fig 1:**
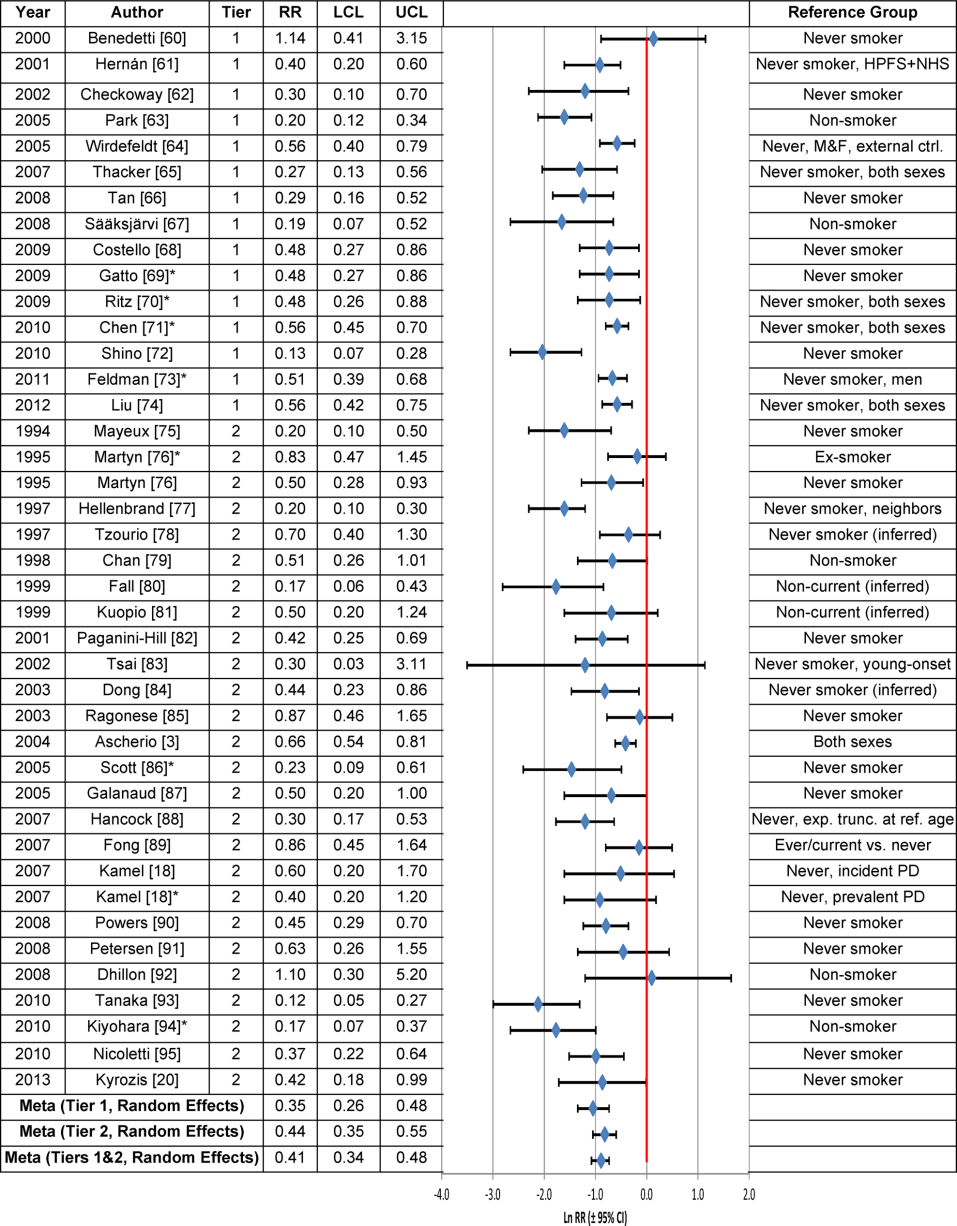
Association between current cigarette smoking and Parkinson’s disease. The natural logarithm of the estimated relative risk [ln(RR)] and the 95% confidence interval for each study are displayed. Current smokers have a significantly greater or lower risk of PD than non-smokers if the horizontal line for the study is to the right or to the left, respectively, of the bold vertical line [ln(RR) = 0] and does not cross it. PD risk for current smokers is similar to that in non-smokers if the horizontal line for the study crosses the bold vertical axis. An asterisk (*) denotes RR estimates that are not included in the meta-analysis due to study overlap with another RR estimate shown in the figure. RR = relative risk, LCL = lower limit of the 95% confidence interval, UCL = upper limit of the 95% confidence interval, HPFS = Health Professionals Follow-Up Study, M & F = males and females, NHS = Nurses’ Health Study, PD = Parkinson’s disease. Citations for studies appearing in this figure can be found here: [3, 18, 20, 60–95].

**Fig 2:**
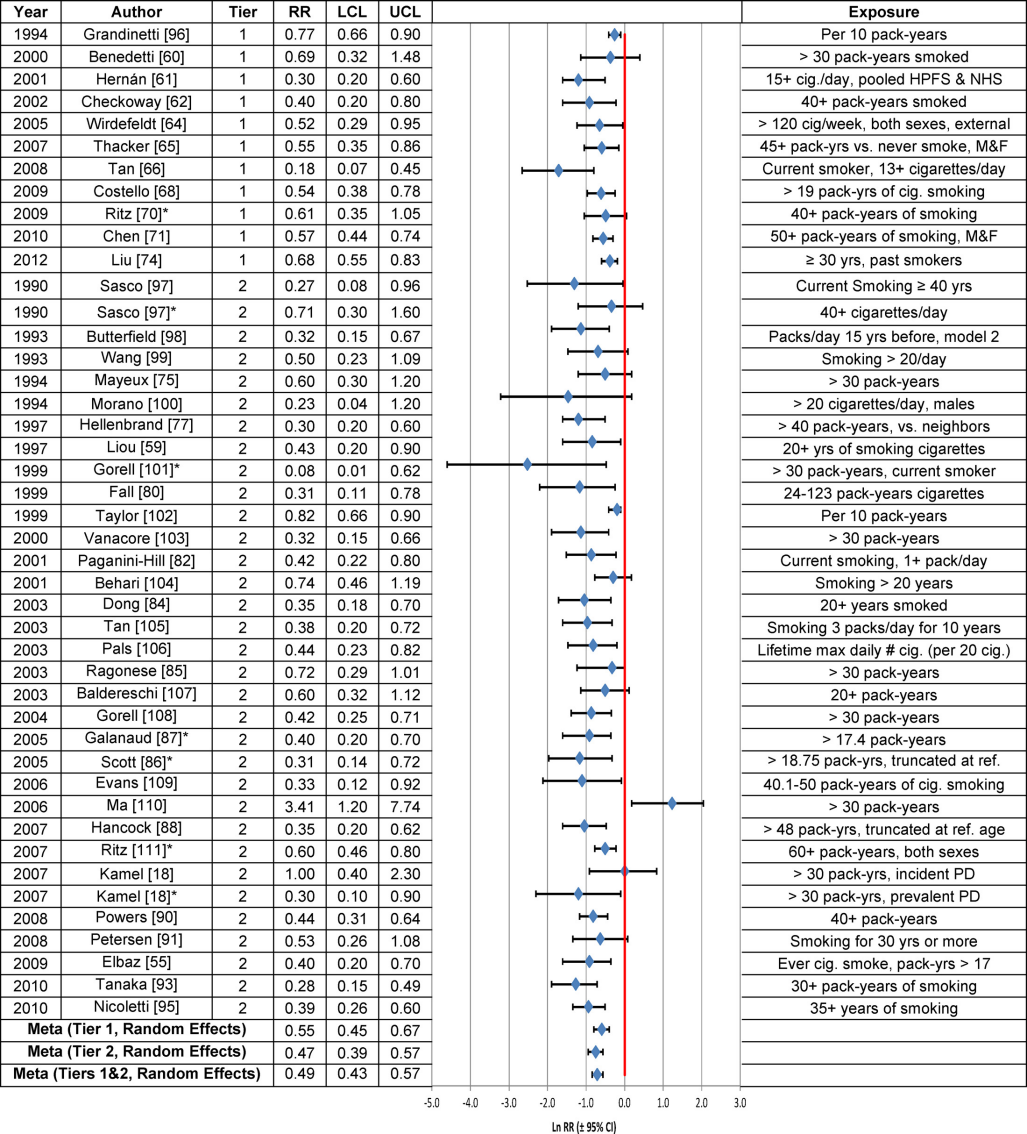
Association between the heavy or long-term cigarette smoking and Parkinson’s disease. The natural logarithm of the estimated relative risk [ln(RR)] and the 95% confidence interval for each study are displayed (see the legend for Fig 1 for instructions on how to interpret forest plots). An asterisk (*) denotes RR estimates that are not included in the meta-analysis due to study overlap with another RR estimate shown in the figure. RR = relative risk, LCL = lower limit of the 95% confidence interval, UCL = upper limit of the 95% confidence interval, M & F = males and females, PD = Parkinson’s disease. Citations for studies appearing in this figure can be found here: [18, 55, 59–62, 64–66, 68, 70, 71, 74, 75, 77, 80, 82, 84–88, 90, 91, 93, 95–111].

In the majority of studies, the risk of PD was statistically significantly reduced in current cigarette smokers compared with non-smokers (Fig 1). Sixty-seven percent of the 33 epidemiological studies evaluated and 10 out of 11 (the exception is Benedetti et al. [60]) Tier 1 studies reported statistically significantly decreased PD risk in cigarette smokers (Table A in S4 File and Table A in S5 File). In studies where the cigarette smoking “dose” was estimated based on packs, years and/or pack-years of smoking, PD risk was statistically significantly reduced in heavy or long-term smokers compared with non-smokers in 73 percent of the studies (90% of Tier 1 studies) (Fig 2, Table B in S4 File and Table B in S5 File). Overall, current cigarette smokers had a statistically significantly reduced risk of PD compared with non-smokers, irrespective of whether risk was assessed using a fixed effects (RR = 0.46; 95% CI = 0.42–0.51) or a random effects model (RR = 0.41; 95% CI = 0.34–0.48) (Table 3). Among Tier 1 studies, the fixed effects RR was 0.41 (95% CI = 0.35–0.48) and the random effects RR was 0.35 (95% CI = 0.25–0.49). Results were similar in studies that separately classified heavy or long-term smokers. The overall (Tiers 1 and 2 combined) estimate of the association between heavy/long-term cigarette smoking and PD was robust and insensitive to stratification by other study characteristics. However, the overall RR estimates tended to be slightly lower when calculated using the random effects model than when using the fixed effects model (Fig A in S2 File).

The regression test statistic for publication bias was not statistically significant in Tier 1 studies of current smokers vs. never smokers (Table 3). However, there was statistically significant publication bias in the assessment of the association between heavy/long-term smoking and PD. The RR adjusted for publication bias using the trim-and-fill procedure was still statistically significantly negative, irrespective of whether a fixed (RR = 0.70; 95% CI = 0.64–0.76) or random effects model (RR = 0.69; 95% CI = 0.57–0.83) was used (Table 3).

An assessment of PD risk in cigarette smokers based on Bradford Hill’s viewpoints (Table 5) indicates a consistent, approximately two-fold reduction in PD risk. We did not formally assess biological gradient (dose-response) in these studies, although when PD risk was evaluated in heavy or long-term smokers (Fig 2), it was reduced, but not to a greater extent than was observed in current smokers (Table 3). Despite compelling statistical evidence of an inverse relationship between the risk of developing PD and cigarette smoking, the underlying biological basis for this relationship is unknown.

**Table 5:** Weight-of-the-evidence assessment of causality based upon Bradford Hill viewpoints.

Bradford Hill Viewpoint	Cigarette Smoking	Rural Living	Well-Water Consumption	Farming	Any Pesticide Use	Herbicide Use	Fungicide Use	Insecticide Use	Paraquat Use
**Strength of Association (RR) in Tier 1 Studies**	0.54*	1.28*	1.50*	1.28*	1.14*	1.26†	0.85†	1.36†	0.90‡
**Biological Gradient**	Pack-Years Assessed	Not Evaluated	Not Evaluated	Not Evaluated	Not Evaluated	No High-Use Tier 1 Studies	Limited High-Use Tier 1 Studies	Limited High-Use Tier 1 Studies	No High-Use Tier 1 Studies
**Temporality**	Not Established	Not Established	Not Established	Not Established	Not Established	Not Established	Not Established	Not Established	Not Established
**Consistency**	Consistent	Inconsistent	Inconsistent	Inconsistent	Consistent	Inconsistent	Consistently Null	Inconsistent	Inconsistent
**Specificity**	Not Specific	Not Specific	Not Specific	Not Specific	Not Specific	Not Specific	Not Specific	Not Specific	Highly Specific
**Plausibility**	Uncertain§	No	No	No	No	No	No	No	Uncertainǁ
**Coherence**	Moderate	No	No	No	No	No	No	No	No
**Experimental Evidence**	No Studies	No Studies	No Studies	No Studies	No Studies	No Studies	No Studies	No Studies	No Studies
**Analogy**	Analogies Exist	No Analogies	No Analogies	No Analogies	No Analogies	No Analogies	No Analogies	No Analogies	Analogies Exist

* Stronger statistically significant RR (from fixed or random effects model) after correction for reporting bias.

† Stronger statistically non-significant RR (from fixed or random effects model) after correction for reporting bias.

‡ Statistically non-significant RR from one study.

§ Although the epidemiological data show a consistent, approximately two-fold reduction in PD risk in individuals who smoke cigarettes, no constituent of cigarette smoke has been identified as being neuroprotective [112] and a mechanism of action has not been elucidated.

ǁ Because paraquat is capable of redox recycling, it is plausible that paraquat could damage dopaminergic neurons in the substantia nigra. However, controversy exists in the published literature as to whether there are effects of paraquat in animal models or whether paraquat, under conditions of human exposure, reaches critical regions of the brain at concentrations sufficient to trigger adverse effects [113].

### Rural Living, Farming and Well-Water Consumption

Rural living, farming and well-water consumption were assessed together because of potential inter-dependence of findings related to these three aspects of a rural or agricultural lifestyle. Within studies that evaluated at least two of the three exposures, there was a positive, but not statistically significant, correlation between RRs for rural living and well-water consumption (r = 0.27; p = 0.24; 21 studies). There was no correlation between RRs for rural living and farming (r = - 0.02; p = 0.94; 14 studies) or between RRs for well-water consumption and farming (r = - 0.02; p = 0.91; 23 studies).

**Fig 3:**
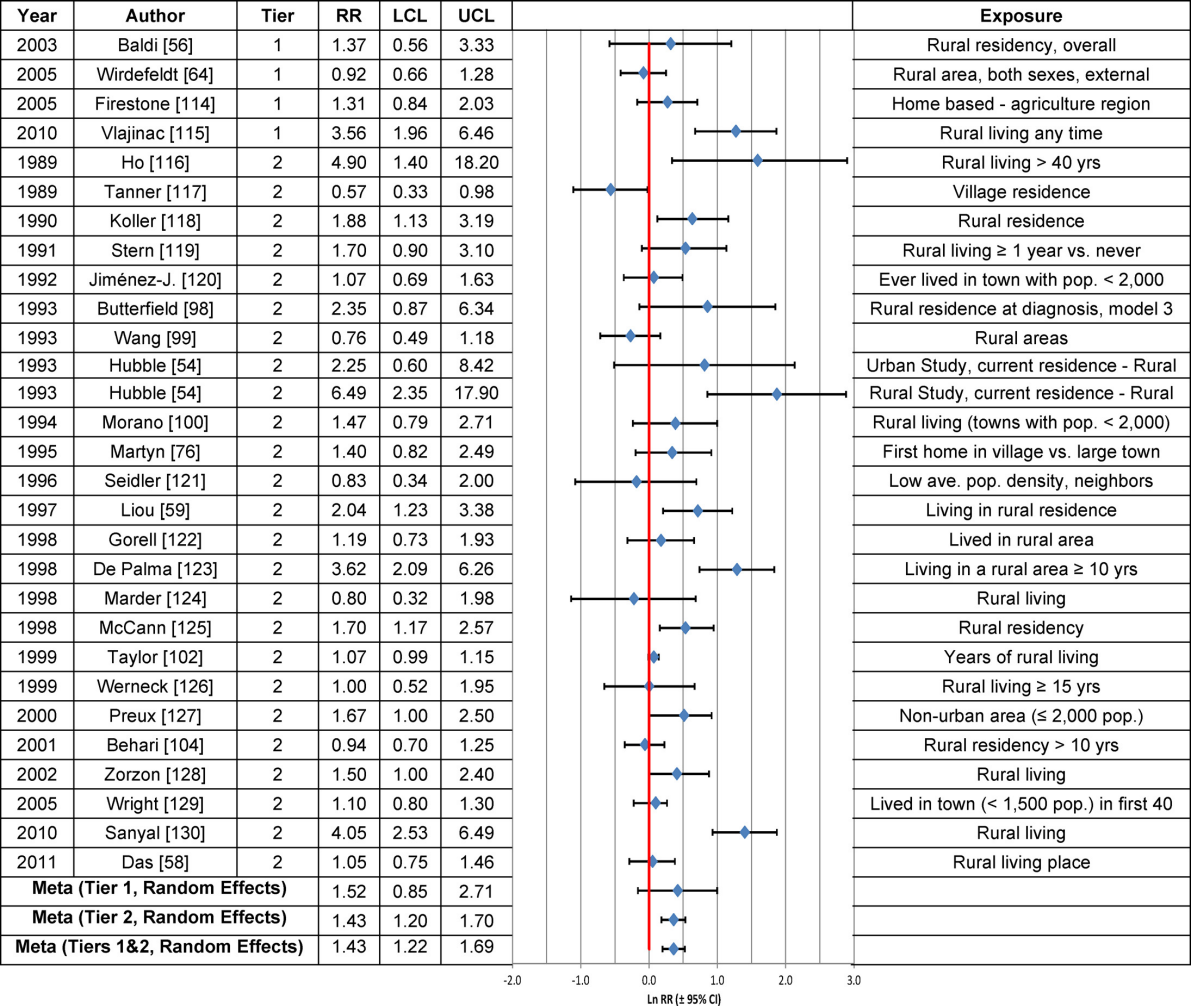
Association between rural living and Parkinson’s disease. The natural logarithm of the estimated relative risk [ln(RR)] and the 95% confidence interval for each study are displayed (see the legend for Fig 1 for instructions on how to interpret forest plots). RR = relative risk, LCL = lower limit of the 95% confidence interval, UCL = upper limit of the 95% confidence interval. Citations for studies appearing in this figure can be found here: [54, 56, 58, 59, 64, 76, 98–100, 102, 104, 114–130].

**Fig 4:**
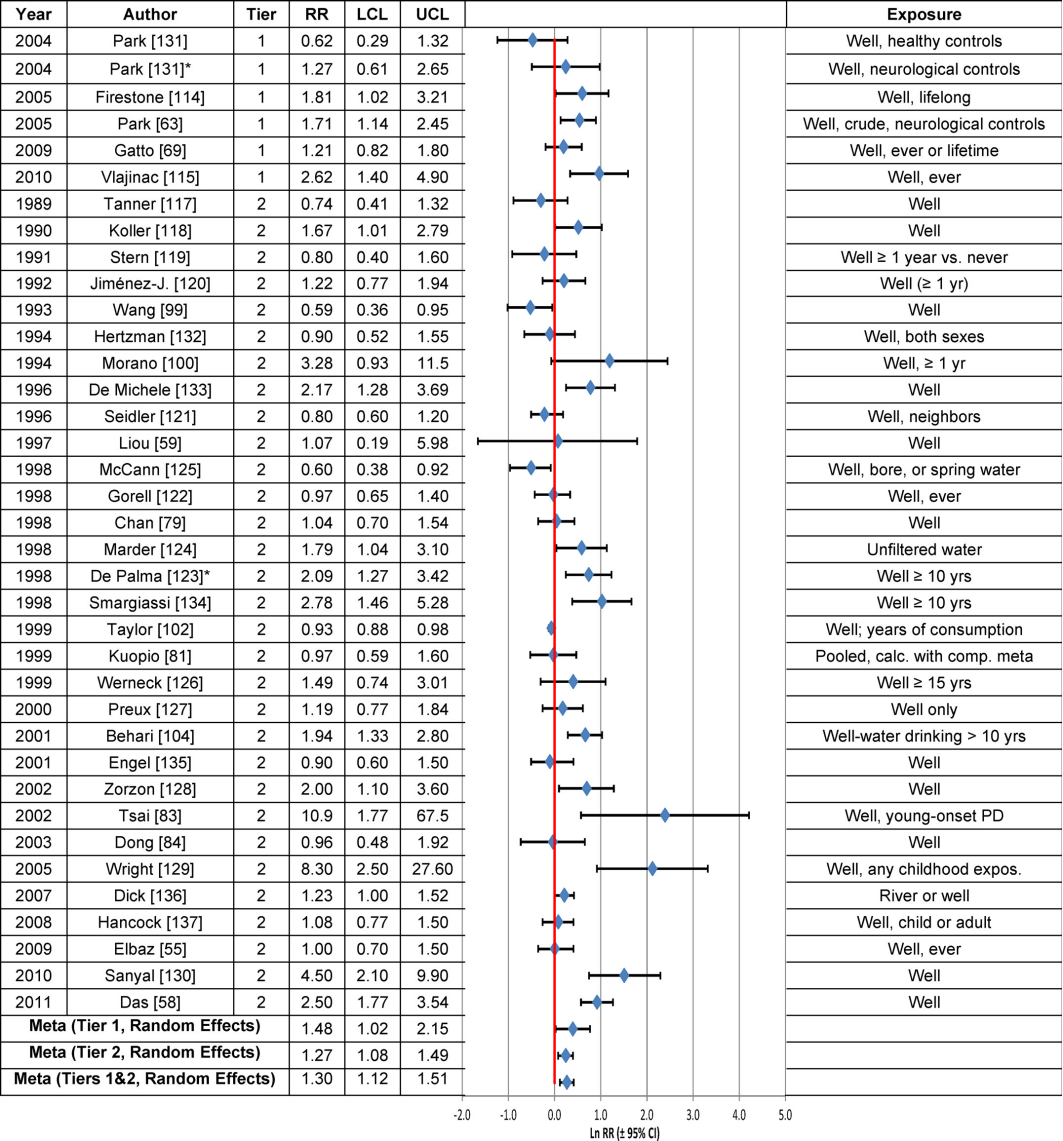
Association between well-water consumption and Parkinson’s disease. The natural logarithm of the estimated relative risk [ln(RR)] and the 95% confidence interval for each study are displayed (see the legend for Fig 1 for instructions on how to interpret forest plots). An asterisk (*) denotes RR estimates that are not included in the meta-analysis due to study overlap with another RR estimate shown in the figure. RR = relative risk, LCL = lower limit of the 95% confidence interval, UCL = upper limit of the 95% confidence interval, PD = Parkinson’s disease. Citations for studies appearing in this figure can be found here: [55, 58, 59, 63, 69, 79, 81, 83, 84, 99, 100, 102, 104, 114, 115, 117–137].

**Fig 5:**
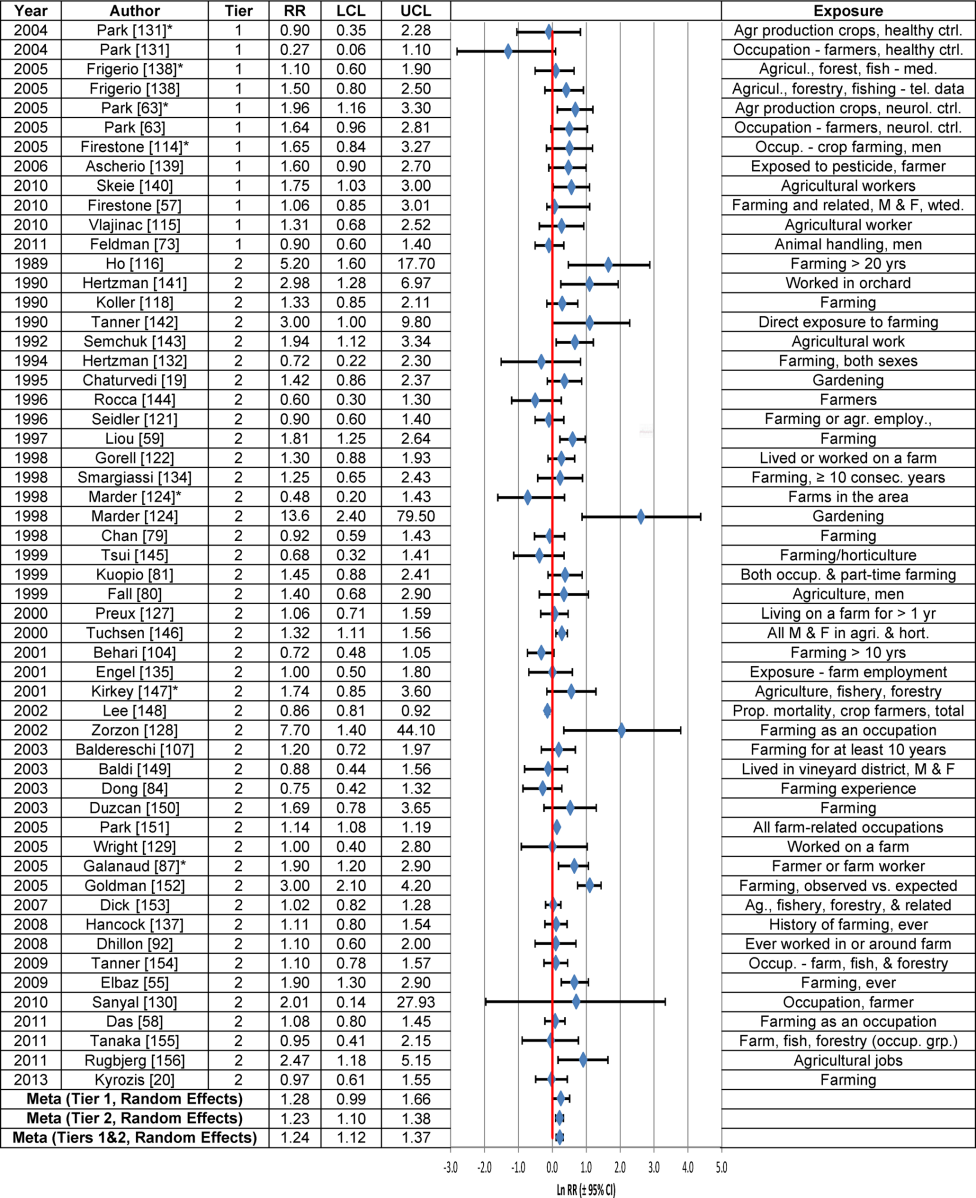
Association between farming and Parkinson’s disease. The natural logarithm of the estimated relative risk [ln(RR)] and the 95% confidence interval for each study are displayed (see the legend for Fig 1 for instructions on how to interpret forest plots). An asterisk (*) denotes RR estimates that are not included in the meta-analysis due to study overlap with another RR estimate shown in the figure. RR = relative risk, LCL = lower limit of the 95% confidence interval, UCL = upper limit of the 95% confidence interval, M & F = males and females. Citations for studies appearing in this figure can be found here: [19, 20, 55, 57–59, 63, 73, 79–81, 84, 87, 92, 104, 107, 114–116, 118, 121, 122, 124, 127–132, 134, 135, 137–156].

Forest plots of the RRs from studies that evaluated the association between PD risk and rural living, well-water consumption or farming are provided in Figs 3, 4 and 5, respectively, and the meta-analysis results are summarized in Table 3. Statistically significant associations were observed between PD risk and rural living (RR = 1.17; 95% CI = 1.10–1.24) and farming (RR = 1.08; 95% CI = 1.04–1.11), but not well-water consumption (RR = 1.02; 95% CI = 0.98–1.07), based on the fixed effects model. The overall meta-analysis RR, based on the random effects model, was statistically significant for all three variables: rural living (RR = 1.43; 95% CI = 1.22–1.69), farming (RR = 1.24; 95% CI = 1.12–1.37) and well-water consumption (RR = 1.30; 95% CI = 1.12–1.51). Similar results were obtained for Tier 1 studies, both before and after correction for publication bias.

Meta-analysis RRs calculated based on Tier 2 studies (Tables C-E in S4 File) were lower than those based on Tier 1 studies (Table 3), largely due to the fact that among the Tier 2 studies for each type of exposure, there was at least one study with a near null result that was weighted heavily, based on a narrow 95% CI. Thus, for rural living, the RR reported in a Tier 2 study by Taylor et al. [102] (RR = 1.07; 95% CI = 0.99–1.15) received a weight of 63.1 percent in the fixed effects model versus 6.7 percent in the random effects model (Table C in S4 File). Similarly, for well-water consumption, the RR reported by Taylor et al. [102] (RR = 0.93; 95% CI = 0.88–0.98) drove the fixed effects meta-analysis value for Tier 1 and Tier 2 studies combined (RR = 1.02; 95% CI = 0.98–1.07), because this RR was assigned a weight of 68.8 percent (Table D in S5 File). In contrast, a 4.7 percent weight was given to this RR in the random effects model. Likewise, the RR for farming from the Tier 2 study by Lee et al. [148] (RR = 0.86; 95% CI = 0.81–0.92) had a large impact (27.9%) on the fixed effects RR and less of an effect (4.8%) on the random effects RR for Tier 1 and Tier 2 studies combined (Table E in S5 File).

The overall estimate of the association between rural living and PD (Fig B in S2 File) was insensitive to stratification by study characteristics, with fixed effects model estimates having generally lower RRs and narrower 95% CIs. In the case of well-water consumption (Fig C in S2 File), fixed effects estimates of the RR tended to be centered around the null, whereas random effects estimates were generally greater than 1.0, but 95% CIs were wide. RR estimates of the association between farming and PD were in general statistically significantly greater than 1.0, independent of the statistical model or after stratification by study characteristics (Fig D in S2 File).

The weight-of-the-evidence assessment of Tier 1 studies on the association between PD and rural living, farming or well-water consumption, according to Bradford Hill’s viewpoints, indicates that there are inadequate data to reach a conclusion of causality (Table 5). The associations reported were small and the biological gradient and the temporality of disease onset have not been adequately investigated. The results are inconsistent for rural living (Fig 3). Rural living, well-water consumption and farming lack specificity.

### Pesticide Use

The RRs for the association between use of any pesticide and PD were not statistically significantly correlated with RRs for farming (r = 0.07; p = 0.72; 33 studies) or herbicide use (r = 0.11; p = 0.74; 12 studies). Positive, but not statistically significant, correlations with pesticide use were observed for rural living (r = 0.39; p = 0.12; 17 studies), well-water use (r = 0.34; p = 0.12; 22 studies) and fungicide use (r = 0.66; p = 0.10; 7 studies). There were statistically significant positive correlations between the RRs for use of any pesticide and use of insecticides (r = 0.82; p = 0.001; 12 studies) or paraquat (r = 0.84; p = 0.005; 9 studies).

**Fig 6:**
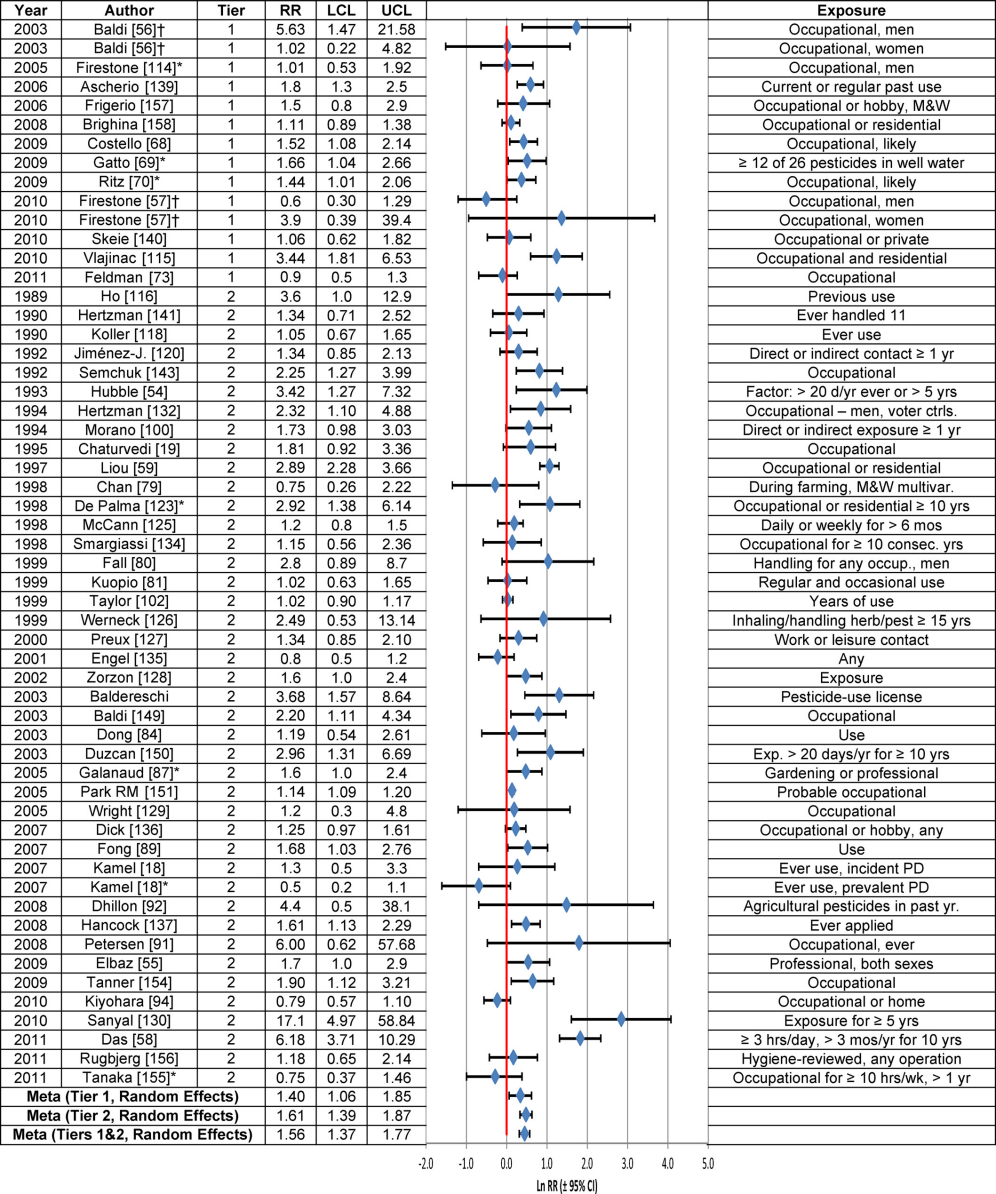
Association between pesticide use and Parkinson’s disease. The natural logarithm of the estimated relative risk [ln(RR)] and the 95% confidence interval for each study are displayed (see the legend for Fig 1 for instructions on how to interpret forest plots). An asterisk (*) denotes RR estimates that are not included in the meta-analysis due to study overlap with another RR estimate shown in the figure. RR = relative risk, LCL = lower limit of the 95% confidence interval, UCL = upper limit of the 95% confidence interval. Citations for studies appearing in this figure can be found here: [18, 19, 54–59, 68–70, 73, 79–81, 84, 87, 89, 91, 92, 94, 100, 102, 107, 114–116, 118, 120, 123, 125–130, 132, 134–137, 139–141, 143, 149–151, 154–158].

Forty-nine of the 56 RRs of the association between the use of any pesticide and PD were greater than 1.0, with 24 RRs being statistically significant. Only seven studies had RRs less than 1.0 and none was statistically significant (Fig 6). There were 11 Tier 1 studies and 38 Tier 2 studies among the 49 studies that reported independent estimates of the RR for pesticide use. The association between pesticide use and PD was statistically significant using the fixed effects model for Tier 1 studies (RR = 1.32; 95% CI = 1.16–1.52) and for all studies combined (RR = 1.22; 95% Cl = 1.18–1.27) (Table 3). Using the random effects model, the RRs were also statistically significant, and slightly greater than the fixed effects RRs for Tier 1 studies (RR = 1.40; 95% Cl = 1.06–1.85) and all studies (RR = 1.56; 95% Cl = 1.37–1.77). When corrected for publication bias, the association between pesticide use and PD was statistically significant for Tier 1 studies using the fixed effects model (RR = 1.14; 95% Cl = 1.01–1.29), but it was not statistically significant when the random effects model was used (RR = 1.11; 95% Cl = 0.82–1.50). The meta-analysis estimates were insensitive to stratification by study characteristics, but the random effects model tended to produce larger RRs with wider 95% CIs than the fixed effects model (Fig E in S2 File).

The weight-of-the-evidence assessment of Tier 1 studies indicated there was a consistent positive association between pesticide use and PD (Table 5). The results lacked specificity or biological plausibility. Neither the biological gradient nor the latency period until PD diagnosis following pesticide use was adequately assessed.

### Herbicide, Fungicide and Insecticide Use

Herbicide, fungicide and insecticide data are presented together in this section, because for most crops, it is likely that a grower would have applied more than one of these classes of pesticide sometime during the growing season, thereby leading to a correlation between exposures and, potentially, their corresponding RRs. Statistically significant positive correlations were observed between RRs for herbicide and insecticide use (r = 0.66; p = 0.008; 15 studies) and those for fungicide and insecticide use (r = 0.90; p = 0.001; 9 studies). A positive correlation between the RRs for herbicide and fungicide use (r = 0.58; p = 0.08; 10 studies) and a negative correlation between the RRs for paraquat and herbicide use (r = - 0.61; p = 0.14; 7 studies) were observed, but these correlations were not statistically significant.

**Fig 7:**
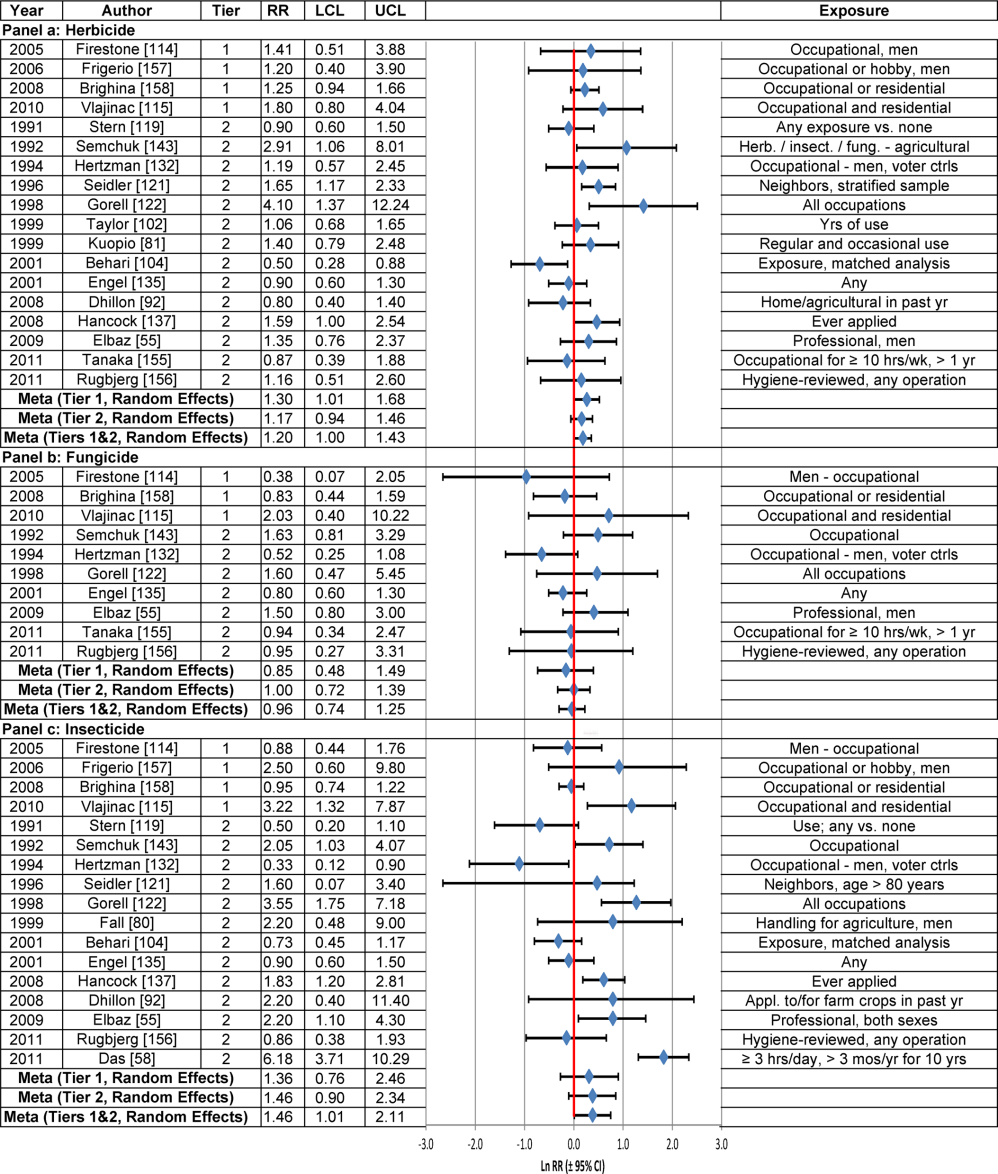
Association between herbicide (Panel a), fungicide (Panel b) or insecticide use (Panel c) and Parkinson’s disease. The natural logarithm of the estimated relative risk [ln(RR)] and the 95% confidence interval for each study are displayed (see the legend for Fig 1 for instructions on how to interpret forest plots). RR = relative risk, LCL = lower limit of the 95% confidence interval, UCL = upper limit of the 95% confidence interval. Citations for studies appearing in this figure can be found here: [55, 58, 80, 81, 92, 102, 104, 114, 115, 119, 121, 122, 132, 135, 137, 143, 155–158].

Use of herbicides (RR = 1.20; 95% CI = 1.06–1.36) or insecticides (RR = 1.32; 95% CI = 1.14–1.52) was associated with statistically significantly increased PD risk, based on all Tier 1 and Tier 2 studies using the fixed effects model (Table 3, Fig 7). Similar results were obtained using the random effects model. An evaluation of Tier 1 studies using the fixed effects model also yielded a statistically significant positive association for herbicides (RR = 1.30; 95% CI = 1.01–1.68), but not for insecticides (RR = 1.04; 95% CI = 0.83–1.31). After correction for publication bias, the RRs between use of herbicides, fungicides or insecticides and PD in Tier 1 studies did not change appreciably, but none of them were statistically significant, based on either random or fixed effects models (Table 3).

The results for both herbicides and insecticides were insensitive to stratification by study characteristics. The stratum-specific meta-analysis RRs based on the fixed effects model were similar to those based on the random effects model for herbicides (Fig F in S2 File) and insecticides (Fig H in S2 File), although 95% CIs tended to be wider for insecticides when calculated using the random effects model.

**Fig 8:**
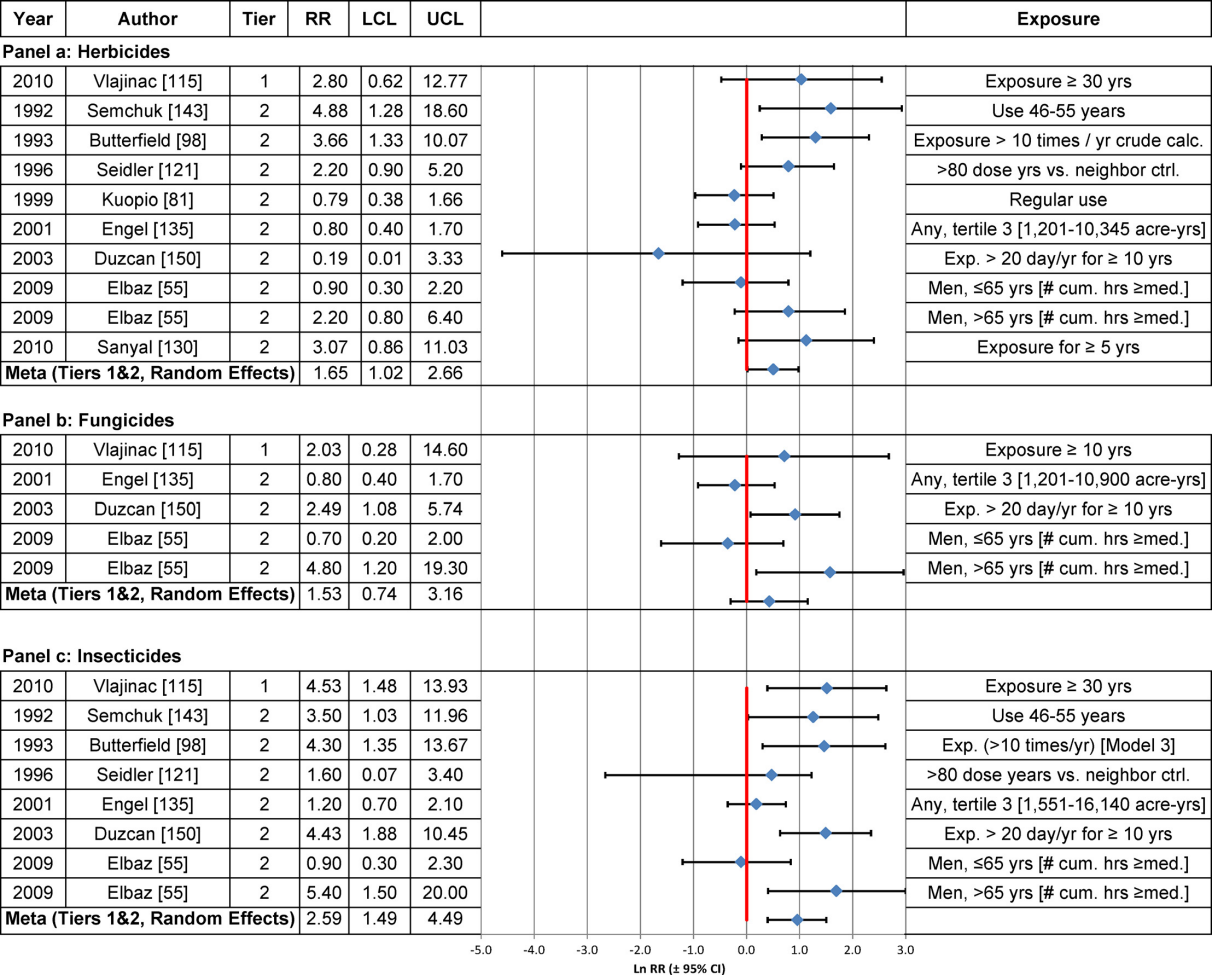
Association between high herbicide (Panel a), high fungicide (Panel b) or high insecticide use (Panel c) and Parkinson’s disease. The natural logarithm of the estimated relative risk [ln(RR)] and the 95% confidence interval for each study are displayed (see the legend for Fig 1 for instructions on how to interpret forest plots). RR = relative risk, LCL = lower limit of the 95% confidence interval, UCL = upper limit of the 95% confidence interval. Citations for studies appearing in this figure can be found here: [55, 81, 98, 115, 121, 130, 135, 143, 150].

High herbicide use was statistically significantly positively associated with PD risk among Tier 1 and Tier 2 studies combined (Table 3, Fig 8 Panel a), but not in the single Tier 1 study (RR = 2.8; 95% CI = 0.6–12.8) conducted by Vlajinac et al. [115], which lacked precision. In contrast, Vlajinac et al. [115] reported a statistically significant positive association between high use of insecticides and PD (RR = 4.5; 95% CI = 1.5–13.9) in the only Tier 1 study of this association, but again, precision in this study was low. High use of insecticides, based upon Tier 1 and Tier 2 studies combined, was statistically significantly associated with PD in both fixed effects and random effects models (Table 3, Fig 8 Panel c).

Use of fungicides (based on Tiers 1 and 2 studies combined or Tier 1 studies alone) and high use of fungicides (based on one Tier 1 study) were not statistically significantly associated with PD risk in any statistical analysis (Table 3, Fig 7 and Fig 8 Panel b). The results were insensitive to use of the fixed effects or random effects model and to stratification by study characteristics (Fig G in S2 File).

The weight-of-the-evidence assessment of Tier 1 studies on the association between herbicide, fungicide or insecticide use and PD, using Bradford Hill’s viewpoints, indicated that there were insufficient high-quality studies to warrant the determination of causation (Table 5). Among the four Tier 1 studies on herbicide use, the overall RRs were comparable in magnitude before and after correction for reporting bias, although the RR was statistically non-significant after correction (uncorrected RR = 1.30; 95% CI = 1.01–1.68; corrected RR = 1.26; 95% CI = 0.99–1.60) (Table 3). Based on the three Tier 1 studies on fungicide use, the RRs were not statistically significant. For insecticides, only one of the four Tier 1 studies [115] had a statistically significantly elevated risk (RR = 3.2; 95% CI = 1.3–7.9) (Fig 7 Panel c).

For all three exposures, the meta-analysis RRs for Tier 1 studies were heavily influenced by the study by Brighina et al. [158], which was assigned a study weight of greater than 75 percent in five of the six fixed and random effects models (Tables G-I in S4 File), and 39 percent in the random effects model for insecticides (Table I in S4 File). Biological gradients and temporality were inadequately assessed for these exposures. The results lack specificity and plausibility [159].

### Paraquat Use

**Fig 9:**
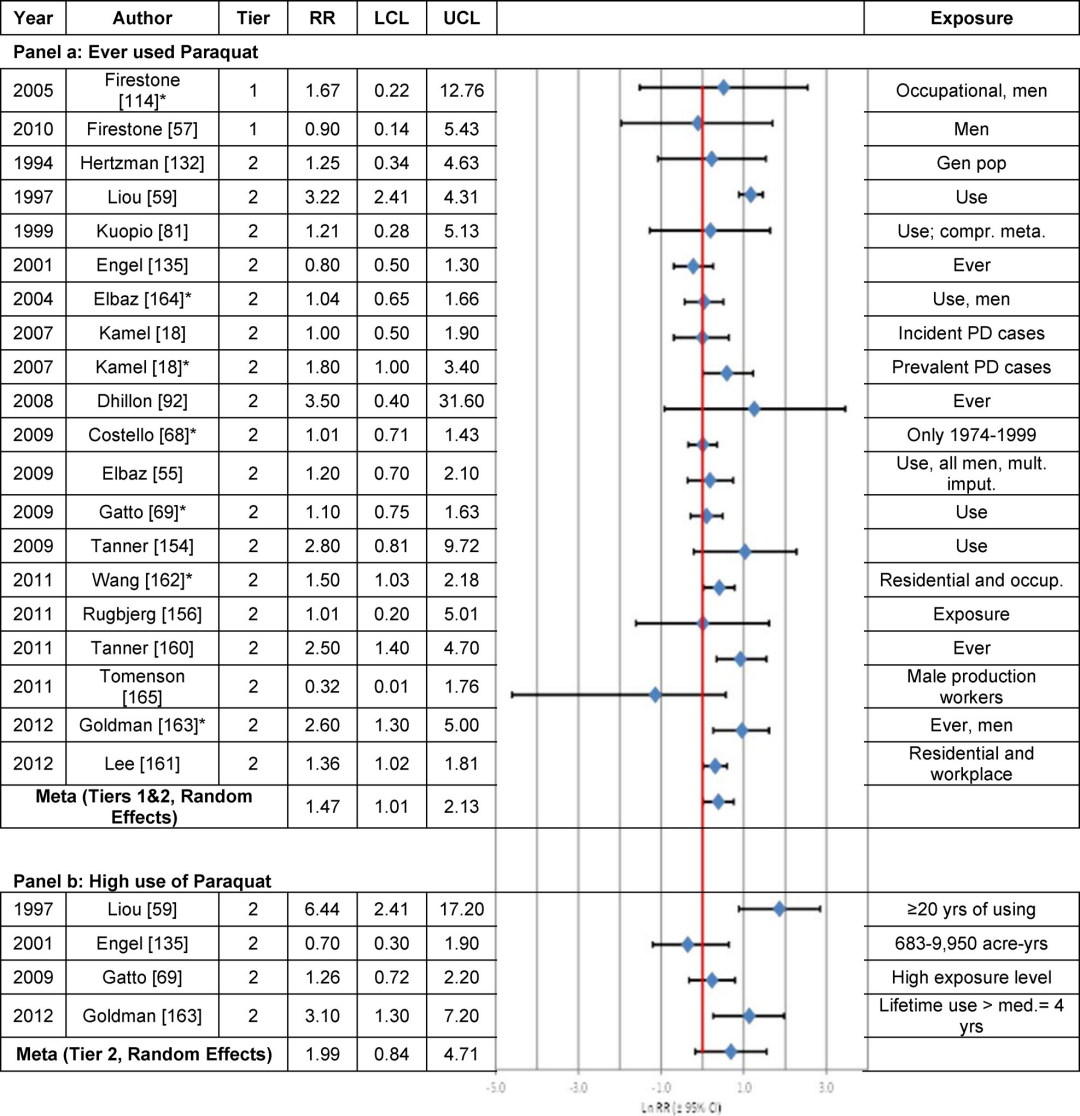
Association between ever use (Panel a) or high use (Panel b) of paraquat and Parkinson’s disease. The natural logarithm of the estimated relative risk [ln(RR)] and the 95% confidence interval for each study are displayed (see the legend for Fig 1 for instructions on how to interpret forest plots). An asterisk (*) denotes RR estimates that are not included in the meta-analysis due to study overlap with another RR estimate shown in the figure. RR = relative risk, LCL = lower limit of the 95% confidence interval, UCL = upper limit of the 95% confidence interval. Citations for studies appearing in this figure can be found here: [18, 55, 57, 59, 68, 69, 81, 92, 114, 132, 135, 154, 156, 160–165].

The one independent Tier 1 study by Firestone et al. [57] found no association between paraquat use and PD (RR = 0.9; 95% CI = 0.14–5.43) (Fig 9 Panel a). Three of the 12 (25%) independent Tier 2 studies [59, 160, 161] reported a statistically significantly elevated risk of PD in individuals who used paraquat. There was a statistically significantly positive meta-analysis RR between PD and paraquat use based on the fixed effects model for all studies (RR = 1.69; 95% CI = 1.44–1.98) (Table 3). Similar results were obtained using the random effects model (RR = 1.47; 95% CI = 1.01–2.13). Results were comparable when overlapping estimates were included from four publications from a case-control study based in California [68, 69, 161, 162], two publications from a nested case-control study in the Agricultural Health Study [160, 163], two publications from a case-control study in Washington [57, 114], and two publications from a case-control study of agriculture workers in France [55, 164], as well as estimates for incident and prevalent PD from the Agricultural Health Study [18] (20 estimates; RRs shown in Fig 9 Panel a).

Among the four studies (all Tier 2) that evaluated the association between high use of paraquat and PD risk (Fig 9 Panel b), two of the studies [59, 163] reported statistically significant positive RRs. Overall, the fixed effects meta-analysis RR was statistically significant (RR = 1.75; 95% CI = 1.19–2.57) (Table 3); the random effects RR was not statistically significant (RR = 1.99; 95% CI = 0.84–4.71), although it was comparable in magnitude to the fixed effects RR.

Sensitivity analyses indicated that the meta-analysis RRs stratified by study characteristics were similar to RRs calculated for all Tier 2 studies (Fig I in S2 File). However, the meta-analysis RRs were not statistically significant after stratification by source population (population-based) or control type (cohort), whereas RRs calculated using the fixed effects model were statistically significant regardless of interview type (in-person or other), method of paraquat use ascertainment (self-reported or other) and confounder adjustment (age, gender and cigarette smoking or fewer covariates).

An assessment of the weight of the evidence according to Bradford Hill’s viewpoints (Table 5) indicates there is an inadequate basis to draw an inference of causality between PD and paraquat use based upon one Tier 1 study [57], which was null but imprecise. Biological gradients and temporality were not assessed. Although the hypothesis that paraquat might cause PD is highly specific, there is disagreement concerning biological plausibility [166].

## Discussion

In this study we found statistically significant inverse associations between cigarette smoking and PD. Meta-analysis RRs, after adjustment for publication bias, ranged from 0.54 to 0.55 for current vs. never smokers. The consistency of the association between smoking cigarettes and PD was maintained when the studies were stratified by different study characteristics, including the Tier 1/Tier 2 categories that we defined and factors used by others to assess study quality (i.e., exposure assessment methods, source population, type of controls and degree of confounder adjustment [49, 50]). Our meta-analysis results for cigarette smoking are comparable to those reported by others (Table 6; [37, 39, 40]).

**Table 6:** Comparison of meta-analysis results from this study to results in the published literature^1^: meta-relative risk (RR; random effects model), 95 percent confidence interval (CI) and number of studies (N).

Risk Factor	Hernán et al. [39]^a^	Allam et al. [40]	Priyadarshi et al. [36]	Van Maele-Fabry et al. [29]^b^	van der Mark et al. [28]	Noyce et al. [37]	Ntzani et al. [167]	Pezzoli and Cereda [30]	Allen and Levy [38]	Breckenridge et al. (2016), all studies	Breckenridge et al. (2016), Tier 1 studies, adjusted for publication bias
**Current Smoking vs. Never Smoking**^a^ **(**Fig 1**)**	0.39* (0.32, 0.47) N = 18	0.37* (0.31, 0.45) N = 26	—	—	—	0.44* (0.39, 0.50) N = 33	—	—	—	0.41* (0.34, 0.48) N = 33	0.55* (0.39, 0.78) N = 11
**Heavy/Long-Term Smoking vs. Never Smoking (Fig 2)**	—	—	—	—	—	—	—	—	—	0.49* (0.43, 0.57) N = 37	0.69* (0.57, 0.83) N = 10
**Rural Living vs. Non-Rural Living (Fig 3)**	—	—	1.56* (1.17, 2.07) N = 16	—	—	1.43* (1.13, 1.81) N = 19	—	1.32* (1.15, 1.51) N = 30	—	1.43* (1.22, 1.69) N = 29	1.52 (0.85, 2.71) N = 4
**Well-Water vs. Non-Well-Water Consumption (Fig 4)**	—	—	1.26 (0.96, 1.64) N = 18	—	—	1.21* (1.04, 1.40) N = 28	—	1.34* (1.16, 1.55) N = 37	—	1.30* (1.12, 1.51) N = 35	1.48* (1.02, 2.15) N = 5
**Farming vs. Non-Farming (Fig 5)**	—	—	1.42* (1.05, 1.91) N = 12	1.23 (0.99, 1.53) N = 8	—	1.26* (1.10, 1.44) N = 25	—	1.30* (1.14, 1.49) N = 34	—	1.24* (1.12, 1.37) N = 48	1.28 (0.99, 1.66) N = 8
**Pesticide Use vs Non-Use (Fig 6)**	—	—	1.85* (1.31, 2.60) N = 14	1.52 (1.00, 2.32) N = 8	1.62* (1.40, 1.88) N = 39	1.78* (1.50, 2.10) N = 38	1.49*^c^ (1.28, 1.73) N = 26	1.76* (1.56, 2.04) N = 51	1.63* (1.37, 1.93) N = 29	1.56* (1.37, 1.77) N = 49	1.11 (0.82, 1.50) N = 11
**Herbicide Use vs. Non-Use (Fig 7A)**	—	—	—	0.88 (0.60, 1.29) N = 2	1.40* (1.08, 1.81) N = 14	—	—	1.33* (1.08, 1.65) N = 19	1.29* (1.04, 1.60) N = 13	1.20* (1.00, 1.43) N = 18	1.26 (0.99, 1.60) N = 4
**Fungicide Use vs. Non-Use (Fig 7B)**	—	—	—	2.0 (0.33, 13.44) N = 2	0.99 (0.71, 1.40) N = 9	—	—	0.97 (0.69, 1.38) N = 12	0.77 (0.51, 1.16) N = 5	0.96 (0.74, 1.25) N = 10	0.85 (0.48, 1.49) N = 3
**Insecticide Use vs. Non-Use (Fig 7C)**	—	—	—	—	1.50* (1.07, 2.11) N = 14	—	—	1.53* (1.12, 2.08) N = 18	1.45* (1.06, 1.97) N = 12	1.46* (1.01, 2.11) N = 17	1.36 (0.76, 2.45) N = 4
**Paraquat Use vs. Non-Use (Fig 9A)**	—	—	—	0.78 (0.49, 1.24) N = 2	—	—	1.32*^d^ (1.10, 1.60) N = 9	2.19* (1.48, 3.26) N = 7	1.38 (0.72, 2.66) N = 6	1.51* (1.04, 2.20) N = 12	—

^1^ The results from the published meta-analysis are as reported; no attempt was made to verify the details of the individual studies selected by the authors for inclusion.

^*^ Statistically significant (P < 0.05; 95% CI excludes 1.0).

^a^ Kiyohara and Kusuhara [168] reported a meta-analysis RR = 0.31 (95% CI = 0.25, 0.38) for current cigarette smokers (N = 24 studies); RR = 0.55 (95% CI = 0.51, 0.59) for ever smokers (N = 50 studies) and RR = 0.77 (95% CI = 0.63–0.83) for former smokers (N = 22 studies).

^b^ Cohort studies only.

^c^ The meta-RR was reported in the text as 1.58 (95% CI = 1.35, 2.85) but shown in a figure as 1.49 (95% CI = 1.28, 1.73).

^d^ The meta-RR was described in the text as being estimated from a random effects model but in a figure as being estimated from a fixed effects model.

The strength and consistency of the weight of evidence suggest that there is an inverse causal relationship between cigarette smoking and PD. Hernán et al. [61] and Liu et al. [74] each evaluated the combined population in two large prospective cohorts, and found that among current cigarette smokers, the strength of the inverse association between cigarette smoking and PD significantly increased with the number of cigarettes smoked per day. Among past smokers, Hernán et al. [61] reported that there was a significant decrease in the strength of the association with a greater amount of time since having quit smoking. In spite of this strong and consistent evidence, the association between cigarette smoking and PD risk lacks specificity given that cigarette smoke comprises many structurally diverse chemicals. Mechanisms underlying the potential neuroprotective effect of cigarette smoke have been postulated, including the activation of nicotinic receptors [169] or cigarette smoke-induced decreased rate of formation of potentially neurotoxic metabolites of endogenous agents [170]. To date, no constituent of cigarette smoke or any other agent has been identified as being neuroprotective against PD [112].

Recently, it has been postulated that individuals who will develop PD find it easier to quit cigarette smoking than individuals who will not develop PD [171]. According to this hypothesis, the consistent inverse association between cigarette smoking and PD is due to reverse causation, whereby prodromal disease leads to an effect on smoking behavior. Under this hypothesis, cigarette smoking per se is not neuroprotective, but rather cigarette smoking occurs less frequently in PD patients, perhaps through early impairment in the quality of olfaction [2] or dysfunction of dopaminergic reward circuitry [172, 173]. To date, no specific genetic or psychological factor has been identified that might prevent individuals who are prone to develop PD from engaging in cigarette smoking and no factor has been identified that might make it easier for undiagnosed PD patients to quit cigarette smoking.

In our study, random effects meta-analysis results for Tier 1 and Tier 2 studies combined were statistically significant for all agricultural lifestyle factors and types of pesticide used (Table 6). Excluding fungicides, based on Tier 1 studies alone, RRs were all greater than unity but were not statistically significant, except for rural living. RRs from individual studies were considerably less consistent than observed in studies on cigarette smoking. Correction for publication bias and stratification by study characteristics had little impact, although RRs were attenuated in Tier 2 studies compared with Tier 1 RRs. Our combined-studies results were comparable to those reported by other investigators (Table 6). The exception was the study by Van Maele-Fabry et al. [29], who evaluated only cohort studies (N = 2 to 8). In their study, RRs for farming and the use of pesticides, herbicides, fungicides or paraquat were not statistically significant.

Studies of farming occupation as a risk factor rarely distinguish among the diverse tasks and the variety of chemical and pathogen exposures that may occur in different types of farming activities. For example, farming may result in many other exposures, such as an increased likelihood of head injury [174], vibrational stress [175], infection [176] and soil-borne pathogens [54, 177–179], that could be causally related to PD. Individuals engaged in these occupations may also be exposed to a large number of chemicals including herbicides, fungicides, insecticides, rodenticides, fumigants, fertilizers and fuels. Participants in such occupations may also exhibit unique lifestyle factors such as dietary preference, tobacco use or sunlight exposure that could alter their susceptibility to PD [33]. Similarly, rural living and well-water consumption lack specificity and correspond to a wide range of exposures, some of which may plausibly be causally linked to PD.

Positive, statistically significant associations (uncorrected RRs = 1.2 to 1.6) were observed between pesticide use and PD. Correction for publication bias reduced the strength of the association (corrected RRs = 1.1) and rendered the result non-significant based upon the random effects model but not the fixed effects model. Similar results were obtained for use of herbicides and insecticides, whereas use of fungicides was not statistically significantly associated with PD in any model. Stratification by study quality or other characteristics had little impact on the magnitude of the RRs, although the stratified RRs were often based on few studies and, therefore, the RRs were often statistically non-significant. The analysis of biological gradient (high use of herbicides, fungicides or insecticides vs. none) was limited by the number of available studies.

Pesticides are a broad group of chemicals that are structurally and functionally diverse and may not share a common mechanism of action, toxicity [159] or common use. The idea that “pesticides,” or even all herbicides, fungicides or insecticides as a class, could cause PD is inconsistent with the understanding that the mechanism of toxicity is highly specific to the chemical structure and the biochemical pathway(s) perturbed by a causative agent [180]. Although a specific pesticide or its metabolites within any of these categories could be causally related to PD, no such compound has yet been identified. Statistically significant correlations observed between RRs derived from the same studies (e.g., pesticide use and insecticide use [r = 0.82]; pesticide use and paraquat use [r = 0.84]; herbicide use and insecticide use [r = 0.66]; and insecticide use and fungicide use [r = 0.90]) illustrate the lack of specificity among these factors.

It is also plausible that a factor correlated with pesticide use, such as exposure to a highly active specific pesticide or some other factor associated with farming, could be causally related to PD. However, the available data do not permit the identification of such a factor. Biologically plausible mechanisms for PD causation have been postulated for specific pesticides, such as the inhibition of mitochondrial complex I by rotenone [44], the inhibition of aldehyde dehydrogenase by the dithiocarbamate fungicides ferbam, macozeb and maneb [42, 43] and the induction of oxidative stress by paraquat [181, 182]. There is disagreement on the utility of animal models for predicting risk in humans [183–185], who may have had limited or intermittent exposure to these pesticides. Furthermore, results obtained using some models appear to be less reliable [186, 187] than originally reported [181, 182], and there is a paucity of epidemiological data for most specific pesticides.

When we focused on the 20 published RR estimates (from 13 independent study populations) on paraquat and PD, we found statistically significant positive associations overall, but not in some strata of study characteristics. For example, the one Tier 1 study by Firestone et al. [57] did not find an association between paraquat and PD. Studies that assessed the association between paraquat exposure and a second potential risk factor for PD, such as co-exposure to the fungicide maneb [68], the occurrence of head injury [161] or the presence of genetic susceptibility factors [70, 163], were not included because of limited data. Overall, the epidemiological data are inconsistent across studies, and collectively, they do not support a conclusion that a causal relationship exists between exposure to paraquat and PD.

Statistical associations between PD and exposure to pesticides or factors related to rural or agricultural living do not necessarily indicate cause-and-effect relationships. Consideration must be given to alternative explanations such as random error (chance), systematic error (bias) and confounding, as well as to the issues of validity, precision and reliability. Sir Austin Bradford Hill developed “viewpoints” to evaluate whether an association between an exposure and an outcome was likely to be causal [188]. He asked, “What aspects of that association should we especially consider before deciding that the most likely interpretation of its causation?” ([188], p.295). Other approaches for evaluating causality have been proposed [189–192], but the Bradford Hill viewpoints are often used. Based upon our weight-of-the-evidence assessment using Bradford Hill’s viewpoints, we conclude that none of the risk factors that we assessed provide sufficient evidence of a causal relationship with PD. This conclusion is consistent with those of other investigators who conducted systematic reviews and/or meta-analyses (Table 7).

**Table 7:** Conclusions from published systematic reviews and meta-analyses of epidemiology studies on Parkinson’s disease.

Study	Type of Analysis	Conclusion
**Barbeau et al. [24]**	**Case Study**	“In summary, we demonstrate that both environmental and genetic susceptibility factors play an important role in the pathogenesis of PD. The prevalence of PD varies considerably between hydrographic basins in Quebec, with the highest prevalence rates being found in rural commercial agricultural areas of high pesticide use. It should be emphasized, however, that pesticides are only *one* of the numerous environmental contaminants present in the same region and that no one has yet *proved* the cause-effect relationship of parkinson’s disease with pesticides.”
**Priyadarshi et al. [36]**	**Meta-Analysis**	“Dose-response relationships could not be established due to the imprecise nature of the reported data. Our findings suggest that living in a rural area, drinking well water, farming, and exposure to pesticides may be a risk factor for developing PD:”
**Li et al. [26]**	**Review**	“Epidemiologic studies were considered according to study quality parameters, and results were found to be mixed and without consistent exposure-response or pesticide-specific patterns. These epidemiologic studies were limited by a lack of detailed and validated pesticide exposure assessment … We conclude that the … epidemiologic data reviewed do not provide sufficient evidence to support a causal association between pesticide exposure and PD.”
**Brown et al. [27]**	**Review**	“[T]he epidemiology … studies were limited by methodologic weaknesses … At present, the weight of the evidence is sufficient to conclude that a generic association between pesticide exposure and PD exists but is insufficient for concluding that this is a causal relationship or that such a relationship exists for any particular pesticide compound or combined pesticide and other exogenous toxicant exposure.”
**Wirdefeldt et al. [33]**	**Review**	“Despite a vast literature on lifestyle and environmental possible risk or protective factors, consistent findings are few. There is compelling for protective effects of smoking and coffee, but the biologic mechanisms for these possibly causal relations are poorly understood. … Evidence that one or several pesticides increase PD risk is suggestive but further research is needed to identify specific compounds that may play a causal role. … Future epidemiologic studies of PD should be large, include detailed quantifications of exposure, and collect information on environmental exposures as well as genetic polymorphisms.”
**van der Mark et al. [28]**	**Meta-Analysis**	“This review affirms the evidence that exposure to herbicides and insecticides increase the risk of PD. Future studies should focus on more objective and improved methods of pesticide exposure assessment.”
**Van Maele-Fabry et al. [29]**	**Meta-Analysis**	“The present study provides some support for the hypothesis that occupational exposure to pesticides increases the risk of PD.”
**Freire and Koifman [34]**	**Review**	“Taken together, this comprehensive set of results suggests that the hypothesis of an association between pesticide exposure and PD cannot be ruled out. However, inadequate data on consistent responses to exposure hinder the establishment of a causal relationship with PD.”
**Noyce et al. [37]**	**Meta-Analysis**	“[P]ositive significant associations were found for history of anxiety or depression, pesticide exposure, head injury, rural living, beta-blockers, farming occupation, and well-water drinking … The strongest risk factors associated with later PD diagnosis are having a family history of PD or tremor, a history of constipation, and lack of smoking history. Further factors also but less strongly contribute to risk of PD diagnosis or, as some premotor symptoms, require further standardized studies to demonstrate the magnitude of risk associated with them.”
**Moretto and Colosio [35]**	**Review**	“Available measurements or estimates of human exposure levels that are significantly lower than those used in animal experimentation provide little support for a causal correlation between pesticide exposure and development of PD in humans.”
**Pezzoli and Cereda [30]**	**Meta-Analysis**	“The literature supports the hypothesis that exposure to pesticides or solvents is a risk factor for PD. Further prospective and high-quality case-control studies are required to substantiate a cause-effect relationship.”
**Allen and Levy [38]**	**Meta-Analysis**	“The results of the meta-analysis reported in this study suggest the existence of a statistically positive association between PD and pesticide exposure. The majority of the studies that were pooled in the meta-analysis were case-control design with very few cohort studies and most with poor exposure characterization.”

A comparison of the results from our meta-analysis with those in the published literature (Table 6) indicates that there is general agreement among studies. Overall, cigarette smoking is consistently inversely associated with PD [37, 39, 40], whereas positive associations have been reported PD between and rural living, well-water consumption, farming or pesticide use [28–30, 36, 37]. As expected, the RRs were more likely to be statistically significant when the meta-analysis included more studies (e.g., Noyce et al. [37], Pezzoli and Cereda [30], and Breckenridge et al., 2016 [all studies]) than when fewer studies were included (e.g., Van Maele-Fabry et al. [29] [cohort studies] and Breckenridge et al., 2016 [Tier 1 studies]).

There are limitations to meta-analysis, especially as applied to observational epidemiology [193]. Such limitations are exemplified by the fact that the heterogeneity of results in Tier 1 and Tier 2 studies, as measured by I^2^ in our analyses, was statistically significant for all risk factors except for fungicides. Between-study variances (τ^2^) calculated based on the random effects model and a review of the distribution of RRs permitted the identification of individual studies that contributed the most to such heterogeneity. However, detailed evaluation of the study protocols and individual study records, which usually are not available in publications or directly from investigators, would be necessary before one could decide if there was a scientific basis for either excluding or assigning extraordinary weight to a study that deviates widely from the majority of studies. This unresolvable problem raises questions about the scientific merit of calculating a single summary RR for a set of observational epidemiologic studies.

Statistically significant heterogeneity was observed in Tier 1 studies alone and in Tiers 1 and 2 studies combined for several risk factors (i.e., current smoking, heavy or long-term smoking, rural living, well-water consumption, any pesticide use and insecticide use). Thus, for these risk factors, study heterogeneity did not depend on study categorization. In contrast, we found that while heterogeneity was statistically significant for combined Tiers 1 and 2 studies of farming and herbicide use, I^2^ was not statistically significant for Tier 1 studies alone, suggesting that differences in heterogeneity of results between studies was dependent on the study category. The observation that τ^2^ was usually smaller for Tier 1 studies than for Tier 1 and Tier 2 studies combined suggests, from a statistical perspective, that Tier 1 studies should not be combined with Tier 2 studies for these risk factors.

In our sensitivity analysis we compared the effect on the meta-analysis RRs of using criteria that other investigators have used to classify studies (i.e., exposure assessment method, source population, control type and confounder adjustment) with the criteria that we selected to define study tiers (i.e., enrollment of incident vs. prevalent PD cases, individual vs. ecological exposure assessment, and the basis of PD diagnosis). We found that these different study characteristics did not substantially alter the results of the overall meta-analysis.

It is known that the selection of studies and results for publication, and hence their use in meta-analysis, can be biased [31]. In this evaluation, there was evidence of reporting bias for heavy/long-term cigarette smoking. Furthermore, the practice of combining results from several studies into one estimate of risk may be inappropriate under the component cause model, where there are potentially multiple causal factors [194]. Using meta-analysis for causal inference based up observational studies generally is not warranted because it tends to give a false impression of the consistency of studies [195]. As Greenland pointed out, “no statistical technique can compensate for fundamental limitations of the input data” [196]. Greenland suggested that meta-analysis can be used appropriately as a basis for comparing results from a variety of studies, and not as a method to produce a single RR estimate with narrower CIs. There may be a greater benefit from examining sources of heterogeneity among study results than by merely computing a summary estimate [31]. In addition, it is important to examine studies to ensure that within-study selection bias is minimized, particularly when the results from a post-hoc subgroup analysis is the main focus of the study [197, 198].

Meta-analysis artificially increases precision by combining RR estimates from epidemiological studies [27–30, 36, 37, 39, 40, 111, 199]. In spite of the increased statistical power arising from the combination of studies in meta-analysis, the results reported in this paper and by other researchers are consistent with each other only with respect to cigarette smoking, rural living, well-water consumption and pesticide use when we analyzed Tier 1 studies. Contradictory evidence for rural living has been reported in a large study of US Medicare beneficiaries aged 65 years and older. In this study, the incidence and prevalence of PD were statistically significantly greater in urban (population greater than 1 million) than in rural (population less than 2500) communities [200]. However, in general, our meta-analysis results were comparable to those published by other investigators, with mostly minor differences in meta-analysis RRs that were probably due to slight differences in the selection of studies and RR estimates.

Increasing statistical power by combining studies cannot overcome problems in the design and conduct of the individual studies. Failure to adjust for study deficiencies may be responsible, at least in part, for many false positive findings in the published epidemiological literature [201–205]. The need for higher standards and a more critical appraisal of individual studies was recognized in a series of recommendations for Strengthening the Reporting of Observational Studies in Epidemiology (STROBE), which were developed to address transparency in reporting the results of epidemiological studies [206].

To establish a causal role of any factor in the etiology of PD, particularly those related to rural living and farming, substantial improvements are needed in the design and conduct of observational epidemiologic studies. Future research must 1) better characterize specific past exposure to suspected agents to permit a more accurate assessment of the dose-response relationship; 2) utilize neurologists or movement disorder specialists to diagnose PD and to confirm the absence of diseases in controls in order to minimize disease misclassification; 3) determine with greater accuracy the date of onset of PD so that the latency between exposure and PD onset and progression can be assessed; and 4) enroll incident PD cases close to the time of diagnosis (if not onset) to increase the likelihood that reported exposures preceded disease development. Without improvements in methodology, further research will encounter similar shortcomings, and the identification of causal environmental risk factors for idiopathic PD will not be achieved.

## Supporting Information

S1 Appendix Literature search methods. (PDF)

S2 Appendix PRISMA Flow Chart: Study Identification, Screening, Eligibility, Inclusion and Exclusion. (PDF)

S1 File **Figs A to J: Frequency distribution of the relative risk estimates (RRs) from individual epidemiological studies assessing the association between risk factors and Parkinson’s disease.** Fig A: Frequency distribution of individual study relative risk estimates: Current cigarette smoking. Fig B: Frequency distribution of individual study relative risk estimates: Heavy or long-term smoking. Fig C: Frequency distribution of individual study relative risk estimates: Rural living. Fig D: Frequency distribution of individual study relative risk estimates: Well-water consumption. Fig E: Frequency distribution of individual study relative risk estimates: Farming. Fig F: Frequency distribution of individual study relative risk estimates: Pesticide use. Fig G: Frequency distribution of individual study relative risk estimates: Herbicide use. Fig H: Frequency distribution of individual study relative risk estimates: Fungicide use. Fig I: Frequency distribution of individual study relative risk estimates: Insecticide use. Fig J: Frequency distribution of individual study relative risk estimates: Paraquat use.
(PDF)

S2 File **Figs A to I: Sensitivity analyses of estimated relative risks (RRs) stratified by study characteristics (study quality tier, exposure interview technique, source population, type of controls and extent of confounder adjustment) in fixed and random effects models.** Fig A: Sensitivity Analyses–Heavy Smoking. Fig B: Sensitivity Analyses–Rural Living. Fig C: Sensitivity Analyses–Well-Water Consumption. Fig D: Sensitivity Analyses–Farming. Fig E: Sensitivity Analyses–Pesticide Use. Fig F: Sensitivity Analyses–Herbicide Use. Fig G: Sensitivity Analyses–Fungicide Use. Fig H: Sensitivity Analyses–Insecticide Use. Fig I: Sensitivity Analyses–Paraquat Use.
(PDF)

S3 File **Tables A to I: Metadata characteristics of Tier 1 and Tier 2 studies on the association between risk factors and Parkinson’s disease.** Table A: Metadata for Tier 1 and Tier 2 studies: Current cigarette smoking. Table B: Metadata for Tier 1 and Tier 2 studies: Heavy or long-term cigarette smoking. Table C: Metadata for Tier 1 and Tier 2 studies: Rural living. Table D: Metadata for Tier 1 and Tier 2 studies: Well-water consumption. Table E: Metadata for Tier 1 and Tier 2 studies: Farming. Table F: Metadata for Tier 1 and Tier 2 studies: Pesticide use. Table G: Metadata for Tier 1 and Tier 2 studies: Herbicide, fungicide or insecticide use. Table H: Metadata for Tier 1 and Tier 2 studies: High herbicide, fungicide or insecticide use. Table I: Metadata for Tier 1 and Tier 2 studies: Paraquat ever use or high use.
(PDF)

S4 File **Tables A to N: Normalized study weights based upon fixed and random effects models and RRs and 95% CIs for individual Tier 1 or Tier 2 studies.** Table A: RRs, 95% CIs and fixed or random effects study weights for Tier 1 or Tier 2 studies: Current cigarette smoking. Table B: RRs, 95% CIs and fixed or random effects study weights for Tier 1 or Tier 2 studies: Heavy or long-term cigarette smoking. Table C: RRs, 95% CIs and fixed or random effects study weights for Tier 1 or Tier 2 studies: Rural living. Table D: RRs, 95% CIs and fixed or random effects study weights for Tier 1 or Tier 2 studies: Well-water consumption. Table E: RRs, 95% CIs and fixed or random effects study weights for Tier 1 or Tier 2 studies: Farming. Table F: RRs, 95% CIs and fixed or random effects study weights for Tier 1 or Tier 2 studies: Pesticide use. Table G: RRs, 95% CIs and fixed or random effects study weights for Tier 1 or Tier 2 studies: Herbicide use. Table H: RRs, 95% CIs and fixed or random effects study weights for Tier 1 or Tier 2 studies: Fungicide use. Table I: RRs, 95% CIs and fixed or random effects study weights for Tier 1 or Tier 2 studies: Insecticide use. Table J: RRs, 95% CIs and fixed or random effects study weights for Tier 1 or Tier 2 studies: High herbicide use. Table K: RRs, 95% Cls and fixed or random effects study weights for Tier 1 or Tier 2 studies: High fungicide use. Table L: RRs, 95% CIs and fixed or random effects study weights for Tier 1 or Tier 2 studies: High insecticide use. Table M: RRs, 95% CIs and fixed or random effects study weights for Tier 1 or Tier 2 studies: Paraquat use. Table N: RRs, 95% CIs and fixed or random effects study weights for Tier 1 or Tier 2 studies: High paraquat use.
(PDF)

S5 File **Tables A to J: Normalized study weights for Tier 1 and Tier 2 studies combined (i.e., all studies) based upon fixed and random effects models and RRs and 95% CLs for all individual studies.** Table A: RRs, 95% CIs and fixed or random effects study weights for all studies: Current cigarette smoking. Table B: RRs, 95% CIs and fixed or random effects study weights for all studies: Heavy or long-term cigarette smoking. Table C: RRs, 95% CIs and fixed or random effects study weights for all studies: Rural living. Table D: RRs, 95% CIs and fixed or random effects study weights for all studies: Well-water consumption. Table E: RRs, 95% CIs and fixed or random effects study weights for all studies: Farming. Table F: RRs, 95% CIs and fixed or random effects study weights for all studies: Pesticide use. Table G: RRs, 95% CIs and fixed or random effects study weights for all studies: Herbicide use. Table H: RRs, 95% CIs and fixed or random effects study weights for all studies: Fungicide use. Table I: RRs, 95% CIs and fixed or random effects study weights for all studies: Insecticide use. Table J: RRs, 95% CIs and fixed or random effects study weights for all studies: Paraquat use.
(PDF)

S1 Table S1 Table: PRISMA Checklist. (PDF)
