# Supplementary material for: Association between Parkinson’s Disease and Cigarette Smoking, Rural Living, Well-Water Consumption, Farming and Pesticide Use: Systematic Review and Meta-Analysis
Source: PLoS One. 2016 Apr 7;11(4):e0151841. doi: 10.1371/journal.pone.0151841 (PMC4824443; doi:10.1371/journal.pone.0151841)
Supplement: S1 Appendix — (PDF) [file pone.0151841.s001.pdf]

S1 Appendix – Literature search methods

Boolean literature search terms used to identify any study published prior to June 2013 that evaluated the associations between Parkinson's disease and rural (or agricultural) risk factors, paraquat use and cigarette smoking are presented in sections 1-3, respectively.

Rural (or agricultural) risk factors and Parkinson's disease

(pesticide OR herbicide, OR insecticide, OR fungicide OR occupation OR occupational OR farmer OR farming OR "stockbreeding" OR orchard OR agriculture OR horticulture OR "pesticide applicator" OR "well water" OR "well-water" OR "unfiltered water" OR rural OR "rural living" OR "rural residency" OR "rural life") AND ("Parkinson" OR "Parkinson's disease" OR "Parkinson Disease" OR "parkinsonism" OR "Parkinson's" OR "parkinsonian") AND (randomized controlled trial[Publication Type] OR (randomized[Title/Abstract] AND controlled[Title/Abstract] AND trial[Title/Abstract]) or trial or "Clinical Trial" [Publication Type] or "cohort studies"[MeSH] OR "case-control studies"[MeSH] OR "cross-sectional studies"[MeSH] OR "epidemiologic studies"[MeSH] OR "follow-up studies"[MeSH] OR "longitudinal studies"[MeSH] OR "prospective studies"[MeSH] OR "retrospective studies"[MeSH] or ((meta-analysis [pt] OR meta-analysis [tw] OR metanalysis [tw]) OR ((review [pt] OR guideline [pt] OR consensus [ti] OR guideline* [ti] OR literature [ti] OR overview [ti] OR review [ti]) AND ((Cochrane [tw] OR Medline [tw] OR CINAHL [tw] OR (National [tw] AND Library [tw])) OR (handsearch* [tw] OR search* [tw] OR searching [tw]) AND (hand [tw] OR manual [tw] OR electronic [tw] OR bibliographi* [tw] OR database* OR (Cochrane [tw] OR Medline [tw] OR CINAHL [tw] OR (National [tw] AND Library [tw]))))) OR ((synthesis [ti] OR overview [ti] OR review [ti] OR survey [ti]) AND (systematic [ti] OR critical [ti] OR methodologic [ti] OR quantitative [ti] OR qualitative [ti] OR literature [ti] OR evidence [ti] OR evidence-based [ti]))) or review) BUTNOT ("Case Reports" [Publication Type] OR editorial [pt] OR comment [pt] OR letter [pt])

Paraquat use and Parkinson's disease

"paraquat" AND ("Parkinson" OR "Parkinson's disease" OR "Parkinson Disease" OR "parkinsonism" OR "Parkinson's" OR "parkinsonian") AND (randomized controlled trial[Publication Type] OR (randomized[Title/Abstract] AND controlled[Title/Abstract] AND trial[Title/Abstract]) or trial or "Clinical Trial" [Publication Type] or "cohort studies"[MeSH] OR "case-control studies"[MeSH] OR "cross-sectional studies"[MeSH] OR "epidemiologic studies"[MeSH] OR "follow-up studies"[MeSH] OR "longitudinal studies"[MeSH] OR "prospective studies"[MeSH] OR "retrospective studies"[MeSH] or ((meta-analysis [pt] OR meta-analysis [tw] OR metanalysis [tw]) OR ((review [pt] OR guideline [pt] OR consensus [ti] OR guideline* [ti] OR literature [ti] OR overview [ti] OR review [ti]) AND ((Cochrane [tw] OR Medline [tw] OR CINAHL [tw] OR (National [tw] AND Library [tw])) OR (handsearch* [tw] OR search* [tw] OR searching [tw]) AND (hand [tw] OR manual [tw] OR electronic [tw] OR bibliographi* [tw] OR database* OR (Cochrane [tw] OR Medline [tw] OR CINAHL [tw] OR (National [tw] AND Library [tw]))))) OR ((synthesis [ti] OR overview [ti] OR review [ti] OR survey [ti]) AND (systematic [ti] OR critical [ti] OR methodologic [ti] OR quantitative [ti] OR qualitative [ti] OR literature [ti] OR evidence [ti] OR evidence-based [ti]))) or review) BUTNOT ("Case Reports" [Publication Type] OR editorial [pt] OR comment [pt] OR letter [pt])

Cigarette smoking and Parkinson's disease

(smok* OR tobacco OR cigar*) AND ("Parkinson" OR "Parkinson's disease" OR "Parkinson Disease" OR "parkinsonism" OR "Parkinson's" OR "parkinsonian") AND (randomized controlled trial[Publication Type] OR (randomized[Title/Abstract] AND controlled[Title/Abstract] AND trial[Title/Abstract]) or trial or "Clinical Trial" [Publication Type] or "cohort studies"[MeSH] OR "case-control studies"[MeSH] OR "cross-sectional studies"[MeSH] OR "epidemiologic studies"[MeSH] OR "follow-up studies"[MeSH] OR "longitudinal studies"[MeSH] OR "prospective studies"[MeSH] OR "retrospective studies"[MeSH] or ((meta-analysis [pt] OR meta-analysis [tw] OR metanalysis [tw]) OR ((review [pt] OR guideline [pt] OR consensus [ti] OR guideline* [ti] OR literature [ti] OR overview [ti] OR review [ti]) AND ((Cochrane [tw] OR Medline [tw] OR CINAHL [tw] OR (National [tw] AND Library [tw])) OR (handsearch* [tw] OR search* [tw] OR searching [tw]) AND (hand [tw] OR manual [tw] OR electronic [tw] OR bibliographi* [tw] OR database* OR (Cochrane [tw] OR Medline [tw] OR CINAHL [tw] OR (National [tw] AND Library [tw]))))) OR ((synthesis [ti] OR overview [ti] OR review [ti] OR survey [ti]) AND (systematic [ti] OR critical [ti] OR methodologic [ti] OR quantitative [ti] OR qualitative [ti] OR literature [ti] OR evidence [ti] OR evidence-based [ti]))) or review) BUTNOT ("Case Reports" [Publication Type] OR editorial [pt] OR comment [pt] OR letter [pt]).
