# Supplementary material for: Association between Parkinson’s Disease and Cigarette Smoking, Rural Living, Well-Water Consumption, Farming and Pesticide Use: Systematic Review and Meta-Analysis
Source: PLoS One. 2016 Apr 7;11(4):e0151841. doi: 10.1371/journal.pone.0151841 (PMC4824443; doi:10.1371/journal.pone.0151841)

S2 Appendix – PRISMA Flow Chart: Study Identification, Screening, Eligibility, Inclusion and Exclusion

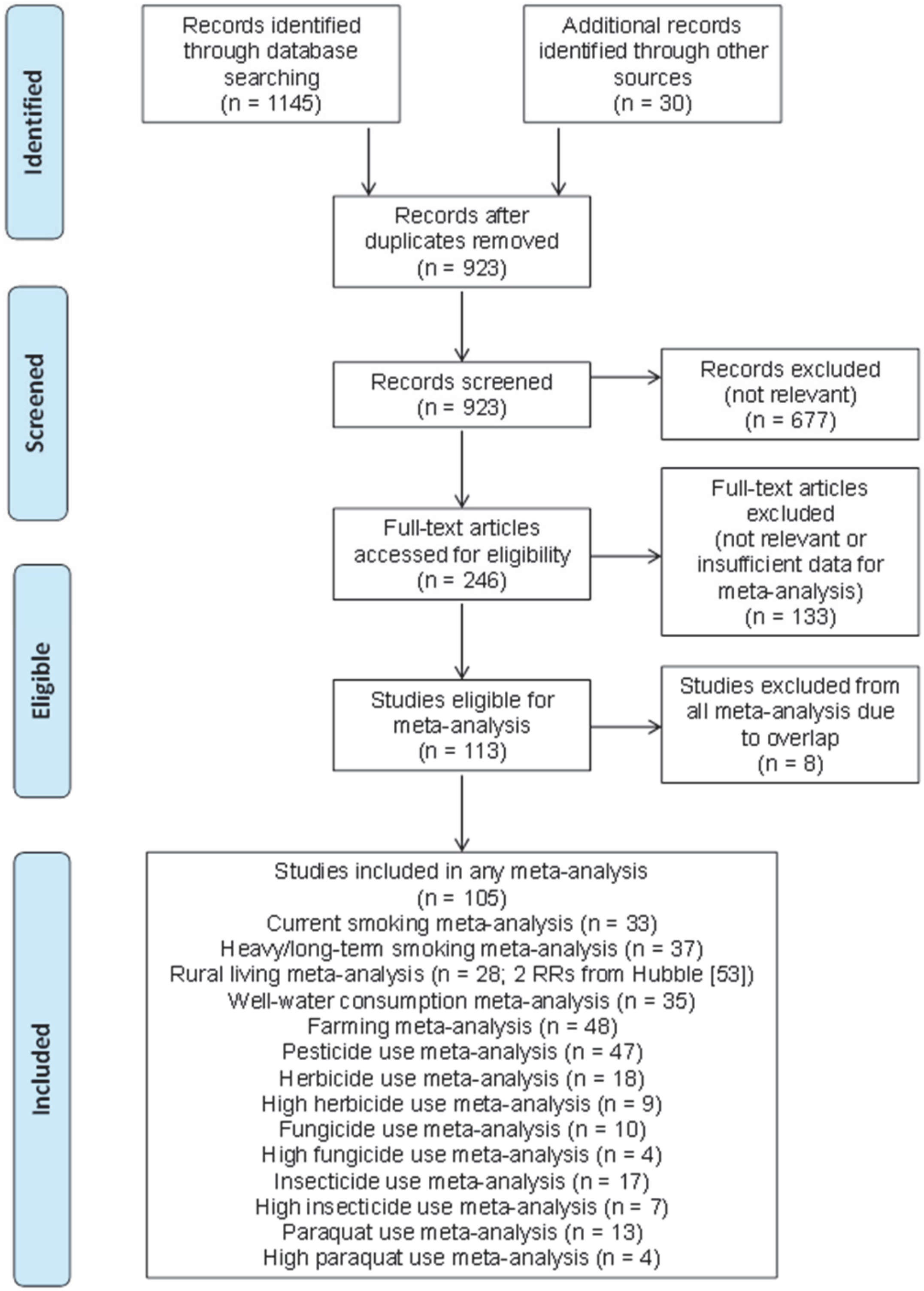

Supplement: S2 Appendix — (PDF) [file pone.0151841.s002.pdf]
