# Supplementary material for: Association between Parkinson’s Disease and Cigarette Smoking, Rural Living, Well-Water Consumption, Farming and Pesticide Use: Systematic Review and Meta-Analysis
Source: PLoS One. 2016 Apr 7;11(4):e0151841. doi: 10.1371/journal.pone.0151841 (PMC4824443; doi:10.1371/journal.pone.0151841)

Figure A: Frequency distribution of individual study relative risk estimates: Current cigarette smoking

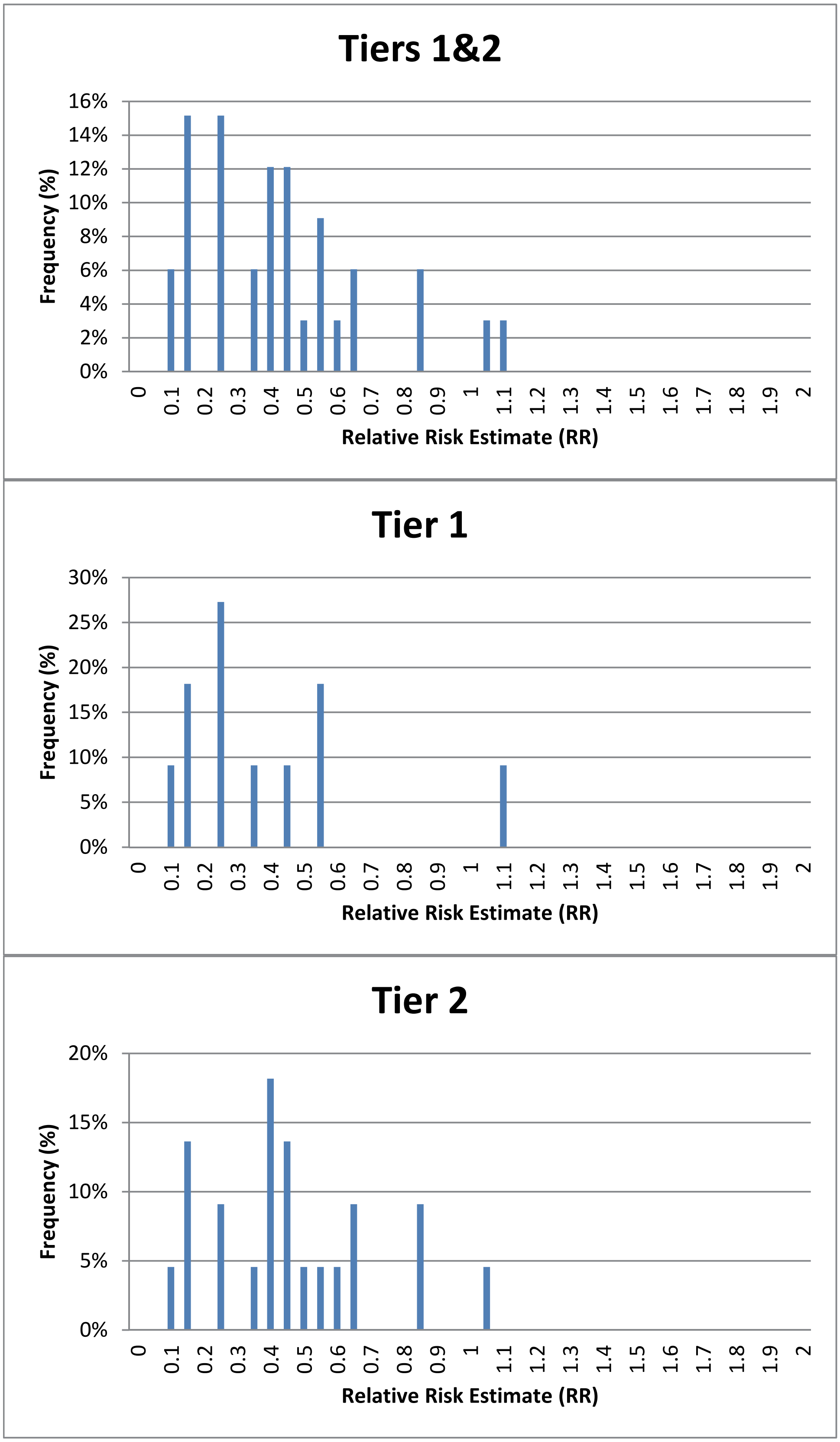

Figure B: Frequency distribution of individual study relative risk estimates: Heavy or long-term smoking

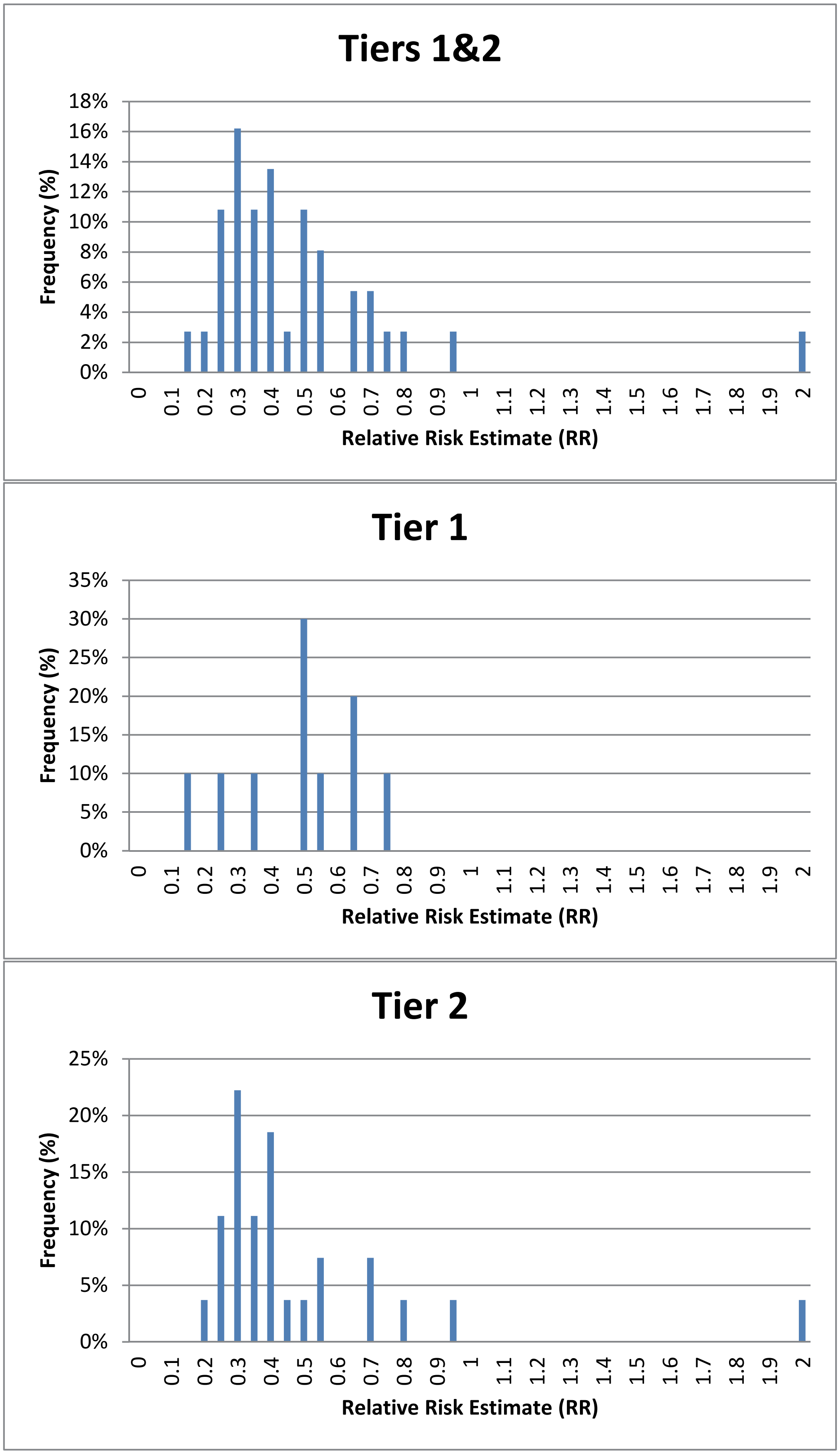

Figure C: Frequency distribution of individual study relative risk estimates: Rural living

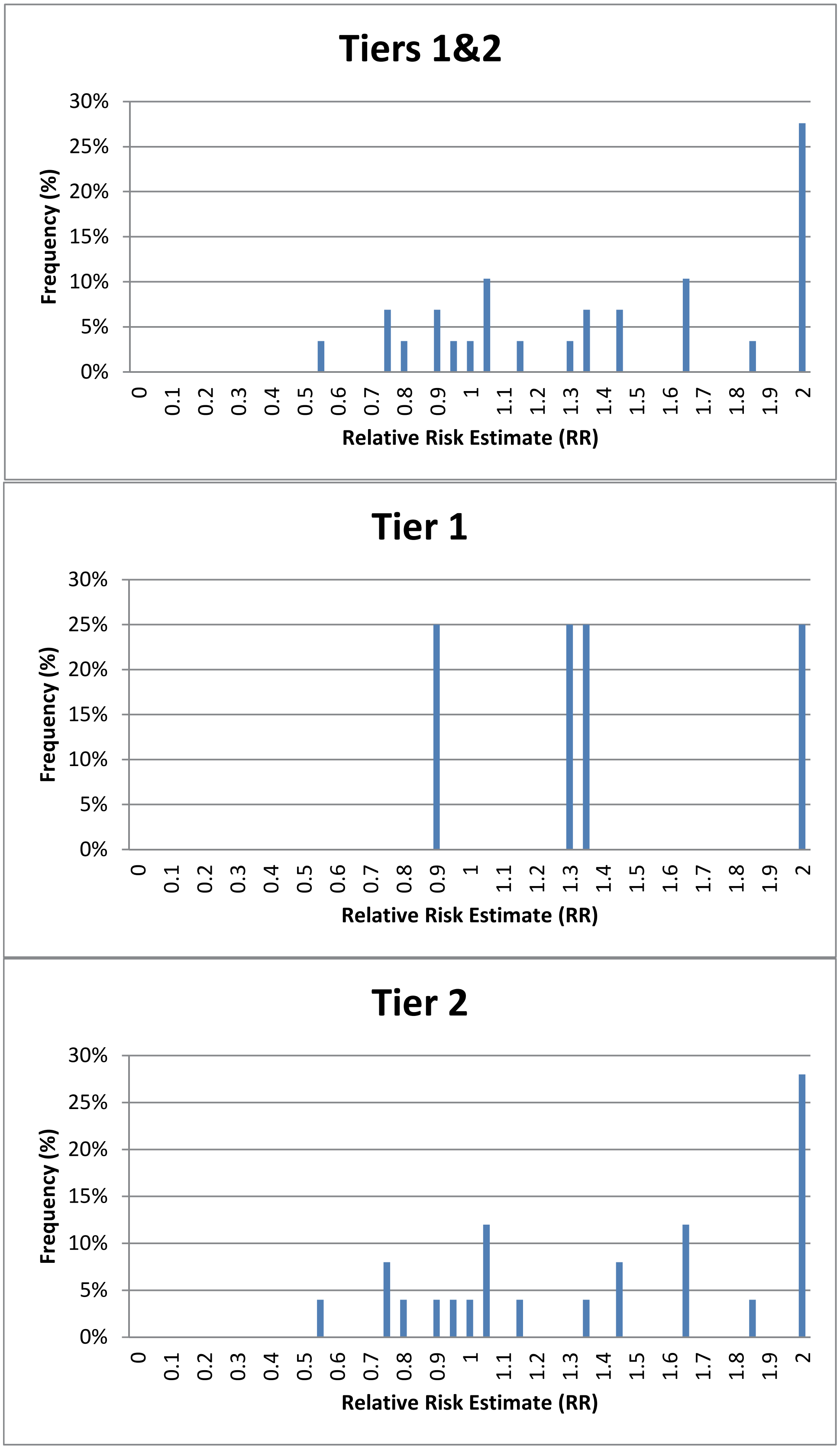

Figure D: Frequency distribution of individual study relative risk estimates: Well-water consumption

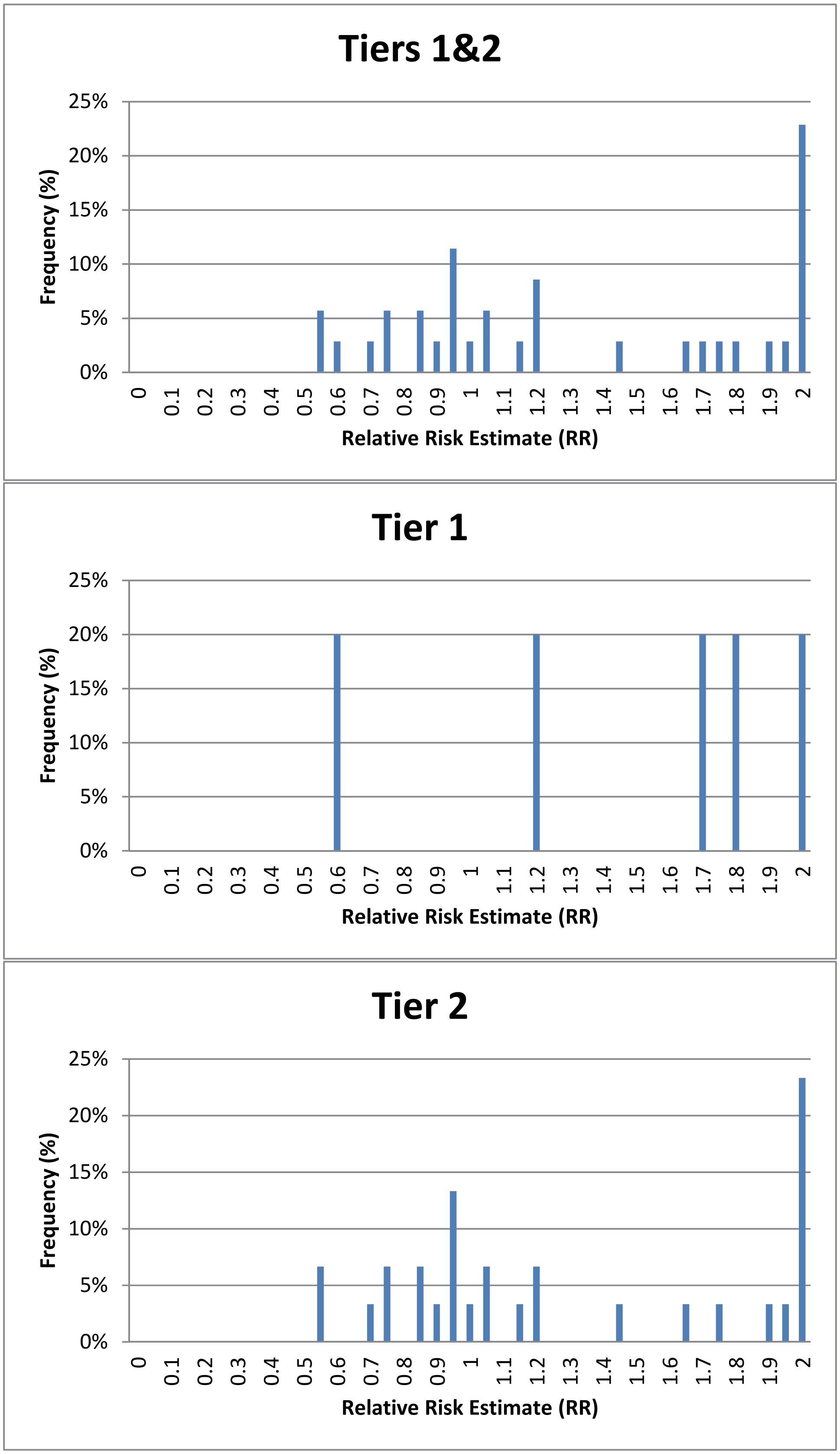

Figure E: Frequency distribution of individual study relative risk estimates: Farming

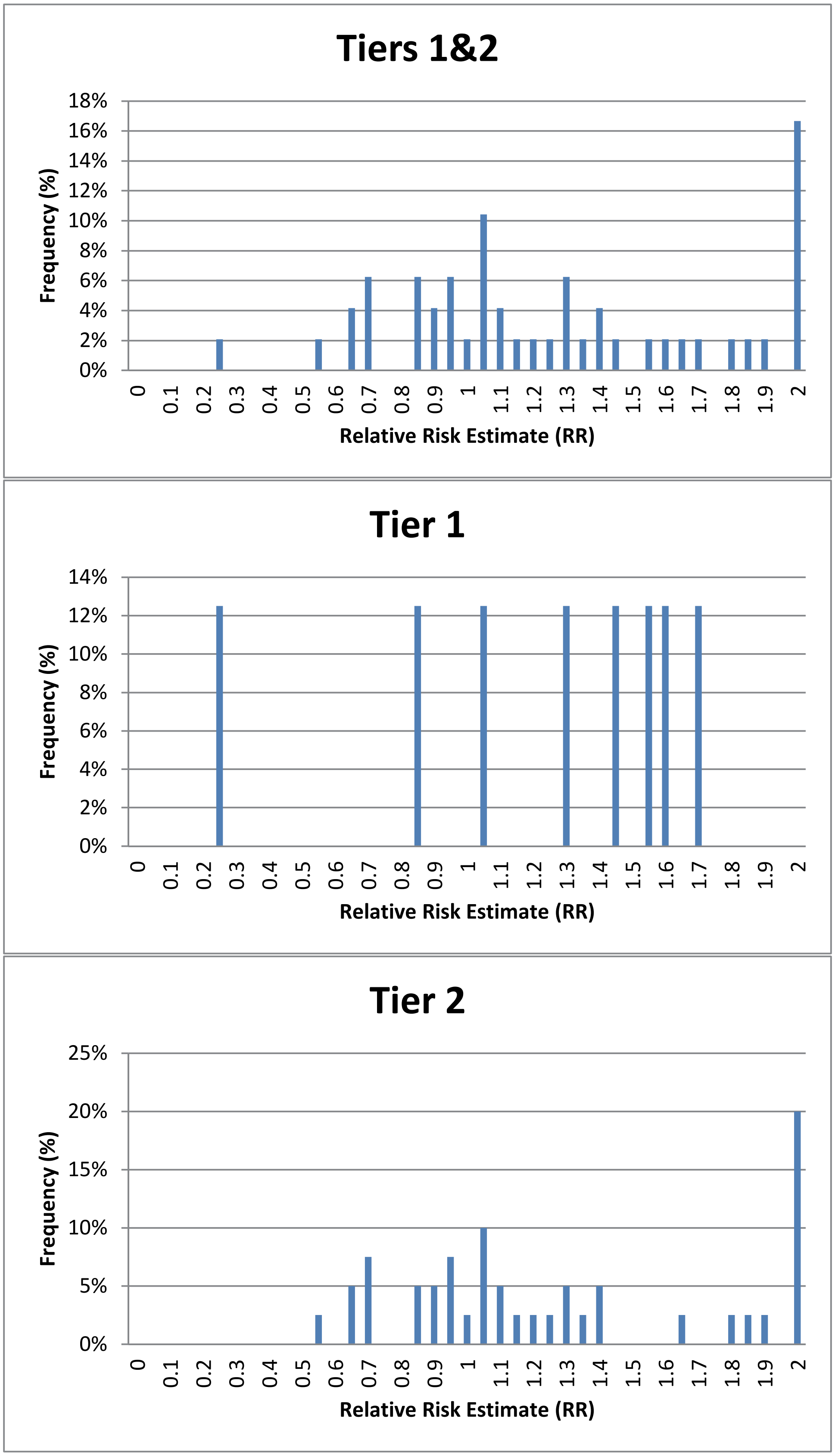

Figure F: Frequency distribution of individual study relative risk estimates: Pesticide use

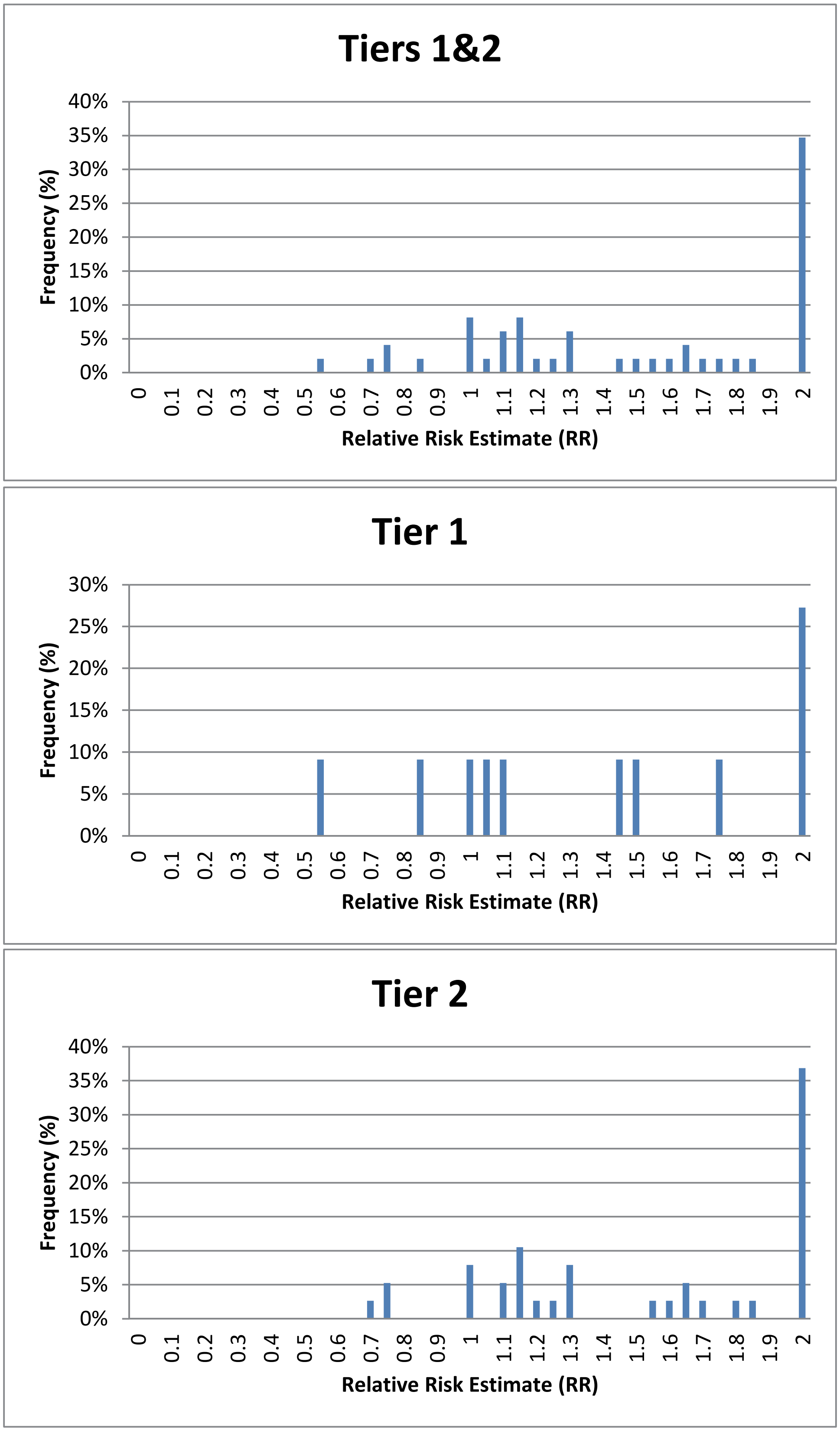

Figure G: Frequency distribution of individual study relative risk estimates: Herbicide use

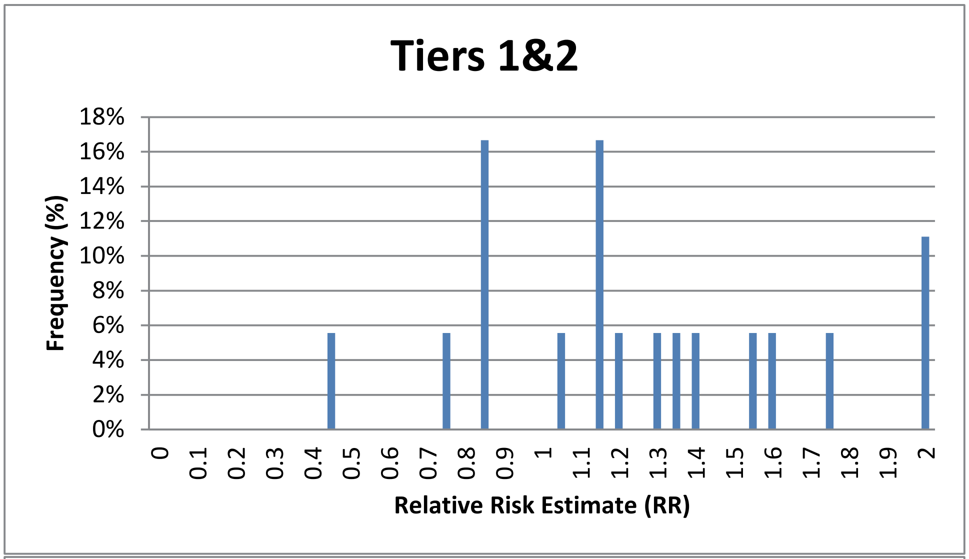

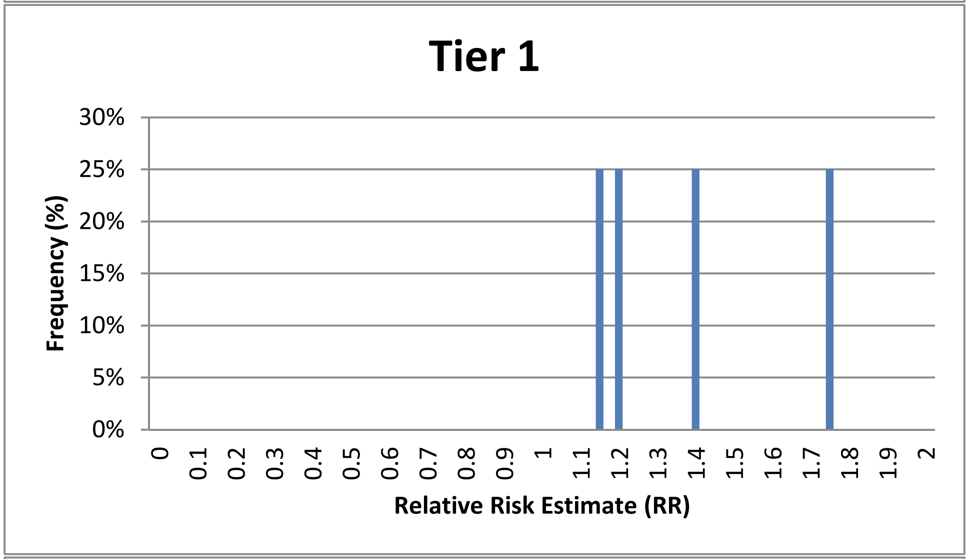

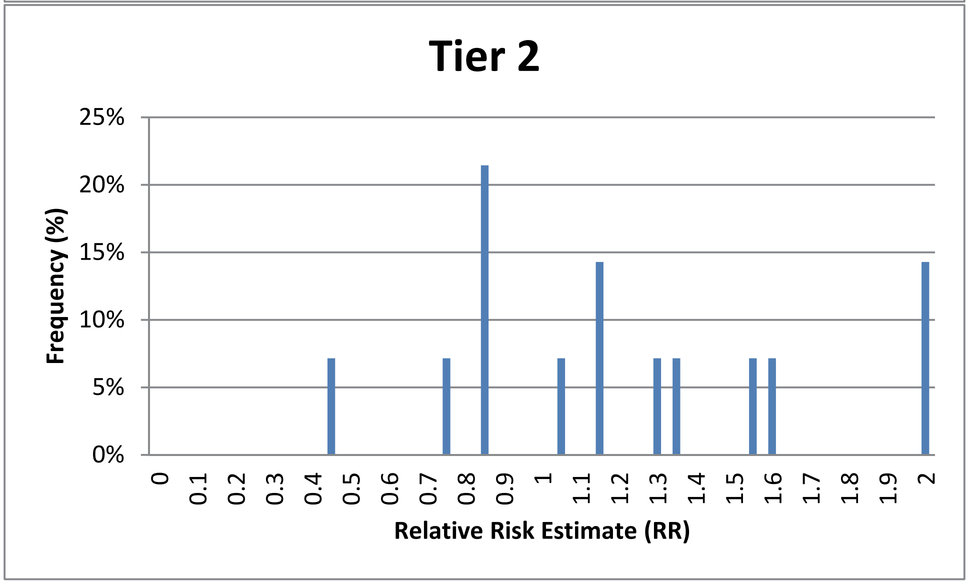

Figure H: Frequency distribution of individual study relative risk estimates: Fungicide use

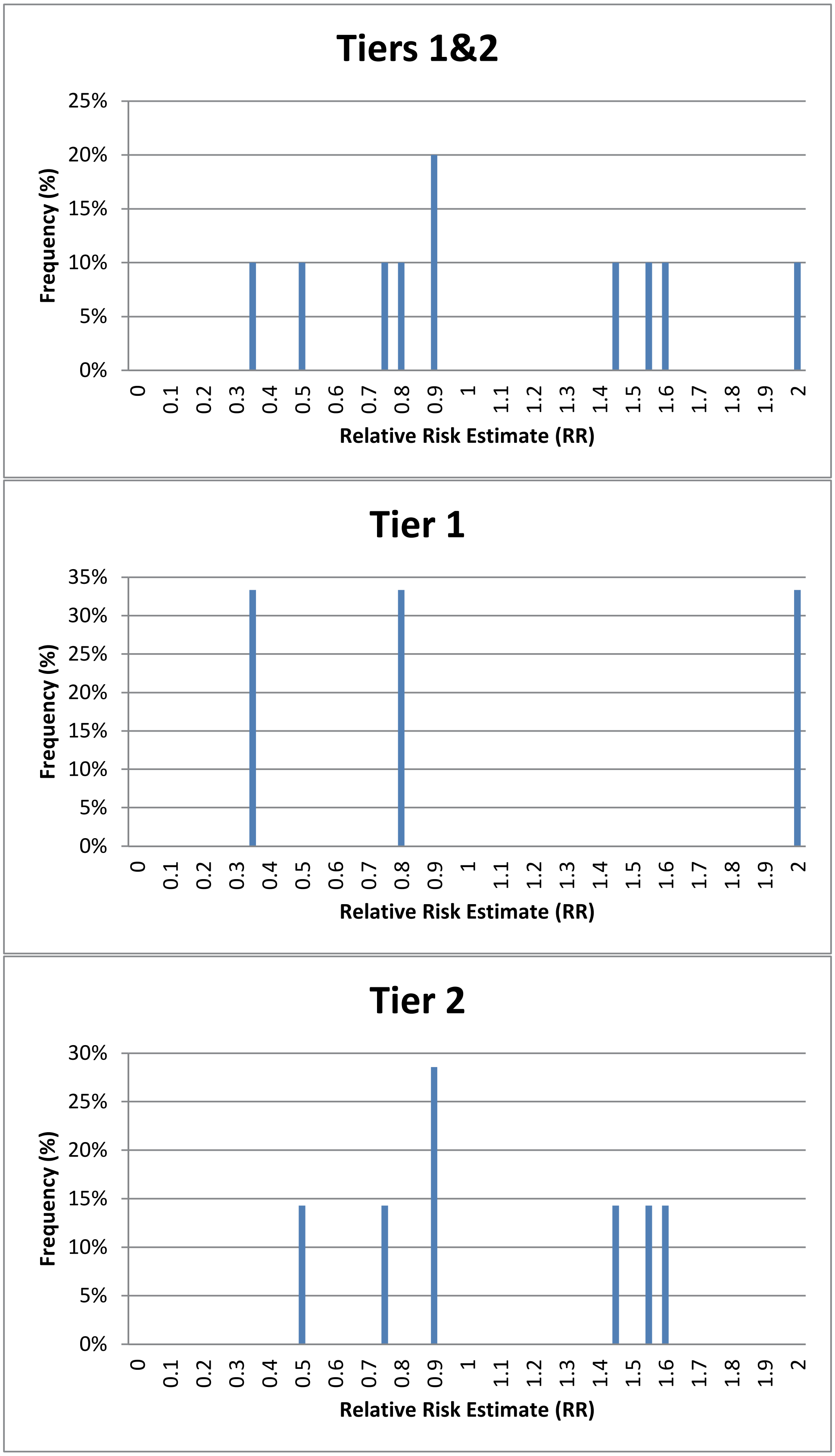

Figure I: Frequency distribution of individual study relative risk estimates: Insecticide use

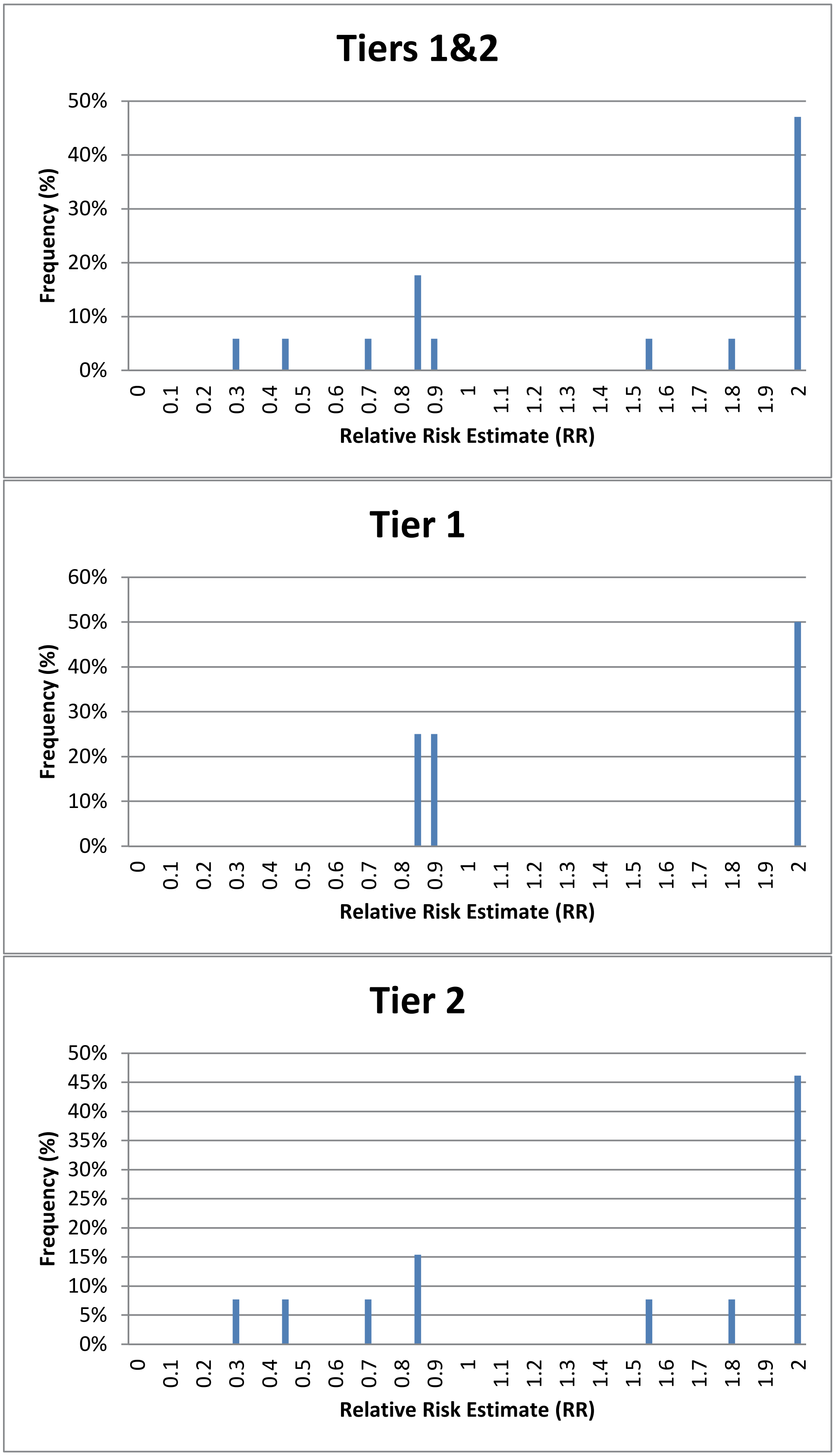

Figure J: Frequency distribution of individual study relative risk estimates: Paraquat use

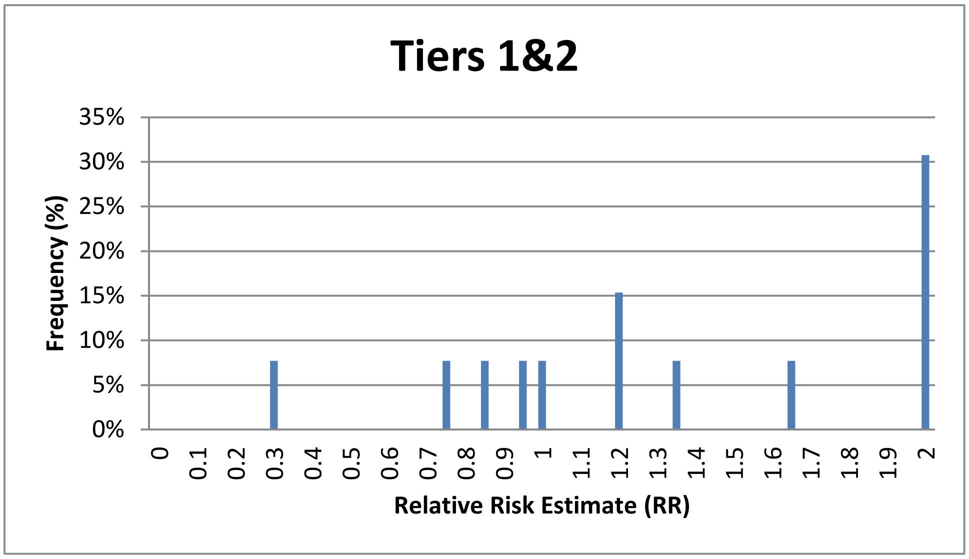

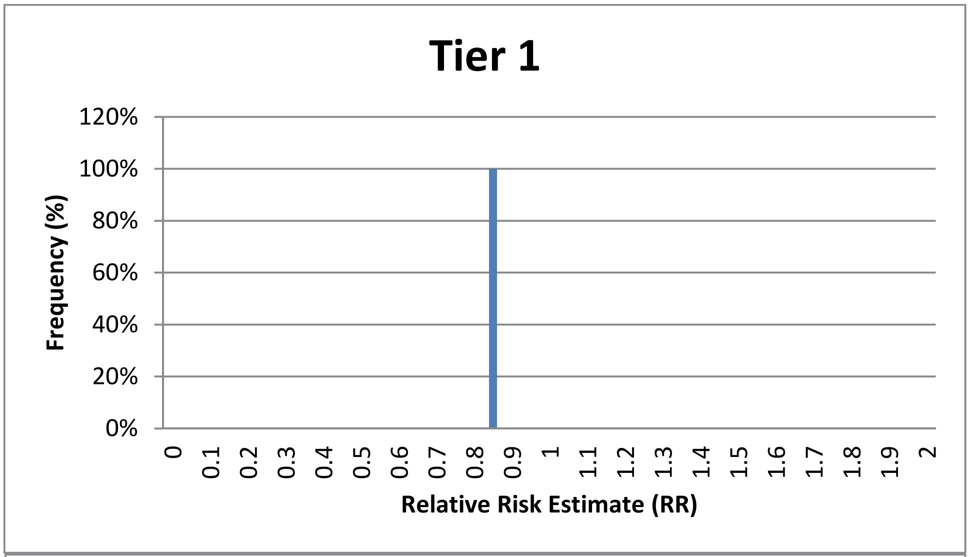

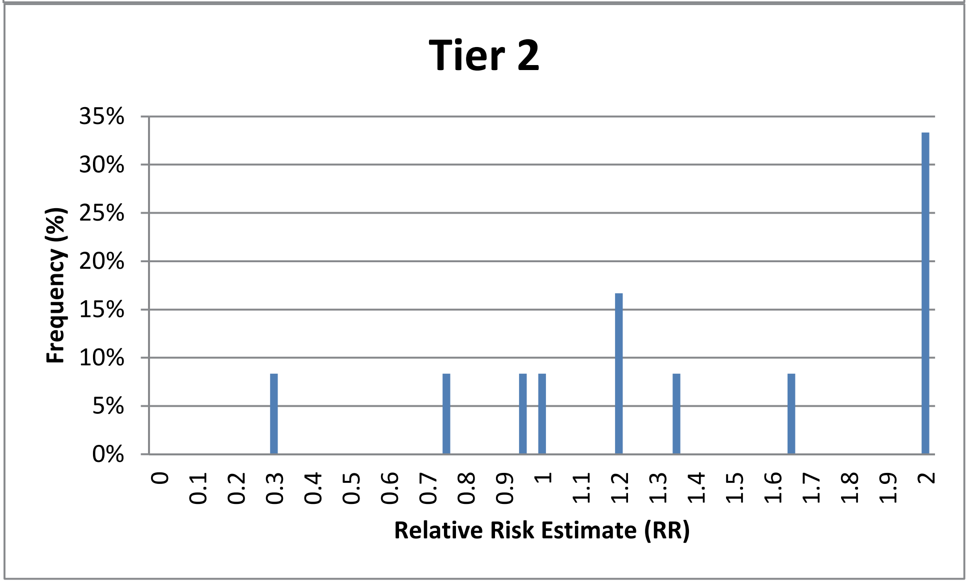

Supplement: S1 File — Figs A to J: Frequency distribution of the relative risk estimates (RRs) from individual epidemiological studies assessing the association between risk factors and Parkinson’s disease. Fig A: Frequency distribution of individual study relative risk estimates: Current cigarette smoking. Fig B: Frequency distribution of individual study relative risk estimates: Heavy or long-term smoking. Fig C: Frequency distribution of individual study relative risk estimates: Rural living. Fig D: Frequency distribution of individual study relative risk estimates: Well-water consumption. Fig E: Frequency distribution of individual study relative risk estimates: Farming. Fig F: Frequency distribution of individual study relative risk estimates: Pesticide use. Fig G: Frequency distribution of individual study relative risk estimates: Herbicide use. Fig H: Frequency distribution of individual study relative risk estimates: Fungicide use. Fig I: Frequency distribution of individual study relative risk estimates: Insecticide use. Fig J: Frequency distribution of individual study relative risk estimates: Paraquat use. (PDF) [file pone.0151841.s003.pdf]
