# Supplementary material for: Association between Parkinson’s Disease and Cigarette Smoking, Rural Living, Well-Water Consumption, Farming and Pesticide Use: Systematic Review and Meta-Analysis
Source: PLoS One. 2016 Apr 7;11(4):e0151841. doi: 10.1371/journal.pone.0151841 (PMC4824443; doi:10.1371/journal.pone.0151841)

Figure A: Sensitivity Analyses – Heavy Smoking

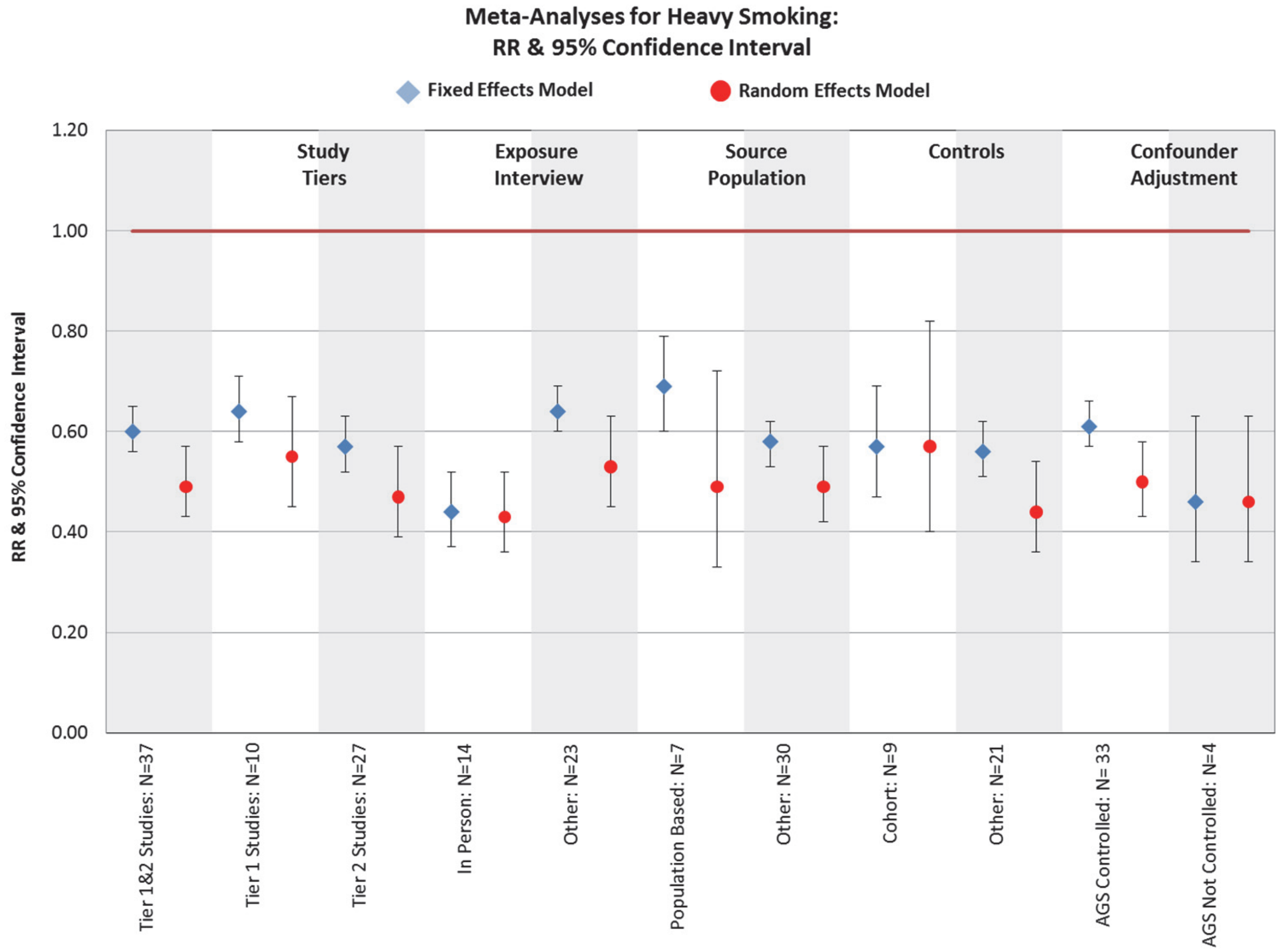

Figure B: Sensitivity Analyses – Rural Living

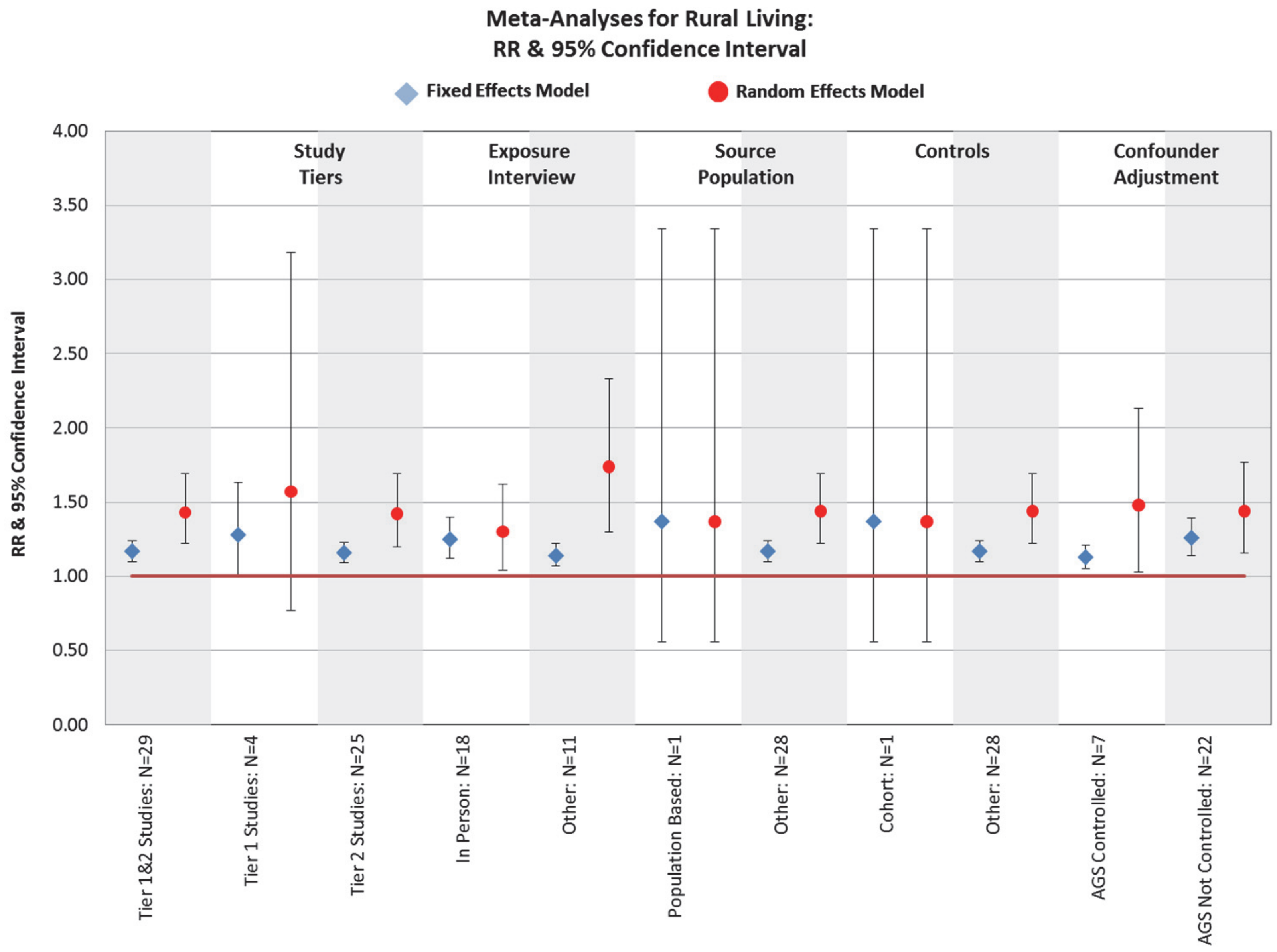

Figure C: Sensitivity Analyses – Well-Water Consumption

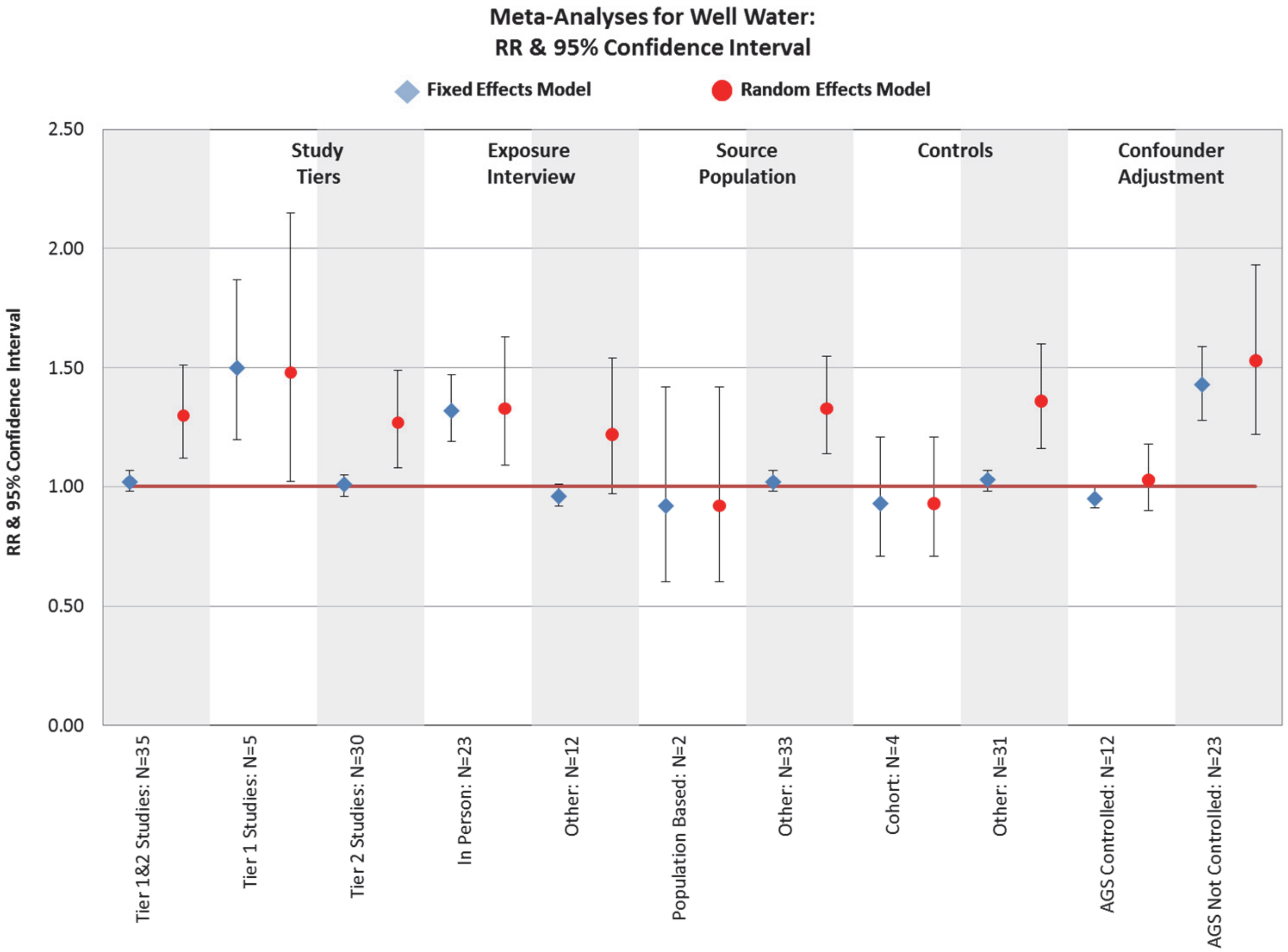

Figure D: Sensitivity Analyses – Farming

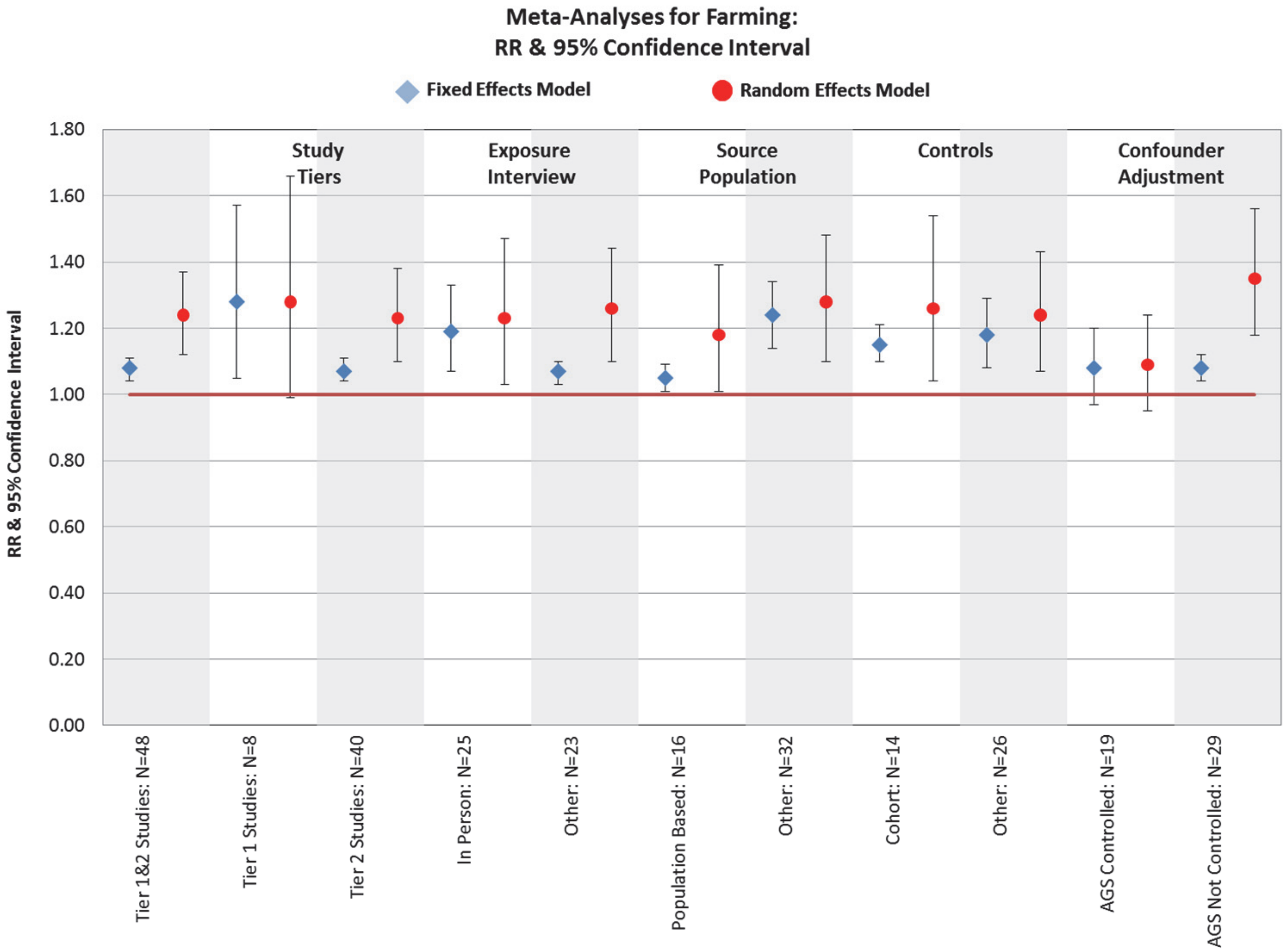

Figure E: Sensitivity Analyses – Pesticide Use

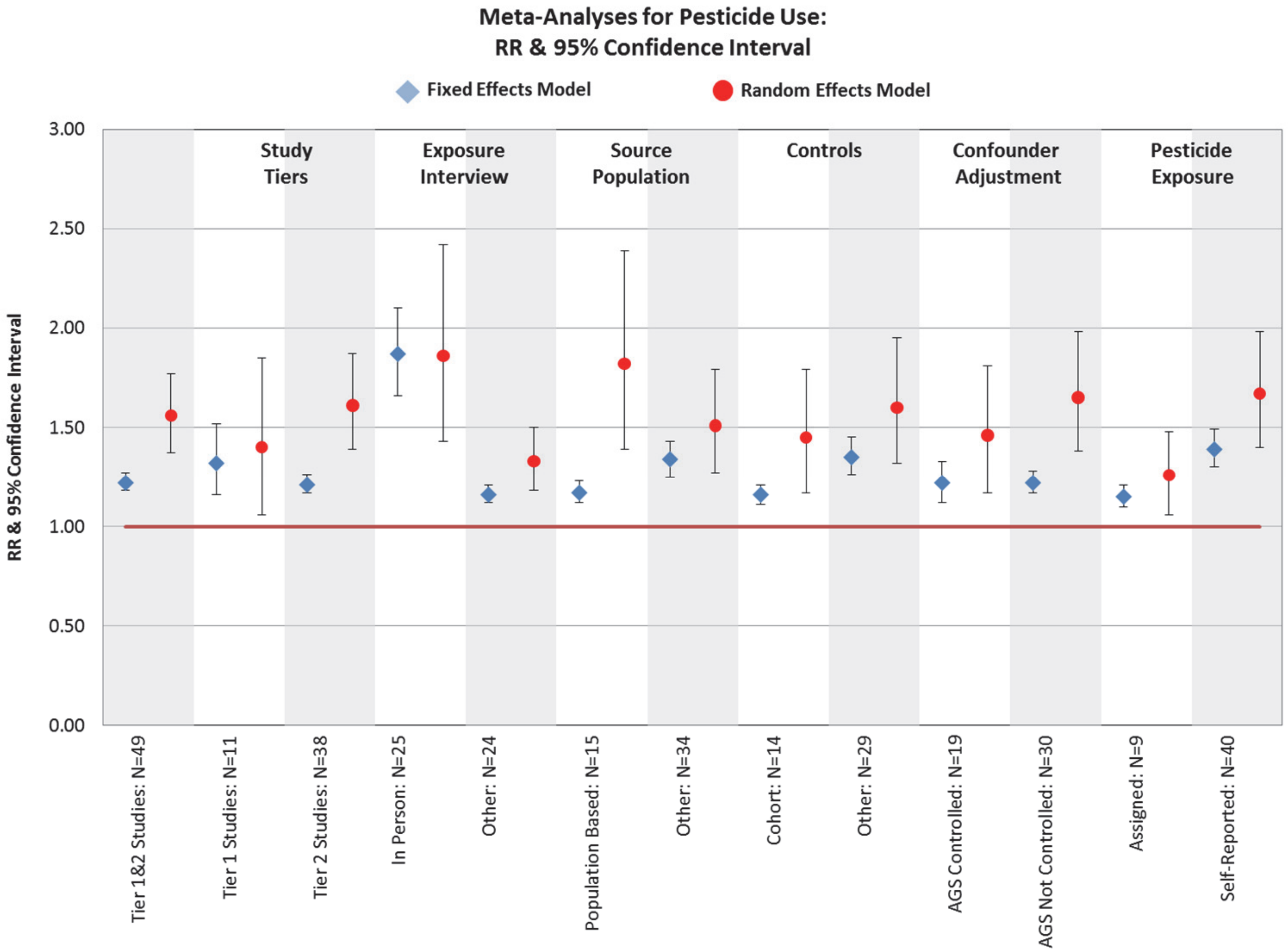

Figure F: Sensitivity Analyses – Herbicide Use

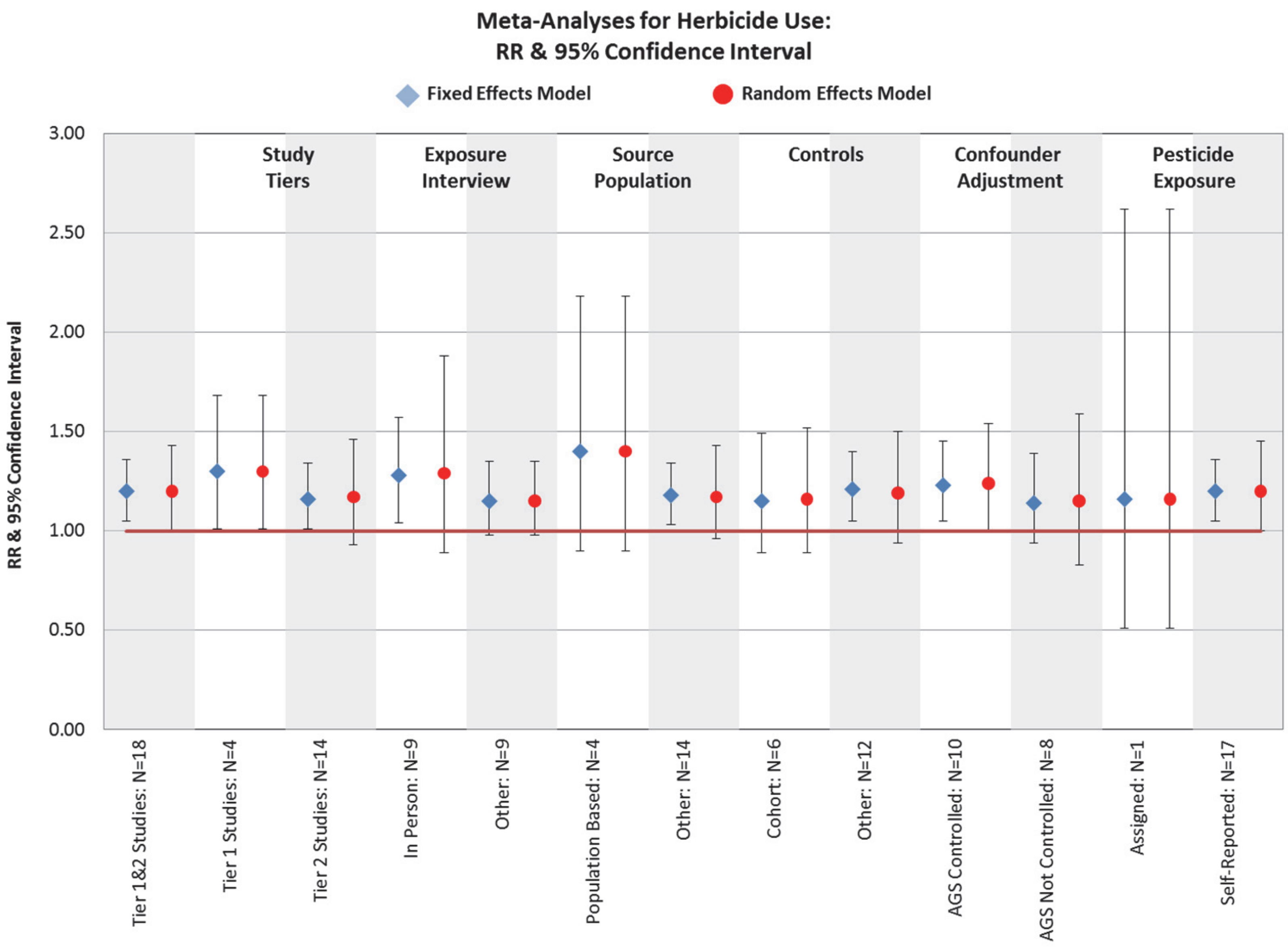

Figure G: Sensitivity Analyses – Fungicide Use

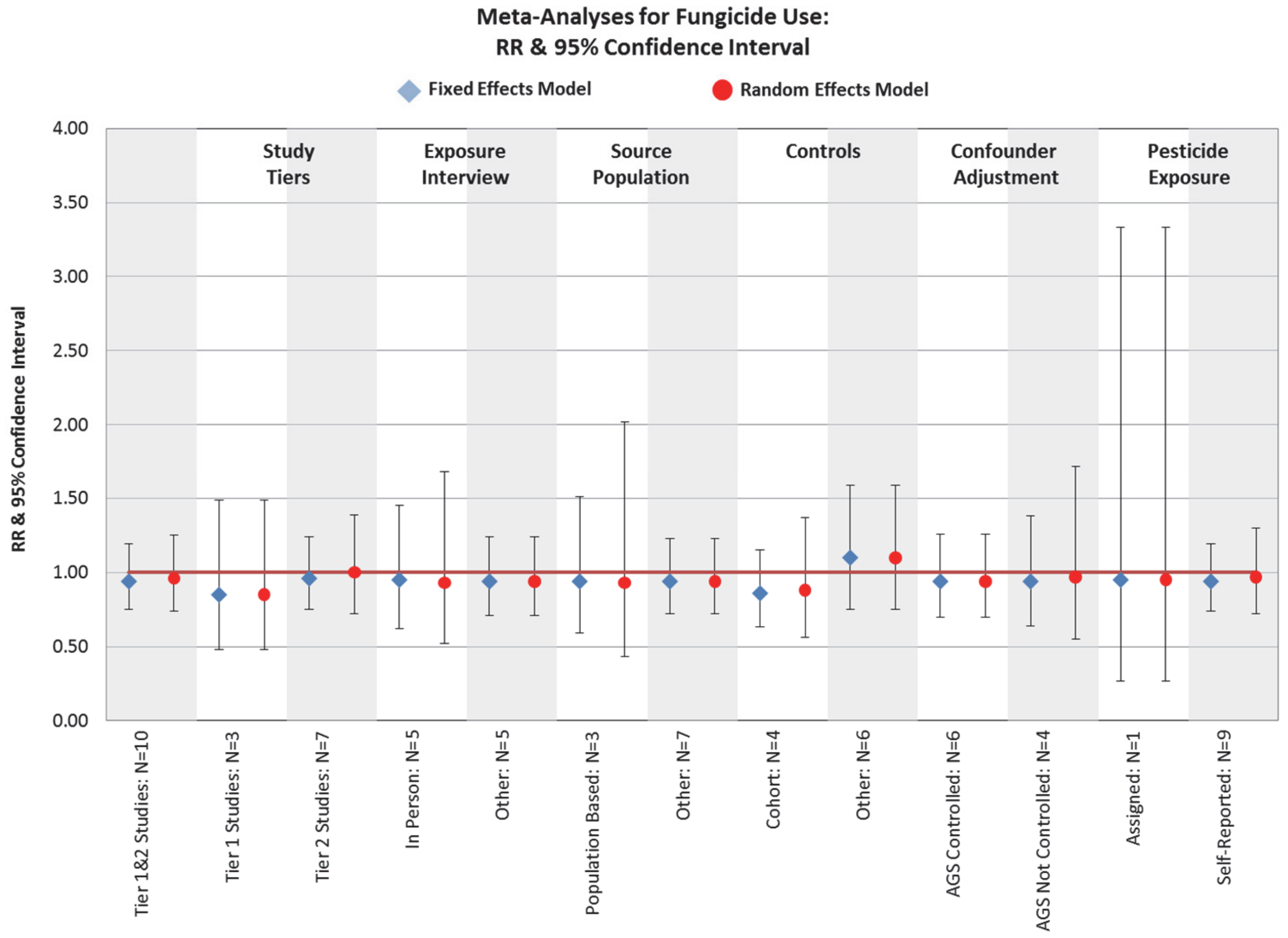

Figure H: Sensitivity Analyses – Insecticide Use

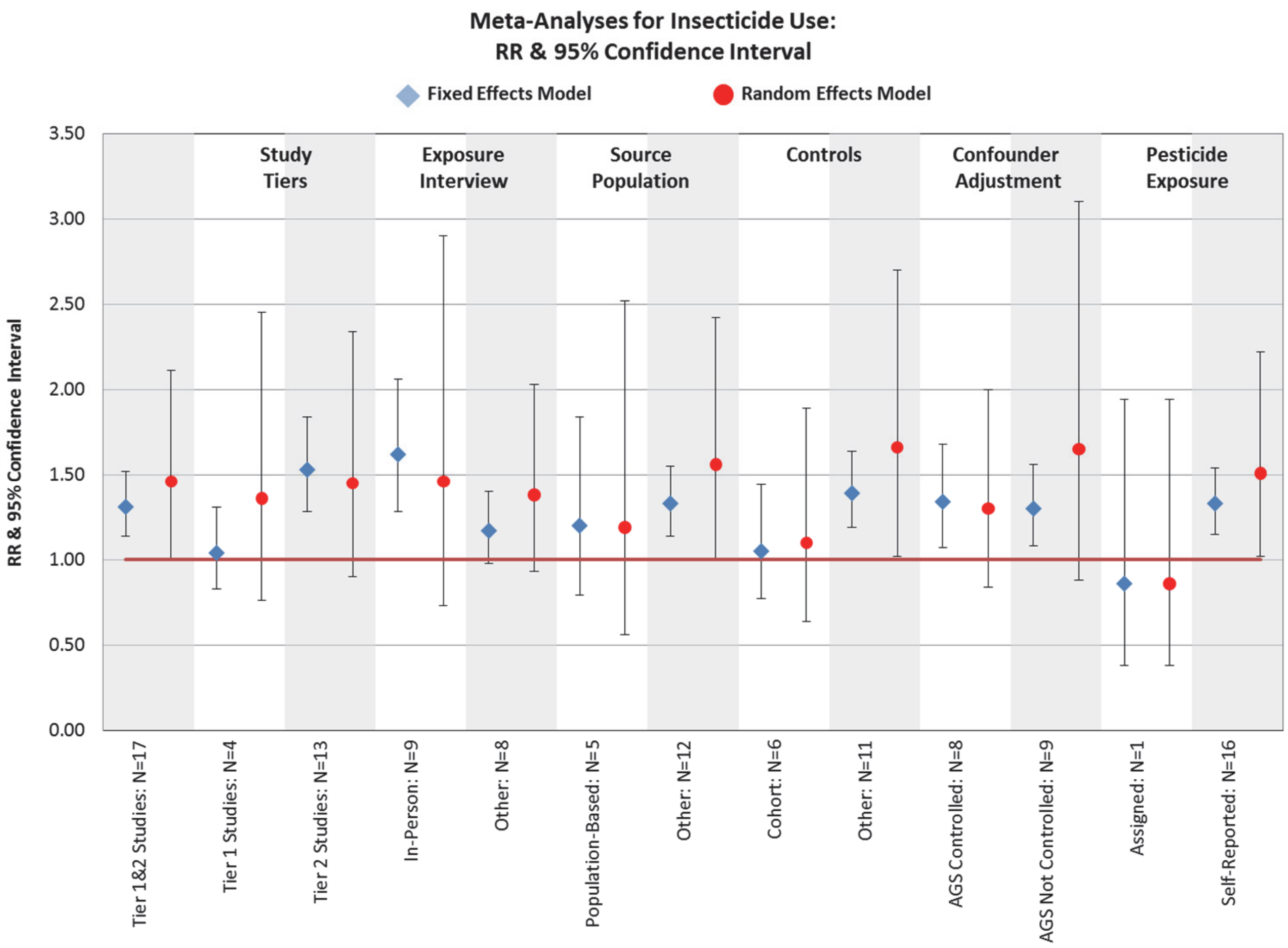

Figure I: Sensitivity Analyses – Paraquat Use

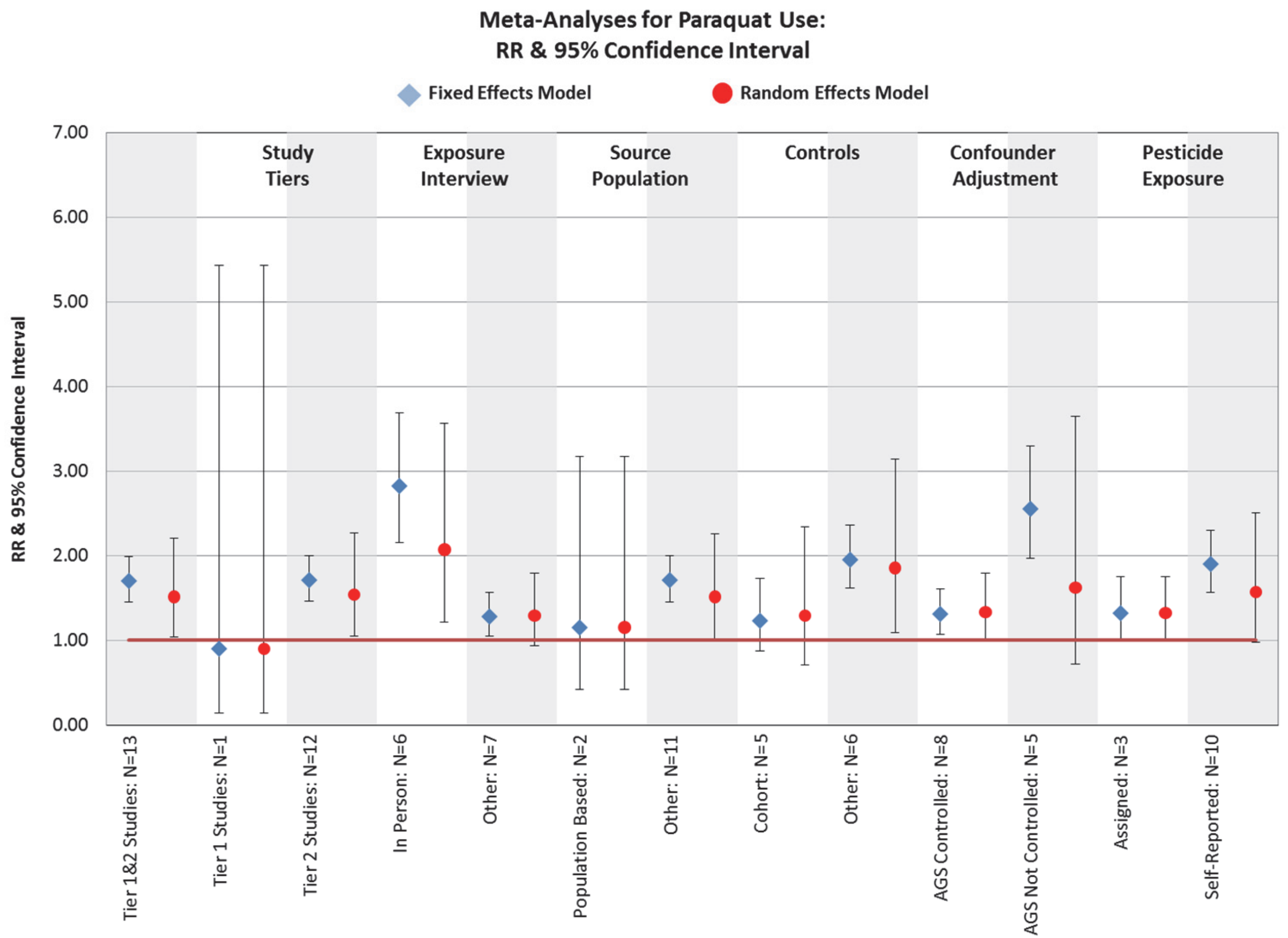

Supplement: S2 File — Figs A to I: Sensitivity analyses of estimated relative risks (RRs) stratified by study characteristics (study quality tier, exposure interview technique, source population, type of controls and extent of confounder adjustment) in fixed and random effects models. Fig A: Sensitivity Analyses–Heavy Smoking. Fig B: Sensitivity Analyses–Rural Living. Fig C: Sensitivity Analyses–Well-Water Consumption. Fig D: Sensitivity Analyses–Farming. Fig E: Sensitivity Analyses–Pesticide Use. Fig F: Sensitivity Analyses–Herbicide Use. Fig G: Sensitivity Analyses–Fungicide Use. Fig H: Sensitivity Analyses–Insecticide Use. Fig I: Sensitivity Analyses–Paraquat Use. (PDF) [file pone.0151841.s004.pdf]
