# Supplementary material for: Association between Parkinson’s Disease and Cigarette Smoking, Rural Living, Well-Water Consumption, Farming and Pesticide Use: Systematic Review and Meta-Analysis
Source: PLoS One. 2016 Apr 7;11(4):e0151841. doi: 10.1371/journal.pone.0151841 (PMC4824443; doi:10.1371/journal.pone.0151841)
Supplement: S3 File — Tables A to I: Metadata characteristics of Tier 1 and Tier 2 studies on the association between risk factors and Parkinson’s disease. Table A: Metadata for Tier 1 and Tier 2 studies: Current cigarette smoking. Table B: Metadata for Tier 1 and Tier 2 studies: Heavy or long-term cigarette smoking. Table C: Metadata for Tier 1 and Tier 2 studies: Rural living. Table D: Metadata for Tier 1 and Tier 2 studies: Well-water consumption. Table E: Metadata for Tier 1 and Tier 2 studies: Farming. Table F: Metadata for Tier 1 and Tier 2 studies: Pesticide use. Table G: Metadata for Tier 1 and Tier 2 studies: Herbicide, fungicide or insecticide use. Table H: Metadata for Tier 1 and Tier 2 studies: High herbicide, fungicide or insecticide use. Table I: Metadata for Tier 1 and Tier 2 studies: Paraquat ever use or high use. (PDF) [file pone.0151841.s005.pdf]

Table A: Metadata for Tier 1 and Tier 2 Studies: Current cigarette smoking

Year	Author	Tier	Case Type	Exposure Type	Diagnostic Data	Reference Group
2000	Benedetti [60]	1	Incident	Individual	Medical records linkage + neurologist chart review	Never smoker
2001	Hernán [61]	1	Incident	Individual	Physician confirmation or chart review	Never smoker, HPFS+NHS
2002	Checkoway [62]	1	Incident	Individual	Neurologist diagnosis or chart review	Never smoker
2005	Park [63]	1	Incident	Individual	Neurology clinic attendance	Non-smoker
2005	Wirdefeldt [64]	1	Incident	Individual	Hospital discharge and cause-of-death registers	Never, M&F, external controls
2007	Thacker [65]	1	Incident	Individual	Physician confirmation or chart review	Never smoker, both sexes
2008	Tan [66]	1	Incident	Individual	Neurologist chart review	Never smoker
2008	Sääksjärvi [67]	1	Incident	Individual	Reimbursement registry + neurologist confirmation	Non-smoker
2009	Costello [68]	1	Incident	Individual	Neurologist examination	Never smoker
2009	Gatto [69]*	1	Incident	Individual	Neurologist examination	Never smoker
2009	Ritz [70]*	1	Incident	Individual	Neurologist examination	Never smoker, both sexes
2010	Chen [71]*	1	Incident	Individual	Neurologist confirmation or chart review	Never smoker, both sexes
2010	Shino [72]	1	Incident	Individual	Neurologist chart review	Never smoker
2011	Feldman [73]*	1	Incident	Individual	Inpatient and death registers	Never smoker, men
2012	Liu [74]	1	Incident	Individual	Physician confirmation or chart review	Never smoker, both sexes
1994	Mayeux [75]	2	Prevalent	Individual	Neurologist examination	Never smoker
1995	Martyn [76]*	2	Prevalent	Individual	Neurologist/geriatrician diagnosis and chart review	Ex-smoker
1995	Martyn [76]	2	Prevalent	Individual	Neurologist/geriatrician diagnosis and chart review	Never smoker
1997	Hellenbrand [77]	2	Prevalent	Individual	Neurologist confirmation	Never smoker, neighbors
1997	Tzourio [78]	2	Prevalent	Individual	Neurologist examination	Never smoker (inferred)
1998	Chan [79]	2	Prevalent	Individual	Neurologist/geriatrician examination	Non-smoker
1999	Fall [80]	2	Prevalent	Individual	Neurologist examination or chart review	Non-current smoker (inferred)
1999	Kuopio [81]	2	Prevalent	Individual	Neurologist examination	Non-current smoker (inferred)
2001	Paganini-Hill [82]	2	Mixed	Individual	Discharge diagnosis, death cert., or self-report	Never smoker
2002	Tsai [83]	2	Prevalent	Individual	Neurologist examination	Never smoker, young-onset PD
2003	Dong [84]	2	Prevalent	Individual	Prevalence survey + 46% neurologist confirmation	Never smoker (Inferred)
2003	Ragonese [85]	2	Prevalent	Individual	Neurology clinic attendance	Never smoker
2004	Ascherio [3]	2	Deceased	Individual	Death certificate	Both sexes
2005	Scott [86]*	2	Prevalent	Individual	Neurologist examination	Never smoker
2005	Galanaud [87]	2	Prevalent	Individual	Neurologist examination or confirmation	Never smoker
2007	Hancock [88]	2	Prevalent	Individual	Neurologist examination	Never, exp. truncated at ref. age
2007	Fong [89]	2	Prevalent	Individual	Neurologist examination	Ever/current vs. never
2007	Kamel [18]	2	Incident	Individual	Self-report	Never, incident PD
2007	Kamel [18]*	2	Prevalent	Individual	Self-report	Never, prevalent PD
2008	Powers [90]	2	Prevalent	Individual	Neurologist diagnosis	Never smoker
2008	Petersen [91]	2	Prevalent	Individual	Neurologist examination	Never smoker

Table A: Metadata for Tier 1 and Tier 2 Studies: Current cigarette smoking (continued)

Year	Author	Tier	Case Type	Exposure Type	Diagnostic Data	Reference Group
2008	Dhillon [92]	2	Prevalent	Individual	Neurologist diagnosis	Non-smoker
2010	Tanaka [93]	2	Prevalent	Individual	Neurologist diagnosis	Never smoker
2010	Kiyohara [94]*	2	Prevalent	Individual	Neurologist diagnosis	Non-smoker
2010	Nicoletti [95]	2	Prevalent	Individual	Neurology clinic attendance	Never smoker
2013	Kyrozis [20]	2	Incident	Individual	Self-report with self-confirmation	Never smoker

*Estimate excluded from meta-analysis due to overlap with another estimate ([69] with [70, 99], [143] with [141], [123] with [129], [127] with [79], [148] with [149], and dual estimates from [18, 131]).

Table B: Metadata for Tier 1 and Tier 2 studies: Heavy or long-term cigarette smoking

Year	Author	Tier	Case Type	Exposure Type	Diagnostic Data	Exposure
1994	Grandinetti [96]	1	Incident	Individual	Neurologist confirmation	Per 10 pack-years
2000	Benedetti [60]	1	Incident	Individual	Medical records linkage + neurologist chart review	> 30 pack-years smoked
2001	Hernán [61]	1	Incident	Individual	Physician confirmation or chart review	15+ cig./day, pooled HPFS & NHS
2002	Checkoway [62]	1	Incident	Individual	Neurologist diagnosis or chart review	40+ pack-years smoked
2005	Wirdefeldt [64]	1	Incident	Individual	Hospital discharge and cause-of-death registers	> 120 cig/week, both sexes, external
2007	Thacker [65]	1	Incident	Individual	Physician confirmation or chart review	45+ pack-yrs vs. never smoker, M&F
2008	Tan [66]	1	Incident	Individual	Neurologist chart review	Current smoker, 13+ cigarettes/day
2009	Costello [68]	1	Incident	Individual	Neurologist examination	> 19 pack-yrs of cig. smoking
2009	Ritz [70]*	1	Incident	Individual	Neurologist examination	40+ pack-years of smoking
2010	Chen [71]	1	Incident	Individual	Neurologist confirmation or chart review	50+ pack-years of smoking, M&F
2012	Liu [74]	1	Incident	Individual	Physician confirmation or chart review	≥ 30 yrs, past smokers
1990	Sasco [97]	2	Prev./dead	Individual	Self-report or death cert., 61% physician confirm.	Current smoking ≥ 40 yrs
1990	Sasco [97]*	2	Prev./dead	Individual	Self-report or death cert., 61% physician confirm.	40+ cigarettes/day
1993	Butterfield [98]	2	Prevalent	Individual	Neurologist confirmation	Packs/day 15 yrs before, model 2
1993	Wang [99]	2	Prevalent	Individual	Neurologist examination	Smoking > 20/day
1994	Mayeux [75]	2	Prevalent	Individual	Neurologist examination	> 30 pack-years
1994	Morano [100]	2	Prevalent	Individual	Neurology clinic attendance	> 20 cigarettes/day, males
1997	Hellenbrand [77]	2	Prevalent	Individual	Neurologist confirmation	> 40 pack-years vs. neighbors
1997	Liou [59]	2	Prevalent	Individual	Neurological examination	20+ yrs of smoking cigarettes
1999	Gorell [101]*	2	Prevalent	Individual	Neurologist diagnosis or examination	> 30 pack-years, current smoker
1999	Fall [80]	2	Prevalent	Individual	Neurologist examination or chart review	24-123 pack-years cigarettes
1999	Taylor [102]	2	Prevalent	Individual	Neurologist examination	Per 10 pack-years
2000	Vanacore [103]	2	Prevalent	Individual	Neurologist chart review	> 30 pack-years
2001	Paganini-Hill [82]	2	Mixed	Individual	Discharge diagnosis, death cert., or self-report	Current smoking, 1+ pack/day
2001	Behari [104]	2	Prevalent	Individual	Neurological examination	Smoking > 20 years
2003	Dong [84]	2	Prevalent	Individual	Prevalence survey + 46% neurologist confirmation	20+ years smoked
2003	Tan [105]	2	Prevalent	Individual	Neurologist diagnosis	Smoking 3 packs/day for 10 years
2003	Pals [106]	2	Prevalent	Individual	Neurologist examination	Lifetime max daily # cig. (per 20 cig.)
2003	Ragonese [85]	2	Prevalent	Individual	Neurology clinic attendance	> 30 pack-years
2003	Baldereschi [107]	2	Prevalent	Individual	Neurological examination	20+ pack-years
2004	Gorell [108]	2	Prevalent	Individual	Neurologist diagnosis or examination	> 30 pack-years
2005	Galanaud [87]*	2	Prevalent	Individual	Neurologist examination or confirmation	> 17.4 pack-years
2005	Scott [86]*	2	Prevalent	Individual	Neurologist examination	> 18.75 pack-yrs, truncated at ref.
2006	Evans [109]	2	Prevalent	Individual	Neurology clinic attendance	40.1-50 pack-years of cig. smoking
2006	Ma [110]	2	Prevalent	Individual	Neurological examination	> 30 pack-years

Table B: Metadata for Tier 1 and Tier 2 studies: Heavy or long-term cigarette smoking (continued)

Year	Author	Tier	Case Type	Exposure Type	Diagnostic Data	Exposure
2007	Hancock [88]	2	Prevalent	Individual	Neurologist examination	> 48 pack-yrs, truncated at ref. age
2007	Ritz [111]*	2	Prev./incid.	Individual	Various (pooled analysis)	60+ pack-years, both sexes
2007	Kamel [18]	2	Incident	Individual	Self-report	> 30 pack-yrs, incident PD
2007	Kamel [18]*	2	Prevalent	Individual	Self-report	> 30 pack-yrs, prevalent PD
2008	Powers [90]	2	Prevalent	Individual	Neurologist diagnosis	40+ pack-years
2008	Petersen [91]	2	Prevalent	Individual	Neurologist examination	Smoking for 30 yrs or more
2009	Elbaz [55]	2	Prevalent	Individual	Neurologist examination or confirmation	Ever cig. smoke, pack-yrs > 17
2010	Tanaka [93]	2	Prevalent	Individual	Neurologist diagnosis	30+ pack-years of smoking
2010	Nicoletti [95]	2	Prevalent	Individual	Neurology clinic attendance	35+ years of smoking

*Estimate excluded from meta-analysis due to overlap with another estimate ([55] with [142], [143] with [141], [162] with [156], [69] with [99], [69, 78, 120, 121, 130, 151, 156] with [112], and dual estimates from [18, 152]).

Table C: Metadata for Tier 1 and Tier 2 studies: Rural living

Year	Author	Tier	Case Type	Exposure Type	Diagnostic Data	Exposure
2005	Wirdefeldt [64]	1	Incident	Individual	Hospital discharge and cause-of-death registers	Rural area, both sexes, external
2005	Firestone [114]	1	Incident	Individual	Neurologist chart review	Home based - agriculture region
2010	Vlajinac [115]	1	Incident	Individual	Neurologist diagnosis	Rural living any time
1989	Ho [116]	2	Prevalent	Individual	Neurological examination and confirmation	Rural living > 40 yrs
1989	Tanner [117]	2	Prevalent	Individual	Neurologist examination	Village residence
1990	Koller [118]	2	Prevalent	Individual	Neurologist examination	Rural residence
1991	Stern [119]	2	Prevalent	Individual	Neurology clinic attendance and chart review	Rural living ≥ 1 year vs. never
1992	Jiménez-J. [120]	2	Prevalent	Individual	Neurology clinic attendance	Ever lived in town with pop. < 2,000
1993	Butterfield [98]	2	Prevalent	Individual	Neurologist confirmation	Rural residence at diagnosis, model 3
1993	Wang [99]	2	Prevalent	Individual	Neurologist examination	Rural areas
1993	Hubble [54]†	2	Prevalent	Individual	Neurologist examination	Urban Study, currently rural residence
1993	Hubble [54]†	2	Prevalent	Individual	Neurologist examination	Rural Study, currently rural residence
1994	Morano [100]	2	Prevalent	Individual	Neurology clinic attendance	Rural living (towns with pop. < 2,000)
1995	Martyn [76]	2	Prevalent	Individual	Neurologist/geriatrician diagnosis and chart review	First home in village vs. large town
1996	Seidler [121]	2	Prevalent	Individual	Neurologist confirmation	Low avg. pop. density, neighbors
1997	Liou [59]	2	Prevalent	Individual	Neurological examination	Living in rural residence
1998	Gorell [122]	2	Prevalent	Individual	Neurologist diagnosis or examination	Lived in rural area
1998	De Palma [123]	2	Prevalent	Individual	Neurology clinic attendance	Living in a rural area ≥ 10 yrs
1998	Marder [124]	2	Prevalent	Individual	Neurologist examination	Rural living
1998	McCann [125]	2	Prevalent	Individual	"All participants were reviewed"	Rural residency
1999	Taylor [102]	2	Prevalent	Individual	Neurologist examination	Years of rural living
1999	Werneck [126]	2	Prevalent	Individual	Neurologist examination	Rural life
2000	Preux [127]	2	Prevalent	Individual	Neurologist examination	Non-urban area (≤ 2,000 pop.)
2001	Behari [104]	2	Prevalent	Individual	Neurological examination	Rural residency > 10 yrs
2002	Zorzon [128]	2	Prevalent	Individual	Neurological examination	Rural living
2003	Baldi [149]	2	Prevalent	Ecologic	Neurologist referral	Rural residency, overall
2005	Wright [129]	2	Prevalent	Individual	Support group or exercise group attendance	Lived in town (< 1,500 pop.) 1st 40 yrs
2010	Sanyal [130]	2	Prevalent	Individual	Neurologist examination	Rural living
2011	Das [58]	2	Prevalent	Individual	Neurologist confirmation	Rural living place

†Independent stratum-specific estimates obtained from the same study and used in the meta-analysis.

Table D: Metadata for Tier 1 and Tier 2 studies: Well-water consumption

Year	Author	Tier	Case Type	Exposure Type	Diagnostic Data	Water Source
2004	Park [131]	1	Incident	Individual	Neurology clinic attendance	Well, healthy controls
2004	Park [131]*	1	Incident	Individual	Neurology clinic attendance	Well, neurological controls
2005	Firestone [114]	1	Incident	Individual	Neurologist chart review	Well, lifelong
2005	Park [63]	1	Incident	Individual	Neurology clinic attendance	Well, crude, neurological controls
2009	Gatto [69]	1	Incident	Individual	Neurologist examination	Well, ever or lifetime
2010	Vlajinac [115]	1	Incident	Individual	Neurologist diagnosis	Well, ever
1989	Tanner [117]	2	Prevalent	Individual	Neurologist examination	Well
1990	Koller [118]	2	Prevalent	Individual	Neurologist examination	Well
1991	Stern [119]	2	Prevalent	Individual	Neurology clinic attendance and chart review	Well ≥ 1 year vs. never
1992	Jiménez-J. [120]	2	Prevalent	Individual	Neurology clinic attendance	Well (≥ 1 yr)
1993	Wang [99]	2	Prevalent	Individual	Neurologist examination	Well
1994	Hertzman [132]	2	Prevalent	Individual	Neurologist examination	Well, both sexes
1994	Morano [100]	2	Prevalent	Individual	Neurology clinic attendance	Well, ≥ 1 yr
1996	De Michele [133]	2	Prevalent	Individual	Neurology clinic attendance	Well
1996	Seidler [121]	2	Prevalent	Individual	Neurologist confirmation	Well, neighbors
1997	Liou [59]	2	Prevalent	Individual	Neurological examination	Well
1998	McCann [125]	2	Prevalent	Individual	"All participants were reviewed"	Well, bore, or spring water
1998	Gorell [122]	2	Prevalent	Individual	Neurologist diagnosis or examination	Well, ever
1998	Chan [79]	2	Prevalent	Individual	Neurologist/geriatrician examination	Well
1998	Marder [124]	2	Prevalent	Individual	Neurologist examination	Unfiltered water
1998	De Palma [123]*	2	Prevalent	Individual	Neurology clinic attendance	Well ≥ 10 yrs
1998	Smargiassi [134]	2	Prevalent	Individual	Neurology clinic attendance	Well ≥ 10 yrs
1999	Taylor [102]	2	Prevalent	Individual	Neurologist examination	Well; years of consumption
1999	Kuopio [81]	2	Prevalent	Individual	Neurologist examination	Pooled, calculated with comp. meta
1999	Werneck [126]	2	Prevalent	Individual	Neurologist examination	Well
2000	Preux [127]	2	Prevalent	Individual	Neurologist examination	Well only
2001	Behari [104]	2	Prevalent	Individual	Neurological examination	Well-water drinking > 10 yrs
2001	Engel [135]	2	Prevalent	Individual	Neurological examination	Well
2002	Zorzon [128]	2	Prevalent	Individual	Neurological examination	Well
2002	Tsai [83]	2	Prevalent	Individual	Neurologist examination	Well, young-onset PD
2003	Dong [84]	2	Prevalent	Individual	Prevalence survey + 46% neurologist confirmation	Well
2005	Wright [129]	2	Prevalent	Individual	Support group or exercise group attendance	Well, any childhood exposure
2007	Dick [136]	2	Prevalent	Individual	Neurologist confirmation or chart review	River or well
2008	Hancock [137]	2	Prevalent	Individual	Neurologist examination	Well, child or adult
2009	Elbaz [55]	2	Prevalent	Individual	Neurologist examination or confirmation	Well, ever
2010	Sanyal [130]	2	Prevalent	Individual	Neurologist examination	Well

Table D: Metadata for Tier 1 and Tier 2 studies: Well-water consumption (continued)

Year	Author	Tier	Case Type	Exposure Type	Diagnostic Data	Water Source
2011	Das [58]	2	Prevalent	Individual	Neurologist confirmation	Well

*Estimate excluded from meta-analysis due to overlap with another estimate ([184] and [172] and dual estimates from [181]).

Table E: Metadata for Tier 1 and Tier 2 studies: Farming

Year	Author	Tier	Case Type	Exposure Type	Diagnostic Data	Exposure
2004	Park [131]*	1	Incident	Individual	Neurology clinic attendance	Agr production crops, healthy ctrl.
2004	Park [131]	1	Incident	Individual	Neurology clinic attendance	Occupation - farmers, healthy ctrl.
2005	Frigerio [138]*	1	Incident	Individual	Medical records linkage + neurologist chart review	Agricul., forest, fish - med. records
2005	Frigerio [138]	1	Incident	Individual	Medical records linkage + neurologist chart review	Agricul., forestry, fishing - tel. data
2005	Park [63]*	1	Incident	Individual	Neurology clinic attendance	Agr production crops, neurol. ctrl.
2005	Park [63]	1	Incident	Individual	Neurology clinic attendance	Occupation - farmers, neurol. ctrl.
2005	Firestone [114]*	1	Incident	Individual	Neurologist chart review	Occup. - crop farming, men
2006	Ascherio [139]	1	Incident	Individual	Physician confirmation or chart review	Exposed to pesticide, farmer
2010	Skeie [140]	1	Incident	Individual	Neurological examination	Agricultural workers
2010	Firestone [57]	1	Incident	Individual	Neurologist chart review	Farming and related, M&F, weighted
2010	Vlajinac [115]	1	Incident	Individual	Neurologist diagnosis	Agricultural worker
2011	Feldman [73]	1	Incident	Individual	Hospital discharge and cause-of-death registers	Animal handling, men
1989	Ho [116]	2	Prevalent	Individual	Neurological examination and confirmation	Farming > 20 yrs
1990	Hertzman [141]	2	Prevalent	Individual	Neurologist examination	Worked in orchard
1990	Koller [118]	2	Prevalent	Individual	Neurologist examination	Farming
1990	Tanner [142]	2	Prevalent	Individual	Neurology clinic attendance or self-report	Direct exposure to farming
1992	Semchuk [143]	2	Prevalent	Individual	Neurologist confirmation	Agricultural work
1994	Hertzman [132]	2	Prevalent	Individual	Neurologist examination	Farming, both sexes
1995	Chaturvedi [19]	2	Prevalent	Individual	Self-report	Gardening
1996	Rocca [144]	2	Prevalent	Individual	Neurologist examination and confirmation	Farmers
1996	Seidler [121]	2	Prevalent	Individual	Neurologist confirmation	Farming or agr. employment,
1997	Liou [59]	2	Prevalent	Individual	Neurological examination	Farming
1998	Gorell [122]	2	Prevalent	Individual	Neurologist diagnosis or examination	Lived or worked on a farm
1998	Smargiassi [134]	2	Prevalent	Individual	Neurology clinic attendance	Farming, ≥ 10 consecutive years
1998	Marder [124]*	2	Prevalent	Individual	Neurologist examination	Farms in the area
1998	Marder [124]	2	Prevalent	Individual	Neurologist examination	Gardening
1998	Chan [79]	2	Prevalent	Individual	Neurologist/geriatrician examination	Farming
1999	Tsui [145]	2	Prevalent	Individual	Neurologist diagnosis or confirmation	Farming/horticulture
1999	Kuopio [81]	2	Prevalent	Individual	Neurologist examination	Both occup. & part-time farming
1999	Fall [80]	2	Prevalent	Individual	Neurologist examination or chart review	Agriculture, men
2000	Preux [127]	2	Prevalent	Individual	Neurologist examination	Living on a farm for > 1 yr
2000	Tuchsen [146]	2	Prevalent	Individual	Hospital inpatient register	All M & F in agriculture & horticulture
2001	Behari [104]	2	Prevalent	Individual	Neurological examination	Farming > 10 yrs
2001	Engel [135]	2	Prevalent	Individual	Neurological examination	Exposure - farm employment
2001	Kirkey [147]*	2	Prevalent	Individual	Neurologist diagnosis or examination	Agriculture, fishery, forestry
2002	Lee [148]	2	Deceased	Individual	Death certificate	Prop. mortality, crop farmers, total

Table E: Metadata for Tier 1 and Tier 2 studies: Farming (continued)

Year	Author	Tier	Case	Exposure Type	Diagnostic Data	Exposure
2002	Zorzon [128]	2	Prevalent	Individual	Neurological examination	Farming as an occupation
2003	Baldereschi [107]	2	Prevalent	Individual	Neurological examination	Farming for at least 10 years
2003	Baldi [149]	2	Prevalent	Ecologic	Neurologist referral	Lived in vineyard district, M & F
2003	Dong [84]	2	Prevalent	Individual	Prevalence survey + 46% neurologist confirmation	Farming experience
2003	Duzcan [150]	2	Prevalent	Individual	Neurologist examination and confirmation	Farming
2005	Park [151]	2	Deceased	Individual	Death certificate	All farm-related occupations
2005	Wright [129]	2	Prevalent	Individual	Support group or exercise group attendance	Worked on a farm
2005	Galanaud [87]*	2	Prevalent	Individual	Neurologist examination or confirmation	Farmer or farm worker
2005	Goldman [152]	2	Prevalent	Individual	Neurologist examination	Farming, observed vs. expected
2007	Dick [153]	2	Prevalent	Individual	Neurologist confirmation or chart review	Agriculture, fishery, forestry, & related
2008	Hancock [137]	2	Prevalent	Individual	Neurologist examination	History of farming, ever
2008	Dhillon [92]	2	Prevalent	Individual	Neurologist diagnosis	Ever worked in or around farm
2009	Tanner [154]	2	Prevalent	Individual	Neurologist diagnosis	Occupation - farm, fish, & forestry
2009	Elbaz [55]	2	Prevalent	Individual	Neurologist examination or confirmation	Farming, ever
2010	Sanyal [130]	2	Prevalent	Individual	Neurologist examination	Occupation, farmer
2011	Das [58]	2	Prevalent	Individual	Neurologist confirmation	Farming as an occupation
2011	Tanaka [155]	2	Prevalent	Individual	Neurologist diagnosis	Farm, fish, forestry (occup. group)
2011	Rugbjerg [156]	2	Prevalent	Individual	Neurological examination and neurologist review	Agricultural jobs
2013	Kyrozis [20]	2	Incident	Individual	Self-report with self-confirmation	Farming

*Estimate excluded from meta-analysis due to overlap with another estimate ([171] with [197], [55] with [142], [57] with [73], and dual estimates from [122, 173, 181, 188]).

Table F: Metadata for Tier 1 and Tier 2 studies: Pesticide use

Year	Author	Tier	Case Type	Exposure Type	Diagnostic Data	Exposure
2003	Baldi [56]†	1	Incident	Individual	Neurological examination	Occupational, men
2003	Baldi [56]†	1	Incident	Individual	Neurological examination	Occupational, women
2005	Firestone [114]*	1	Incident	Individual	Neurologist chart review	Occupational, men
2006	Ascherio [139]	1	Deceased	Individual	Death certificate	Current or regular past use
2006	Frigerio [157]	1	Incident	Individual	Medical records linkage + neurologist chart review	Occupational or hobby, M&W
2008	Brighina [158]	1	Incident	Individual	Medical records linkage + neurologist chart review	Occupational or residential
2009	Costello [68]	1	Incident	Individual	Neurologist examination	Occupational, likely
2009	Gatto [69]*	1	Incident	Individual	Neurologist examination	≥ 12 of 26 pesticides in well water
2009	Ritz [70]*	1	Incident	Individual	Neurologist examination	Occupational, likely
2010	Firestone [57]†	1	Incident	Individual	Neurologist chart review	Occupational, men
2010	Firestone [57]†	1	Incident	Individual	Neurologist chart review	Occupational, women
2010	Skeie [140]	1	Incident	Individual	Neurological examination	Occupational or private
2010	Vlajinac [115]	1	Incident	Individual	Neurologist diagnosis	Occupational and residential
2011	Feldman [73]	1	Incident	Individual	Hospital discharge and cause-of-death registers	Occupational
1989	Ho [116]	2	Prevalent	Individual	Neurological examination and confirmation	Previous use
1990	Hertzman [141]	2	Prevalent	Individual	Neurologist examination	Ever handled 11 [illegible]
1990	Koller [118]	2	Prevalent	Individual	Neurologist examination	Ever use
1992	Jiménez-J. [120]	2	Prevalent	Individual	Neurology clinic attendance	Direct or indirect contact ≥ 1 yr
1992	Semchuk [143]	2	Prevalent	Individual	Neurologist confirmation	Occupational
1993	Hubble [54]	2	Prevalent	Individual	Neurologist examination	Factor: > 20 d/yr ever or > 5 yrs
1994	Hertzman [132]	2	Prevalent	Individual	Neurologist examination	Occupational - men, voter ctrls.
1994	Morano [100]	2	Prevalent	Individual	Neurology clinic attendance	Direct or indirect exposure ≥ 1 yr
1995	Chaturvedi [19]	2	Prevalent	Individual	Self-report	Occupational
1997	Liou [59]	2	Prevalent	Individual	Neurological examination	Occupational or residential
1998	Chan [79]	2	Prevalent	Individual	Neurologist/geriatrician examination	During farming, M&W multivar.
1998	De Palma [123]*	2	Prevalent	Individual	Neurology clinic attendance	Occupational or residential ≥ 10 yrs
1998	McCann [125]	2	Prevalent	Individual	"All participants were reviewed"	Daily or weekly for > 6 mos.
1998	Smargiassi [134]	2	Prevalent	Individual	Neurology clinic attendance	Occupational for ≥ 10 consec. yrs
1999	Fall [80]	2	Prevalent	Individual	Neurologist examination or chart review	Handling for any occup., men
1999	Kuopio [81]	2	Prevalent	Individual	Neurologist examination	Regular and occasional use
1999	Taylor [102]	2	Prevalent	Individual	Neurologist examination	Years of use
1999	Werneck [126]	2	Prevalent	Individual	Neurologist examination	Inhaling/handling herb/pest ≥ 15 yrs
2000	Preux [127]	2	Prevalent	Individual	Neurologist examination	Work or leisure contact
2001	Engel [135]	2	Prevalent	Individual	Neurological examination	Any

Table F: Metadata for Tier 1 and Tier 2 studies: Pesticide use (continued)

Year	Author	Tier	Case Type	Exposure Type	Diagnostic Data	Exposure
2002	Zorzon [128]	2	Prevalent	Individual	Neurological examination	Exposure
2003	Baldereschi [107]	2	Prevalent	Individual	Neurological examination	Pesticide-use license
2003	Baldi [149]	2	Prevalent	Individual	Neurologist referral	Occupational
2003	Dong [84]	2	Prevalent	Individual	Prevalence survey + 46% neurologist confirmation	Use
2003	Duzcan [150]	2	Prevalent	Individual	Neurologist examination and confirmation	Exp. > 20 days/yr for ≥ 10 yrs
2005	Galanaud [87]*	2	Prevalent	Individual	Neurologist examination or confirmation	Gardening or professional
2005	Park RM [151]	2	Deceased	Individual	Death certificate	Probable occupational
2005	Wright [129]	2	Prevalent	Individual	Support group or exercise group attendance	Occupational
2007	Dick [136]	2	Prevalent	Individual	Neurologist confirmation or chart review	Occupational or hobby, any
2007	Fong [89]	2	Prevalent	Individual	Neurologist examination	Use
2007	Kamel [18]	2	Incident	Individual	Self-report	Ever use, incident PD
2007	Kamel [18]*	2	Prevalent	Individual	Self-report	Ever use, prevalent PD
2008	Dhillon [92]	2	Prevalent	Individual	Neurologist diagnosis	Agricultural pesticides in past yr
2008	Hancock [137]	2	Prevalent	Individual	Neurologist examination	Ever applied
2008	Petersen [91]	2	Prevalent	Individual	Neurologist examination	Occupational, ever
2009	Elbaz [55]	2	Prevalent	Individual	Neurologist examination or confirmation	Professional, both sexes
2009	Tanner [154]	2	Prevalent	Individual	Neurologist diagnosis	Occupational
2010	Kiyohara [94]	2	Prevalent	Individual	Neurologist diagnosis	Occupational or home
2010	Sanyal [130]	2	Prevalent	Individual	Neurologist examination	Exposure for ≥ 5 yrs
2011	Das [58]	2	Prevalent	Individual	Neurologist confirmation	≥ 3 hrs/day, > 3 mos/yr for 10 yrs
2011	Rugbjerg [156]	2	Prevalent	Individual	Neurological examination and neurologist review	Hygiene-reviewed, any operation
2011	Tanaka [155]*	2	Prevalent	Individual	Neurologist diagnosis	Occupational for ≥ 10 hrs/wk, > 1 yr

*Estimate excluded from meta-analysis due to overlap with another estimate ([57] with [73], [69] with [70, 99], [55] with [142], [149] with [203], [184] with [172], and dual estimates from [18]).
†Independent stratum-specific estimates obtained from the same study and used in the meta-analysis.
Results from Pals et al. [160] were not included because relative risks and confidence intervals could not be calculated, but the authors reported: "With the exception of a trend towards more organophosphates exposure in patients, we found no differences in pesticide exposure."

Table G: Metadata for Tier 1 and Tier 2 studies: Herbicide, fungicide or insecticide use

Year	Author	Tier	Case Type	Exposure Type	Diagnostic Data	Exposure
**Herbicides**						
2005	Firestone [114]	1	Incident	Individual	Neurologist chart review	Occupational, men
2006	Frigerio [157]	1	Incident	Individual	Medical records linkage + neurologist chart review	With farming, men
2008	Brighina [158]	1	Incident	Individual	Medical records linkage + neurologist chart review	Ever/never
2010	Vlajinac [115]	1	Incident	Individual	Neurologist diagnosis	Use
1991	Stern [119]	2	Prevalent	Individual	Neurology clinic attendance and chart review	Any exposure vs. none
1992	Semchuk [143]	2	Prevalent	Individual	Neurologist confirmation	Herb. / insect. / fung. - agricultural
1994	Hertzman [132]	2	Prevalent	Individual	Neurologist examination	Men
1996	Seidler [121]	2	Prevalent	Individual	Neurologist confirmation	Neighbors, stratified sample
1998	Gorell [122]	2	Prevalent	Individual	Neurologist diagnosis or examination	All occupations
1999	Taylor [102]	2	Prevalent	Individual	Neurologist examination	Years of use
1999	Kuopio [81]	2	Prevalent	Individual	Neurologist examination	Regular and occasional use
2001	Behari [104]	2	Prevalent	Individual	Neurological examination	Exposure, matched analysis
2001	Engel [135]	2	Prevalent	Individual	Neurological examination	Any
2008	Dhillon [92]	2	Prevalent	Individual	Neurologist diagnosis	Home/agricultural
2008	Hancock [137]	2	Prevalent	Individual	Neurologist examination	Ever applying
2009	Elbaz [55]	2	Prevalent	Individual	Neurologist examination or confirmation	Use, both sexes
2011	Tanaka [155]	2	Prevalent	Individual	Neurologist diagnosis	Occupational
2011	Rugbjerg [156]	2	Prevalent	Individual	Neurological examination and neurologist review	Hygiene-reviewed, any operation
**Fungicides**						
2005	Firestone [114]	1	Incident	Individual	Neurologist chart review	Occupational, men
2008	Brighina [158]	1	Incident	Individual	Medical records linkage + neurologist chart review	Ever/never
2010	Vlajinac [115]	1	Incident	Individual	Neurologist diagnosis	Use
1992	Semchuk [143]	2	Prevalent	Individual	Neurologist confirmation	Use
1994	Hertzman [132]	2	Prevalent	Individual	Neurologist examination	Men
1998	Gorell [122]	2	Prevalent	Individual	Neurologist diagnosis or examination	All occupations
2001	Engel [135]	2	Prevalent	Individual	Neurological examination	Any
2009	Elbaz [55]	2	Prevalent	Individual	Neurologist examination or confirmation	Men
2011	Tanaka [155]	2	Prevalent	Individual	Neurologist diagnosis	Occupational
2011	Rugbjerg [156]	2	Prevalent	Individual	Neurological examination and neurologist review	Hygiene-reviewed, any operation
**Insecticides**						
2005	Firestone [114]	1	Incident	Individual	Neurologist chart review	Occupational, men
2006	Frigerio [157]	1	Incident	Individual	Medical records linkage + neurologist chart review	Farming, any exposure, men
2008	Brighina [158]	1	Incident	Individual	Medical records linkage + neurologist chart review	Ever/never
2010	Vlajinac [115]	1	Incident	Individual	Neurologist diagnosis	Occupational and residential

Table G: Metadata for Tier 1 and Tier 2 studies: Herbicide, fungicide or insecticide use (Insecticides, continued)

Year	Author	Tier	Case Type	Exposure Type	Diagnostic Data	Exposure
1991	Stern [119]	2	Prevalent	Individual	Neurology clinic attendance and chart review	Use; any vs. none
1992	Semchuk [143]	2	Prevalent	Individual	Neurologist confirmation	Use
1994	Hertzman [132]	2	Prevalent	Individual	Neurologist examination	Men - exposure
1996	Seidler [121]	2	Prevalent	Individual	Neurologist confirmation	Neighbors, age > 80 years
1998	Gorell [122]	2	Prevalent	Individual	Neurologist diagnosis or examination	All occupations
1999	Fall [80]	2	Prevalent	Individual	Neurologist examination or chart review	Handling for agriculture, men
2001	Behari [104]	2	Prevalent	Individual	Neurological examination	Exposure, matched analysis
2001	Engel [135]	2	Prevalent	Individual	Neurological examination	Any
2008	Hancock [137]	2	Prevalent	Individual	Neurologist examination	Ever applied
2008	Dhillon [92]	2	Prevalent	Individual	Neurologist diagnosis	Farm crops
2009	Elbaz [55]	2	Prevalent	Individual	Neurologist examination or confirmation	Men
2011	Rugbjerg [156]	2	Prevalent	Individual	Neurological examination and neurologist review	Hygiene-reviewed, any operation
2011	Das [58]	2	Prevalent	Individual	Neurologist confirmation	Exposure to pesticides

Table H: Metadata for Tier 1 and Tier 2 studies: High herbicide, fungicide or insecticide use

Year	Author	Tier	Case Type	Exposure Type	Diagnostic Data	Exposure
**High Herbicides**						
2010	Vlajinac [115]	1	Incident	Individual	Neurologist diagnosis	Exposure ≥ 30 yrs
1992	Semchuk [143]	2	Prevalent	Individual	Neurologist confirmation	Use 46-55 years
1993	Butterfield [98]	2	Prevalent	Individual	Neurologist confirmation	Exposure > 10 times / yr crude calc.
1996	Seidler [121]	2	Prevalent	Individual	Neurologist confirmation	>80 dose yrs vs. neighbor controls
1999	Kuopio [81]	2	Prevalent	Individual	Neurologist examination	Regular use
2001	Engel [135]	2	Prevalent	Individual	Neurological examination	Any, tertile 3 [1,201-10,345 acre-yrs]
2003	Duzcan [150]	2	Prevalent	Individual	Neurologist examination and confirmation	Exp. > 20 days/yr for ≥ 10 yrs
2009	Elbaz [55]†	2	Prevalent	Individual	Neurologist examination or confirmation	Men, ≤65 yrs [# cumul. hrs ≥median]
2009	Elbaz [55]†	2	Prevalent	Individual	Neurologist examination or confirmation	Men, >65 yrs [# cumul. hrs ≥median]
2010	Sanyal [130]	2	Prevalent	Individual	Neurologist examination	Exposure for ≥ 5 yrs
**High Fungicides**						
2010	Vlajinac [115]	1	Incident	Individual	Neurologist diagnosis	Exposure ≥ 10 yrs
2001	Engel [135]	2	Prevalent	Individual	Neurological examination	Any, tertile 3 [1,201-10,900 acre-yrs]
2003	Duzcan [150]	2	Prevalent	Individual	Neurologist examination and confirmation	Exp. > 20 days/yr for ≥ 10 yrs
2009	Elbaz [55]†	2	Prevalent	Individual	Neurologist examination or confirmation	Men, ≤65 yrs [# cumul. hrs ≥median]
2009	Elbaz [55]†	2	Prevalent	Individual	Neurologist examination or confirmation	Men, >65 yrs [# cumul. hrs ≥median]
**High Insecticides**						
2010	Vlajinac [115]	1	Incident	Individual	Neurologist diagnosis	Exposure ≥ 30 yrs
1992	Semchuk [143]	2	Prevalent	Individual	Neurologist confirmation	Use 46-55 years
1993	Butterfield [98]	2	Prevalent	Individual	Neurologist confirmation	Exp. (>10 times/yr) [model 3]
1996	Seidler [121]	2	Prevalent	Individual	Neurologist confirmation	>80 dose yrs vs. neighbor controls
2001	Engel [135]	2	Prevalent	Individual	Neurological examination	Any, tertile 3 [1,551-16,140 acre-yrs]
2003	Duzcan [150]	2	Prevalent	Individual	Neurologist examination and confirmation	Exp. > 20 days/yr for ≥ 10 yrs
2009	Elbaz [55]†	2	Prevalent	Individual	Neurologist examination or confirmation	Men, ≤65 yrs [# cumul. hrs ≥median.]
2009	Elbaz [55]†	2	Prevalent	Individual	Neurologist examination or confirmation	Men, >65 yrs [# cumul. hrs ≥median.]

† Independent stratum-specific estimates obtained from the same study and used in the meta-analysis.

Table I: Metadata for Tier 1 and Tier 2 studies: Paraquat ever-use or high-use

Year	Author	Tier	Case Type	Exposure Type	Diagnostic Data	Exposure
**Ever Use of Paraquat**						
2005	Firestone [114]*	1	Incident	Individual	Neurologist chart review	Occupational, men
2010	Firestone [57]	1	Incident	Individual	Neurologist chart review	Occupational, men
1994	Hertzman [132]	2	Prevalent	Individual	Neurologist examination	Voter controls
1997	Liou [59]	2	Prevalent	Individual	Neurological examination	Use
1999	Kuopio [81]	2	Prevalent	Individual	Neurologist examination	Use; calc. using comp. meta.
2001	Engel [135]	2	Prevalent	Individual	Neurological examination	Occupational
2004	Elbaz [164]*	2	Prevalent	Individual	Neurologist examination or confirmation	Use, men
2007	Kamel [18]	2	Incident	Individual	Self-report	Incident PD cases
2007	Kamel [18]*	2	Prevalent	Individual	Self-report	Prevalent PD cases
2008	Dhillon [92]	2	Prevalent	Individual	Neurologist diagnosis	Ever
2009	Costello [68]*	2	Incident	Ecologic	Neurologist examination	Only 1974-1999
2009	Elbaz [55]	2	Prevalent	Individual	Neurologist examination or confirmation	Use, all men, multiple
2009	Gatto [69]*	2	Incident	Ecologic	Neurologist examination	Use
2009	Tanner [154]	2	Prevalent	Individual	Neurologist diagnosis	Use
2011	Wang [162]*	2	Incident	Ecologic	Neurologist examination	Residential and occupational
2011	Rugbjerg [156]	2	Prevalent	Individual	Neurological examination and neurologist review	Exposure
2011	Tanner [160]	2	Prevalent	Individual	Neurologist examination	Ever
2011	Tomenson [165]	2	Deceased	Individual	Death certificate	Occupational (production)
2012	Goldman [163]*	2	Prevalent	Individual	Neurologist examination	Ever, men
2012	Lee [161]	2	Incident	Ecologic	Neurologist examination	Residential and workplace
**High Use of Paraquat**						
1997	Liou [59]	2	Prevalent	Individual	Neurological examination	≥20 yrs of use
2001	Engel [135]	2	Prevalent	Individual	Neurological examination	683-9,950 acre-yrs
2009	Gatto [69]	2	Incident	Ecologic	Neurologist examination	High exposure level
2012	Goldman [163]	2	Prevalent	Individual	Neurologist examination	Lifetime use > median= 4 yrs

*Estimate excluded from meta-analysis due to overlap with another estimate ([57] with [73], [68] with [69-71], [67] with [72], [55] with [74], and dual estimates from [18]).

The following four studies were not included because relative risks and confidence intervals could not be calculated, but relevant data were provided. Semchuk et al. [193] noted in a case-control study of 130 cases and 260 controls that one case reported having worked with paraquat (not specified for controls). Pals et al. [160] found no cases or controls that used paraquat in a case-control study of 403 PD cases and 205 controls. Seidler et al. [170] found in a case-control study of 380 PD cases, 379 neighborhood controls and 376 regional controls that one case and zero controls reported use of paraquat. In a case-control study of 57 cases and 122 controls, Hertzman et al. [191] found that four cases and zero controls reported use of paraquat (p value = .01).
