# Supplementary material for: Association between Parkinson’s Disease and Cigarette Smoking, Rural Living, Well-Water Consumption, Farming and Pesticide Use: Systematic Review and Meta-Analysis
Source: PLoS One. 2016 Apr 7;11(4):e0151841. doi: 10.1371/journal.pone.0151841 (PMC4824443; doi:10.1371/journal.pone.0151841)
Supplement: S4 File — Tables A to N: Normalized study weights based upon fixed and random effects models and RRs and 95% CIs for individual Tier 1 or Tier 2 studies. Table A: RRs, 95% CIs and fixed or random effects study weights for Tier 1 or Tier 2 studies: Current cigarette smoking. Table B: RRs, 95% CIs and fixed or random effects study weights for Tier 1 or Tier 2 studies: Heavy or long-term cigarette smoking. Table C: RRs, 95% CIs and fixed or random effects study weights for Tier 1 or Tier 2 studies: Rural living. Table D: RRs, 95% CIs and fixed or random effects study weights for Tier 1 or Tier 2 studies: Well-water consumption. Table E: RRs, 95% CIs and fixed or random effects study weights for Tier 1 or Tier 2 studies: Farming. Table F: RRs, 95% CIs and fixed or random effects study weights for Tier 1 or Tier 2 studies: Pesticide use. Table G: RRs, 95% CIs and fixed or random effects study weights for Tier 1 or Tier 2 studies: Herbicide use. Table H: RRs, 95% CIs and fixed or random effects study weights for Tier 1 or Tier 2 studies: Fungicide use. Table I: RRs, 95% CIs and fixed or random effects study weights for Tier 1 or Tier 2 studies: Insecticide use. Table J: RRs, 95% CIs and fixed or random effects study weights for Tier 1 or Tier 2 studies: High herbicide use. Table K: RRs, 95% Cls and fixed or random effects study weights for Tier 1 or Tier 2 studies: High fungicide use. Table L: RRs, 95% CIs and fixed or random effects study weights for Tier 1 or Tier 2 studies: High insecticide use. Table M: RRs, 95% CIs and fixed or random effects study weights for Tier 1 or Tier 2 studies: Paraquat use. Table N: RRs, 95% CIs and fixed or random effects study weights for Tier 1 or Tier 2 studies: High paraquat use. (PDF) [file pone.0151841.s006.pdf]

Table A: RRs, 95% CIs and fixed or random effects study weights: Current cigarette smoking

Author	Year	Tier	RR	LCL	UCL	Fixed Effects Normalized Study Weight	Random Effects Normalized Study Weight
Benedetti [60]	2000	1	1.14	0.41	3.15	2.38	5.91
Hernán [61]	2001	1	0.4	0.2	0.6	8.21	10.09
Checkoway [62]	2002	1	0.3	0.1	0.7	2.62	6.23
Wirdefeldt [64]	2005	1	0.56	0.4	0.79	21.39	12.29
Park [63]	2005	1	0.2	0.12	0.34	9.14	10.40
Thacker [65]	2007	1	0.27	0.13	0.56	4.65	8.26
Tan [66]	2008	1	0.29	0.16	0.52	7.13	9.67
Sääksjärvi [67]	2008	1	0.19	0.07	0.52	2.46	6.02
Costello [68]	2009	1	0.48	0.27	0.86	7.38	9.77
Shino [72]	2010	1	0.13	0.07	0.28	5.16	8.61
Liu [74]	2012	1	0.56	0.42	0.75	29.48	12.76
						100%	100%
**Meta-Analysis (Fixed)**			0.41*	0.35	0.48		
**Meta-Analysis (Random)**			0.35*	0.26	0.48		
Mayeux [75]	1994	2	0.2	0.1	0.5	2.23	4.04
Martyn [76]	1995	2	0.5	0.28	0.93	4.01	5.34
Tzourio [78]	1997	2	0.7	0.4	1.3	4.16	5.42
Hellenbrand [77]	1997	2	0.2	0.1	0.3	4.79	5.71
Chan [79]	1998	2	0.51	0.26	1.01	3.14	4.80
Kuopio [81]	1999	2	0.5	0.2	1.24	1.74	3.49
Fall [80]	1999	2	0.17	0.06	0.43	1.49	3.17
Paganini-Hill [82]	2001	2	0.42	0.25	0.69	5.61	6.03
Tsai [83]	2002	2	0.3	0.03	3.11	0.27	0.81
Ragonese [85]	2003	2	0.87	0.46	1.65	3.55	5.07
Dong [84]	2003	2	0.44	0.23	0.86	3.33	4.93
Ascherio [3]	2004	2	0.66	0.54	0.81	35.18	8.30
Galanaud [87]	2005	2	0.5	0.2	1	2.23	4.04
Fong [89]	2007	2	0.86	0.45	1.64	3.46	5.01
Kamel [18]	2007	2	0.6	0.2	1.7	1.26	2.84
Hancock [88]	2007	2	0.3	0.17	0.53	4.47	5.57
Dhillon [92]	2008	2	1.1	0.3	5.2	0.71	1.86
Petersen [91]	2008	2	0.63	0.26	1.55	1.81	3.58
Powers [90]	2008	2	0.45	0.29	0.7	7.45	6.56
Nicoletti [95]	2010	2	0.37	0.22	0.64	5.07	5.83
Tanaka [93]	2010	2	0.12	0.05	0.27	2.03	3.83
Kyrozis [20]	2013	2	0.42	0.18	0.99	1.99	3.79
						100%	100%
**Meta-Analysis (Fixed)**			0.49*	0.44	0.56		
**Meta-Analysis (Random)**			0.44*	0.35	0.55		

*Statistically significant (P < 0.05; 95% CI excludes 1.0).

Table B: RRs, 95% CIs and fixed or random effects study weights: Heavy or long-term cigarette smoking

Author	Year	Tier	RR	LCL	UCL	Fixed Effects Normalized Study Weight	Random Effects Normalized Study Weight
Grandinetti [96]	1994	1	0.77	0.66	0.9	40.07	17.66
Benedetti [60]	2000	1	0.69	0.32	1.48	1.64	5.00
Hernán [61]	2001	1	0.3	0.2	0.6	3.19	7.85
Checkoway [62]	2002	1	0.4	0.2	0.8	2.01	5.78
Wirdefeldt [64]	2005	1	0.52	0.29	0.95	2.74	7.13
Thacker [65]	2007	1	0.55	0.35	0.86	4.77	9.81
Tan [66]	2008	1	0.18	0.07	0.45	1.11	3.69
Costello [68]	2009	1	0.54	0.38	0.78	7.45	11.99
Chen [71]	2010	1	0.57	0.44	0.74	14.26	14.77
Liu [74]	2012	1	0.68	0.55	0.83	22.76	16.32
						100%	100%
**Meta-Analysis (Fixed)**			0.64*	0.58	0.71		
**Meta-Analysis (Random)**			0.55*	0.45	0.67		
Sasco [97]	1990	2	0.27	0.08	0.96	0.61	1.79
Wang [99]	1993	2	0.5	0.23	1.09	1.56	3.23
Butterfield [98]	1993	2	0.32	0.15	0.67	1.68	3.36
Mayeux [75]	1994	2	0.6	0.3	1.2	1.96	3.62
Morano [100]	1994	2	0.23	0.04	1.2	0.33	1.09
Liou [59]	1997	2	0.43	0.2	0.9	1.67	3.35
Hellenbrand [77]	1997	2	0.3	0.2	0.6	3.13	4.37
Taylor [102]	1999	2	0.82	0.66	0.9	39.23	6.43
Fall [80]	1999	2	0.31	0.11	0.78	0.98	2.48
Vanacore [103]	2000	2	0.32	0.15	0.66	1.72	3.40
Behari [104]	2001	2	0.74	0.46	1.19	4.18	4.79
Paganini-Hill [82]	2001	2	0.42	0.22	0.8	2.26	3.86
Ragonese [85]	2003	2	0.72	0.29	1.01	2.42	3.97
Baldereschi [107]	2003	2	0.6	0.32	1.12	2.40	3.95
Pals [106]	2003	2	0.44	0.23	0.82	2.34	3.91
Tan [105]	2003	2	0.38	0.2	0.72	2.30	3.88
Dong [84]	2003	2	0.35	0.18	0.7	2.05	3.69
Gorell [108]	2004	2	0.42	0.25	0.71	3.46	4.52
Ma [110]	2006	2	3.41	1.2	7.74	1.09	2.64
Evans [109]	2006	2	0.33	0.12	0.92	0.91	2.36
Kamel [18]	2007	2	1	0.4	2.3	1.23	2.84
Hancock [88]	2007	2	0.35	0.2	0.62	2.95	4.28
Petersen [91]	2008	2	0.53	0.26	1.08	1.86	3.53
Powers [90]	2008	2	0.44	0.31	0.64	7.18	5.44
Elbaz [55]	2009	2	0.4	0.2	0.7	2.40	3.95
Nicoletti [95]	2010	2	0.39	0.26	0.6	5.40	5.12
Tanaka [93]	2010	2	0.28	0.15	0.49	2.69	4.14
						100%	100%
**Meta-Analysis (Fixed)**			0.57*	0.52	0.63		
**Meta-Analysis (Random)**			0.47*	0.39	0.57		

*Statistically significant (P < 0.05; 95% CI excludes 1.0).

Table C: RRs, 95% CIs and fixed or random effects study weights: Rural living

Author	Year	Tier	RR	LCL	UCL	Fixed Effects Normalized Study Weight	Random Effects Normalized Study Weight
Baldi [56]	2003	1	1.37	0.56	3.33	6.87	18.48
Wirdefeldt [64]	2005	1	0.92	0.66	1.28	49.75	29.61
Firestone [114]	2005	1	1.31	0.84	2.03	28.03	27.56
Vlajinac [115]	2010	1	3.56	1.96	6.46	15.35	24.35
						100%	100%
**Meta-Analysis (Fixed)**			1.28*	1.02	1.62		
**Meta-Analysis (Random)**			1.52	0.85	2.71		
Tanner [117]	1989	2	0.57	0.33	0.98	1.19	4.08
Ho [116]	1989	2	4.9	1.4	18.2	0.22	1.45
Koller [118]	1990	2	1.88	1.13	3.19	1.31	4.23
Stern [119]	1991	2	1.7	0.9	3.1	0.93	3.66
Jiménez-J. [120]	1992	2	1.07	0.69	1.63	1.92	4.80
Wang [99]	1993	2	0.76	0.49	1.18	1.83	4.73
Hubble [54]	1993	2	2.25	0.6	8.42	0.20	1.38
Butterfield [98]	1993	2	2.35	0.87	6.34	0.36	2.12
Hubble [54]	1993	2	6.49	2.35	17.9	0.34	2.05
Morano [100]	1994	2	1.47	0.79	2.71	0.93	3.67
Martyn [76]	1995	2	1.4	0.82	2.49	1.15	4.01
Seidler [121]	1996	2	0.83	0.34	2	0.45	2.46
Liou [59]	1997	2	2.04	1.23	3.38	1.39	4.32
Marder [124]	1998	2	0.8	0.32	1.98	0.43	2.37
Gorell [122]	1998	2	1.19	0.73	1.93	1.50	4.44
McCann [125]	1998	2	1.7	1.17	2.57	2.29	5.04
De Palma [123]	1998	2	3.62	2.09	6.26	1.18	4.05
Werneck [126]	1999	2	1	0.52	1.95	0.81	3.43
Taylor [102]	1999	2	1.07	0.99	1.15	63.06	6.69
Preux [127]	2000	2	1.67	1	2.5	1.69	4.61
Behari [104]	2001	2	0.94	0.7	1.25	4.21	5.71
Zorzon [128]	2002	2	1.5	1	2.4	1.85	4.75
Wright [129]	2005	2	1.1	0.8	1.3	6.00	5.99
Sanyal [130]	2010	2	4.05	2.53	6.49	1.59	4.53
Das [58]	2011	2	1.05	0.75	1.46	3.19	5.43
						100%	100%
**Meta-Analysis (Fixed)**			1.16*	1.09	1.23		
**Meta-Analysis (Random)**			1.43*	1.20	1.70		

*Statistically significant (P < 0.05; 95% CI excludes 1.0).

Table D: RRs, 95% CIs and fixed or random effects study weights: Well-water consumption

Author	Year	Tier	RR	LCL	UCL	Fixed Effects Normalized Study Weight	Random Effects Normalized Study Weight
Park [131]	2004	1	0.62	0.29	1.32	8.44	14.12
Park [63]	2005	1	1.71	1.14	2.45	33.11	25.04
Firestone [114]	2005	1	1.81	1.02	3.21	14.75	18.83
Gatto [69]	2009	1	1.21	0.82	1.8	31.35	24.67
Vlajinac [115]	2010	1	2.62	1.4	4.9	12.35	17.33
						100%	100%
**Meta-Analysis (Fixed)**			1.50*	1.20	1.87		
**Meta-Analysis (Random)**			1.48*	1.02	2.15		
Tanner [117]	1989	2	0.74	0.41	1.32	0.61	3.15
Koller [118]	1990	2	1.67	1.01	2.79	0.80	3.52
Stern [119]	1991	2	0.8	0.4	1.6	0.43	2.68
Jiménez-J. [120]	1992	2	1.22	0.77	1.94	0.97	3.76
Wang [99]	1993	2	0.59	0.36	0.95	0.88	3.64
Hertzman [132]	1994	2	0.9	0.52	1.55	0.70	3.33
Morano [100]	1994	2	3.28	0.93	11.51	0.13	1.23
Seidler [121]	1996	2	0.8	0.6	1.2	1.73	4.37
De Michele [133]	1996	2	2.17	1.28	3.69	0.74	3.41
Liou [59]	1997	2	1.07	0.19	5.98	0.07	0.73
McCann [125]	1998	2	0.6	0.38	0.92	1.06	3.86
Gorell [122]	1998	2	0.97	0.65	1.4	1.41	4.17
Chan [79]	1998	2	1.04	0.7	1.54	1.34	4.12
Marder [124]	1998	2	1.79	1.04	3.1	0.70	3.33
Smargiassi [134]	1998	2	2.78	1.46	5.28	0.50	2.89
Taylor [102]	1999	2	0.93	0.88	0.98	71.73	5.50
Kuopio [81]	1999	2	0.97	0.59	1.6	0.83	3.57
Werneck [126]	1999	2	1.49	0.74	3.01	0.42	2.65
Preux [127]	2000	2	1.19	0.77	1.84	1.10	3.90
Engel [135]	2001	2	0.9	0.6	1.5	0.99	3.78
Behari [104]	2001	2	1.94	1.33	2.8	1.50	4.23
Zorzon [128]	2002	2	2	1.1	3.6	0.59	3.11
Tsai [83]	2002	2	10.9	1.77	67.5	0.06	0.66
Dong [84]	2003	2	0.96	0.48	1.92	0.43	2.68
Wright [129]	2005	2	8.3	2.5	27.6	0.14	1.32
Dick [136]	2007	2	1.23	1	1.52	4.74	5.04
Hancock [137]	2008	2	1.08	0.77	1.5	1.87	4.44
Elbaz [55]	2009	2	1	0.7	1.5	1.43	4.19
Sanyal [130]	2010	2	4.5	2.1	9.9	0.35	2.37
Das [58]	2011	2	2.5	1.77	3.54	1.73	4.37
						100%	100%
**Meta-Analysis (Fixed)**			1.01	0.96	1.05		
**Meta-Analysis (Random)**			1.27*	1.08	1.49		

*Statistically significant ($P < 0.05$; 95% CI excludes 1.0).

Table E: RRs, 95% CIs and fixed or random effects study weights: Farming

Author	Year	Tier	RR	LCL	UCL	Fixed Effects Normalized Study Weight	Random Effects Normalized Study Weight
Park [131]	2004	1	0.27	0.06	1.1	1.95	2.86
Frigerio [138]	2005	1	1.5	0.8	2.5	12.71	13.26
Park [63]	2005	1	1.64	0.96	2.81	14.31	14.32
Ascherio [139]	2006	1	1.6	0.9	2.7	13.67	13.91
Firestone [57]	2010	1	1.06	0.85	3.01	10.32	11.51
Vlajinac [115]	2010	1	1.31	0.68	2.52	9.62	10.94
Skeie [140]	2010	1	1.75	1.03	3	14.44	14.40
Feldman [73]	2011	1	0.9	0.6	1.4	22.99	18.80
						100%	100%
**Meta-Analysis (Fixed)**			1.28*	1.05	1.57		
**Meta-Analysis (Random)**			1.28	0.99	1.66		
Ho [116]	1989	2	5.2	1.6	17.7	0.08	0.74
Koller [118]	1990	2	1.33	0.85	2.11	0.16	1.32
Hertzman [141]	1990	2	2.98	1.28	6.97	0.56	2.91
Tanner [142]	1990	2	3	1	9.8	0.09	0.81
Semchuk [143]	1992	2	1.94	1.12	3.34	0.39	2.39
Hertzman [132]	1994	2	0.72	0.22	2.3	0.08	0.77
Chaturvedi [19]	1995	2	1.42	0.86	2.37	0.45	2.60
Rocca [144]	1996	2	0.6	0.3	1.3	0.22	1.63
Seidler [121]	1996	2	0.9	0.6	1.4	0.65	3.11
Liou [59]	1997	2	1.81	1.25	2.64	0.83	3.46
Chan [79]	1998	2	0.92	0.59	1.43	0.59	2.99
Smargiassi [134]	1998	2	1.25	0.65	2.43	0.75	3.32
Gorell [122]	1998	2	1.3	0.88	1.93	0.04	0.38
Marder [124]	1998	2	13.6	2.4	79.5	0.27	1.89
Tsui [145]	1999	2	0.68	0.32	1.41	0.22	1.66
Fall [80]	1999	2	1.4	0.68	2.9	0.46	2.62
Kuopio [81]	1999	2	1.45	0.88	2.41	0.21	1.61
Preux [127]	2000	2	1.06	0.71	1.59	0.72	3.25
Tuchsen [146]	2000	2	1.32	1.11	1.56	4.01	5.02
Behari [104]	2001	2	0.72	0.48	1.05	0.76	3.33
Engel [135]	2001	2	1	0.5	1.8	0.28	1.97
Lee [148]	2002	2	0.86	0.81	0.92	28.67	5.59
Zorzon [128]	2002	2	7.7	1.4	44.1	0.04	0.39
Baldi [149]	2003	2	0.88	0.44	1.56	0.46	2.62
Dong [84]	2003	2	0.75	0.42	1.32	0.29	2.00
Baldereschi [107]	2003	2	1.2	0.72	1.97	0.35	2.26
Duzcan [150]	2003	2	1.69	0.78	3.65	0.20	1.52
Wright [129]	2005	2	1	0.4	2.8	0.97	3.66
Park [151]	2005	2	1.14	1.08	1.19	49.41	5.63
Goldman [152]	2005	2	3	2.1	4.2	0.12	1.06
Dick [153]	2007	2	1.02	0.82	1.28	2.34	4.63
Dhillon [92]	2008	2	1.1	0.6	2	0.32	2.13
Hancock [137]	2008	2	1.11	0.8	1.54	1.08	3.81
Tanner [154]	2009	2	1.1	0.78	1.57	0.72	3.26

Table E: RRs, 95% CIs and fixed or random effects study weights: Farming (continued)

Author	Year	Tier	RR	LCL	UCL	Fixed Effects Normalized Study Weight	Random Effects Normalized Study Weight
Elbaz [55]	2009	2	1.9	1.3	2.9	0.95	3.64
Sanyal [130]	2010	2	2.01	0.14	27.93	0.02	0.17
Tanaka [155]	2011	2	0.95	0.41	2.15	1.31	4.04
Das [58]	2011	2	1.08	0.8	1.45	0.21	1.62
Rugbjerg [156]	2011	2	2.47	1.18	5.15	0.17	1.36
Kyrozis [20]	2013	2	0.97	0.61	1.55	0.53	2.84
						100%	100%
**Meta-Analysis (Fixed)**			1.07*	1.04	1.11		
**Meta-Analysis (Random)**			1.23*	1.10	1.38		

*Statistically significant (P < 0.05; 95% CI excludes 1.0).

Table F: RRs, 95% CIs and fixed or random effects study weights: Pesticide use

Author	Year	Tier	RR	LCL	UCL	Fixed Effects Normalized Study Weight	Random Effects Normalized Study Weight
Baldi [56]	2003	1	5.63	1.47	21.5	1.02	3.41
Baldi [56]	2003	1	1.02	0.22	4.82	0.77	2.71
Ascherio [139]	2006	1	1.8	1.3	2.5	17.19	14.25
Frigerio [157]	2006	1	1.5	0.8	2.9	4.43	9.05
Brighina [158]	2008	1	1.11	0.89	1.38	38.21	16.01
Costello [68]	2009	1	1.52	1.08	2.14	15.72	13.99
Firestone [57]	2010	1	0.6	0.30	1.29	3.46	7.94
Firestone [57]	2010	1	3.9	0.39	39.4	0.35	1.32
Skeie [140]	2010	1	1.06	0.62	1.82	6.34	10.62
Vlajinac [115]	2010	1	3.44	1.81	6.53	4.47	9.08
Feldman [73]	2011	1	0.9	0.5	1.3	8.05	11.62
						100%	100%
**Meta-Analysis (Fixed)**			1.32*	1.16	1.52		
**Meta-Analysis (Random)**			1.40*	1.06	1.85		
Ho [116]	1989	2	3.6	1.0	12.9	0.10	1.06
Hertzman [141]	1990	2	1.34	0.71	2.52	0.40	2.60
Koller [118]	1990	2	1.05	0.67	1.65	0.79	3.39
Jiménez-J. [120]	1992	2	1.34	0.85	2.13	0.76	3.35
Semchuk [143]	1992	2	2.25	1.27	3.99	0.49	2.85
Hubble [54]	1993	2	3.42	1.27	7.32	0.21	1.82
Hertzman [132]	1994	2	2.32	1.10	4.88	0.29	2.20
Morano [100]	1994	2	1.73	0.98	3.03	0.50	2.88
Chaturvedi [19]	1995	2	1.81	0.92	3.36	0.38	2.55
Liou [59]	1997	2	2.89	2.28	3.66	2.85	4.36
Chan [79]	1998	2	0.75	0.26	2.22	0.14	1.39
McCann [125]	1998	2	1.2	0.8	1.5	1.61	4.03
Smargiassi [134]	1998	2	1.15	0.56	2.36	0.31	2.29
Fall [80]	1999	2	2.8	0.89	8.7	0.12	1.27
Kuopio [81]	1999	2	1.02	0.63	1.65	0.69	3.24
Taylor [102]	1999	2	1.02	0.90	1.17	9.27	4.72
Werneck [126]	1999	2	2.49	0.53	13.1	0.06	0.73
Preux [127]	2000	2	1.34	0.85	2.10	0.78	3.38
Engel [135]	2001	2	0.8	0.5	1.2	0.83	3.45
Zorzon [128]	2002	2	1.6	1.0	2.4	0.83	3.45
Baldereschi [107]	2003	2	3.68	1.57	8.64	0.22	1.88
Baldi [149]	2003	2	2.20	1.11	4.34	0.34	2.42
Dong [84]	2003	2	1.19	0.54	2.61	0.26	2.07
Duzcan [150]	2003	2	2.96	1.31	6.69	0.24	1.99
Park RM [151]	2005	2	1.14	1.09	1.20	69.02	4.88
Wright [129]	2005	2	1.2	0.3	4.8	0.08	0.94
Dick [136]	2007	2	1.25	0.97	1.61	2.48	4.30
Fong [89]	2007	2	1.68	1.03	2.76	0.66	3.19
Kamel [18]	2007	2	1.3	0.5	3.3	0.18	1.65
Dhillon [92]	2008	2	4.4	0.5	38.1	0.03	0.43
Hancock [137]	2008	2	1.61	1.13	2.29	1.28	3.85
Petersen [91]	2008	2	6.00	0.62	57.6	0.03	0.40
Elbaz [55]	2009	2	1.7	1.0	2.9	0.56	3.02

Table F: RRs, 95% CIs and fixed or random effects study weights: Pesticide use (continued)

Author	Year	Tier	RR	LCL	UCL	Fixed Effects Normalized Study Weight	Random Effects Normalized Study Weight
Tanner [154]	2009	2	1.90	1.12	3.21	0.58	3.04
Kiyohara [94]	2010	2	0.79	0.57	1.10	1.48	3.96
Sanyal [130]	2010	2	17.12	4.97	58.8	0.10	1.12
Das [58]	2011	2	6.18	3.71	10.2	0.61	3.12
Rugbjerg [156]	2011	2	1.18	0.65	2.14	0.45	2.75
						100%	100%
**Meta-Analysis (Fixed)**			1.21*	1.17	1.26		
**Meta-Analysis (Random)**			1.61*	1.39	1.87		

*Statistically significant (P < 0.05; 95% CI excludes 1.0).

Table G: RRs, 95% CIs and fixed or random effects study weights: Herbicide use

Author	Year	Tier	RR	LCL	UCL	Fixed Effects Normalized Study Weight	Random Effects Normalized Study Weight
Firestone [114]	2005	1	1.41	0.51	3.88	6.21	6.21
Frigerio [157]	2006	1	1.2	0.4	3.9	4.93	4.93
Brighina [158]	2008	1	1.25	0.94	1.66	79.10	79.10
Vlajinac [115]	2010	1	1.8	0.8	4.04	9.75	9.75
						100%	100%
**Meta-Analysis (Fixed)**			1.30*	1.01	1.68		
**Meta-Analysis (Random)**			1.30*	1.01	1.68		
Stern [119]	1991	2	0.9	0.6	1.5	9.90	9.02
Semchuk [143]	1992	2	2.91	1.06	8.01	2.03	3.66
Hertzman [132]	1994	2	1.19	0.57	2.45	3.91	5.71
Seidler [121]	1996	2	1.65	1.17	2.33	17.51	10.80
Gorell [122]	1998	2	4.1	1.37	12.24	1.73	3.24
Taylor [102]	1999	2	1.06	0.68	1.65	10.57	9.25
Kuopio [81]	1999	2	1.4	0.79	2.48	6.35	7.44
Behari [104]	2001	2	0.5	0.28	0.88	6.34	7.44
Engel [135]	2001	2	0.9	0.6	1.3	13.90	10.13
Dhillon [92]	2008	2	0.8	0.4	1.4	5.29	6.79
Hancock [137]	2008	2	1.59	1	2.54	9.56	8.90
Elbaz [55]	2009	2	1.35	0.76	2.37	6.42	7.49
Tanaka [155]	2011	2	0.87	0.39	1.88	3.36	5.19
Rugbjerg [156]	2011	2	1.16	0.51	2.6	3.13	4.96
						100%	100%
**Meta-Analysis (Fixed)**			1.16*	1.01	1.34		
**Meta-Analysis (Random)**			1.17	0.94	1.46		

*Statistically significant ($P < 0.05$; 95% CI excludes 1.0).

Table H: RRs, 95% CIs and fixed or random effects study weights: Fungicide use

Author	Year	Tier	RR	LCL	UCL	Fixed Effects Normalized Study Weight	Random Effects Normalized Study Weight
Firestone [114]	2005	1	0.38	0.07	2.05	11.12	11.12
Brighina [158]	2008	1	0.83	0.44	1.59	76.81	76.81
Vlajinac [115]	2010	1	2.03	0.4	10.22	12.07	12.07
						100%	100%
**Meta-Analysis (Fixed)**			0.85	0.48	1.49		
**Meta-Analysis (Random)**			0.85	0.48	1.49		
Semchuk [143]	1992	2	1.63	0.81	3.29	13.39	15.63
Hertzman [132]	1994	2	0.52	0.25	1.08	12.29	14.68
Gorell [122]	1998	2	1.6	0.47	5.45	4.38	6.30
Engel [135]	2001	2	0.8	0.6	1.3	44.01	31.27
Elbaz [55]	2009	2	1.5	0.8	3	15.06	16.98
Tanaka [155]	2011	2	0.94	0.34	2.47	6.69	9.08
Rugbjerg [156]	2011	2	0.95	0.27	3.31	4.19	6.06
						100%	100%
**Meta-Analysis (Fixed)**			0.96	0.75	1.24		
**Meta-Analysis (Random)**			1.00	0.72	1.39		

Table I: RRs, 95% CIs and fixed or random effects study weights: Insecticide use

Author	Year	Tier	RR	LCL	UCL	Fixed Effects Normalized Study Weight	Random Effects Normalized Study Weight
Firestone [114]	2005	1	0.88	0.44	1.76	10.48	26.67
Frigerio [157]	2006	1	2.5	0.6	9.8	2.58	12.48
Brighina [158]	2008	1	0.95	0.74	1.22	80.61	39.41
Vlajinac [115]	2010	1	3.22	1.32	7.87	6.32	21.43
						100%	100%
**Meta-Analysis (Fixed)**			1.04	0.83	1.31		
**Meta-Analysis (Random)**			1.36	0.76	2.46		
Stern [119]	1991	2	0.5	0.2	1.1	4.63	7.81
Semchuk [143]	1992	2	2.05	1.03	4.07	7.12	8.56
Hertzman [132]	1994	2	0.33	0.12	0.9	3.31	7.10
Seidler [121]	1996	2	1.6	0.07	3.4	0.89	3.81
Gorell [122]	1998	2	3.55	1.75	7.18	6.75	8.48
Fall [80]	1999	2	2.2	0.48	9	1.56	5.24
Behari [104]	2001	2	0.73	0.45	1.17	14.72	9.43
Engel [135]	2001	2	0.9	0.6	1.5	16.01	9.50
Hancock [137]	2008	2	1.83	1.2	2.81	18.57	9.62
Dhillon [92]	2008	2	2.2	0.4	11.4	1.20	4.55
Elbaz [55]	2009	2	2.2	1.1	4.3	7.23	8.59
Rugbjerg [156]	2011	2	0.86	0.38	1.93	5.09	7.99
Das [58]	2011	2	6.18	3.71	10.29	12.92	9.31
						100%	100%
**Meta-Analysis (Fixed)**			1.53*	1.28	1.84		
**Meta-Analysis (Random)**			1.46	0.90	2.34		

*Statistically significant (P < 0.05; 95% CI excludes 1.0).

Table J: RRs, 95% CIs and fixed or random effects study weights: High herbicide use

Author	Year	Tier	RR	LCL	UCL	Fixed Effects Normalized Study Weight	Random Effects Normalized Study Weight
Vlajinac [115]	2010	1	2.8	0.62	12.77	100.00	100.00
			---	---	---	100%	100%
**Meta-Analysis (Fixed)**			---	---	---		
**Meta-Analysis (Random)**			---	---	---		
Semchuk [143]	1992	2	4.88	1.28	18.6	6.04	8.75
Butterfield [98]	1993	2	3.66	1.33	10.07	10.55	11.83
Seidler [121]	1996	2	2.2	0.9	5.2	14.05	13.40
Kuopio [81]	1999	2	0.79	0.38	1.66	19.89	15.18
Engel [135]	2001	2	0.8	0.4	1.7	20.65	15.36
Duzcan [150]	2003	2	0.19	0.01	3.33	1.28	2.69
Elbaz [55][1]	2009	2	0.9	0.3	2.2	10.89	12.00
Elbaz [55][1]	2009	2	2.2	0.8	6.4	10.00	11.53
Sanyal [130]	2010	2	3.07	0.86	11.03	6.64	9.26
						100%	100%
**Meta-Analysis (Fixed)**			1.45*	1.04	2.01		
**Meta-Analysis (Random)**			1.59	0.96	2.65		

[1]RRs based on PD risk in men aged < 65 years or ≥ 65 years.
*Statistically significant (P < 0.05; 95% CI excludes 1.0).

Table K: RRs, 95% CIs and fixed or random effects study weights: High fungicide use

Author	Year	Tier	RR	LCL	UCL	Fixed Effects Normalized Study Weight	Random Effects Normalized Study Weight
Vlajinac [115]	2010	1	2.03	0.28	14.6	100.00	100.00
			---	---	---	100%	100%
**Meta-Analysis (Fixed)**			---	---	---		
**Meta-Analysis (Random)**			---	---	---		
Engel [135]	2001	2	0.8	0.4	1.7	41.38	30.48
Duzcan [150]	2003	2	2.49	1.08	5.74	31.05	28.29
Elbaz [55][1]	2009	2	4.8	1.2	19.3	11.23	18.76
Elbaz [55][1]	2009	2	0.7	0.2	2	16.34	22.47
						100%	100%
**Meta-Analysis (Fixed)**			1.36	0.86	2.17		
**Meta-Analysis (Random)**			1.50	0.65	3.43		

[1]RRs based on PD risk in men aged < 65 years or ≥ 65 years.

Table L: RRs, 95% CIs and fixed or random effects study weights: High insecticide use

Author	Year	Tier	RR	LCL	UCL	Fixed Effects Normalized Study Weight	Random Effects Normalized Study Weight
Vlajinac [115]	2010	1	4.53	1.48	13.93	100.00	100.00
			---	---	---	100%	100%
**Meta-Analysis (Fixed)**			---	---	---		
**Meta-Analysis (Random)**			---	---	---		
Semchuk [143]	1992	2	3.5	1.03	11.96	8.41	12.68
Butterfield [98]	1993	2	4.3	1.35	13.67	9.43	13.47
Seidler [121]	1996	2	1.6	0.07	3.4	3.35	7.01
Engel [135]	2001	2	1.2	0.7	2.1	41.89	22.21
Duzcan [150]	2003	2	4.43	1.88	10.45	17.18	17.47
Elbaz [55][1]	2009	2	5.4	1.5	20	7.54	11.94
Elbaz [55][1]	2009	2	0.9	0.3	2.3	12.19	15.21
						100%	100%
**Meta-Analysis (Fixed)**			2.02*	1.42	2.89		
**Meta-Analysis (Random)**			2.40*	1.32	4.35		

[1]RRs based on PD risk in men aged < 65 years or ≥ 65 years.
*Statistically significant (P < 0.05; 95% CI excludes 1.0).

Table M: RRs, 95% CIs and fixed or random effects study weights: Paraquat use

Author	Year	Tier	RR	LCL	UCL	Fixed Effects Normalized Study Weight	Random Effects Normalized Study Weight
Firestone [57]	2010	1	0.9	0.14	5.43	100.00	100.00
			---	---	----	100%	100%
**Meta-Analysis (Fixed)**			---	---	---		
**Meta-Analysis (Random)**			---	---	---		
Hertzman [132]	1994	2	1.25	0.34	4.63	1.50	5.54
Liou [59]	1997	2	3.22	2.41	4.31	30.26	14.40
Kuopio [81]	1999	2	1.21	0.28	5.13	1.21	4.79
Engel [135]	2001	2	0.8	0.5	1.3	11.20	12.61
Kamel [18]	2007	2	1	0.5	1.9	8.47	11.85
Dhillon [92]	2008	2	3.5	0.4	31.6	5.74	10.61
Elbaz [55]	2009	2	1.2	0.7	2.1	0.54	2.56
Tanner [154]	2009	2	2.8	0.81	9.72	1.66	5.90
Rugbjerg [156]	2011	2	1.01	0.2	5.01	0.99	4.14
Tanner [160]	2011	2	2.5	1.4	4.7	6.97	11.26
Tomenson [165]	2011	2	0.32	0.01	1.76	0.38	1.92
Lee [161]	2012	2	1.36	1.02	1.81	31.09	14.43
						100%	100%
**Meta-Analysis (Fixed)**			1.70*	1.45	1.99		
**Meta-Analysis (Random)**			1.50*	1.02	2.19		

*Statistically significant (P < 0.05; 95% CI excludes 1.0).

Table N: RRs, 95% CIs and fixed or random effects study weights: High paraquat use

Author	Year	Tier	RR	LCL	UCL	Fixed Effects Normalized Study Weight	Random Effects Normalized Study Weight
Liou [59]	1997	2	6.44	2.41	17.2	15.27	22.91
Engel [135]	2001	2	0.7	0.3	1.9	17.31	23.74
Gatto [69]	2009	2	1.26	0.72	2.2	47.28	28.67
Goldman [163]	2012	2	3.1	1.3	7.2	20.13	24.68
						100%	100%
**Meta-Analysis (Fixed)**			1.75*	1.19	2.57		
**Meta-Analysis (Random)**			1.99	0.84	4.72		

*Statistically significant (P < 0.05; 95% CI excludes 1.0).
