# Supplementary material for: Association between Parkinson’s Disease and Cigarette Smoking, Rural Living, Well-Water Consumption, Farming and Pesticide Use: Systematic Review and Meta-Analysis
Source: PLoS One. 2016 Apr 7;11(4):e0151841. doi: 10.1371/journal.pone.0151841 (PMC4824443; doi:10.1371/journal.pone.0151841)
Supplement: S5 File — Tables A to J: Normalized study weights for Tier 1 and Tier 2 studies combined (i.e., all studies) based upon fixed and random effects models and RRs and 95% CLs for all individual studies. Table A: RRs, 95% CIs and fixed or random effects study weights for all studies: Current cigarette smoking. Table B: RRs, 95% CIs and fixed or random effects study weights for all studies: Heavy or long-term cigarette smoking. Table C: RRs, 95% CIs and fixed or random effects study weights for all studies: Rural living. Table D: RRs, 95% CIs and fixed or random effects study weights for all studies: Well-water consumption. Table E: RRs, 95% CIs and fixed or random effects study weights for all studies: Farming. Table F: RRs, 95% CIs and fixed or random effects study weights for all studies: Pesticide use. Table G: RRs, 95% CIs and fixed or random effects study weights for all studies: Herbicide use. Table H: RRs, 95% CIs and fixed or random effects study weights for all studies: Fungicide use. Table I: RRs, 95% CIs and fixed or random effects study weights for all studies: Insecticide use. Table J: RRs, 95% CIs and fixed or random effects study weights for all studies: Paraquat use. (PDF) [file pone.0151841.s007.pdf]

Table A: RRs, 95% CIs and fixed or random effects study weights for all studies: Current cigarette smoking

Author	Year	Tier	RR	LCI	UCL	Fixed Effects Normalized Study Weight	Random Effects Normalized Study Weight
Benedetti [60]	2000	1	1.14	0.41	3.15	0.88	1.99
Hernán [61]	2001	1	0.4	0.2	0.6	3.03	3.64
Checkoway [62]	2002	1	0.3	0.1	0.7	0.96	2.11
Wirdefeldt [64]	2005	1	0.56	0.4	0.79	7.89	4.61
Park [63]	2005	1	0.2	0.12	0.34	3.37	3.77
Thacker [65]	2007	1	0.27	0.13	0.56	1.71	2.89
Tan [66]	2008	1	0.29	0.16	0.52	2.63	3.46
Sääksjärvi [67]	2008	1	0.19	0.07	0.52	0.91	2.03
Costello [68]	2009	1	0.48	0.27	0.86	2.72	3.51
Shino [72]	2010	1	0.13	0.07	0.28	1.90	3.03
Liu [74]	2012	1	0.56	0.42	0.75	10.86	4.82
Mayeux [75]	1994	2	0.2	0.1	0.5	1.41	2.62
Martyn [76]	1995	2	0.5	0.28	0.93	2.53	3.41
Tzourio [78]	1997	2	0.7	0.4	1.3	2.63	3.46
Hellenbrand [77]	1997	2	0.2	0.1	0.3	3.03	3.64
Chan [79]	1998	2	0.51	0.26	1.01	1.98	3.09
Kuopio [81]	1999	2	0.5	0.2	1.24	1.10	2.28
Fall [80]	1999	2	0.17	0.06	0.43	0.94	2.08
Paganini-Hill [82]	2001	2	0.42	0.25	0.69	3.54	3.83
Tsai [83]	2002	2	0.3	0.03	3.11	0.17	0.54
Ragonese [85]	2003	2	0.87	0.46	1.65	2.24	3.25
Dong [84]	2003	2	0.44	0.23	0.86	2.10	3.17
Ascherio [3]	2004	2	0.66	0.54	0.81	22.21	5.16
Galanaud [87]	2005	2	0.5	0.2	1	1.41	2.62
Fong [89]	2007	2	0.86	0.45	1.64	2.18	3.22
Kamel [18]	2007	2	0.6	0.2	1.7	0.80	1.87
Hancock [88]	2007	2	0.3	0.17	0.53	2.82	3.55
Dhillon [92]	2008	2	1.1	0.3	5.2	0.45	1.23
Petersen [91]	2008	2	0.63	0.26	1.55	1.15	2.34
Powers [90]	2008	2	0.45	0.29	0.7	4.70	4.14
Nicoletti [95]	2010	2	0.37	0.22	0.64	3.20	3.71
Tanaka [93]	2010	2	0.12	0.05	0.27	1.28	2.49
Kyrozis [20]	2013	2	0.42	0.18	0.99	1.26	2.46
						100%	100%
**Meta-Analysis (Fixed)**			0.46*	0.42	0.51		
**Meta-Analysis (Random)**			0.41*	0.34	0.48		

*Statistically significant ($P < 0.05$; 95% CI excludes 1.0).

Table B: RRs, 95% CIs and fixed or random effects study weights for all studies: Heavy or long-term cigarette smoking

Author	Year	Tier	RR	LCL	UCL	Fixed Effects Normalized Study Weight	Random Effects Normalized Study Weight
Grandinetti [96]	1994	1	0.77	0.66	0.9	19.82	5.12
Benedetti [60]	2000	1	0.69	0.32	1.48	0.81	2.06
Hernán [61]	2001	1	0.3	0.2	0.6	1.58	2.95
Checkoway [62]	2002	1	0.4	0.2	0.8	0.99	2.32
Wirdefeldt [64]	2005	1	0.52	0.29	0.95	1.35	2.74
Thacker [65]	2007	1	0.55	0.35	0.86	2.36	3.48
Tan [66]	2008	1	0.18	0.07	0.45	0.55	1.59
Costello [68]	2009	1	0.54	0.38	0.78	3.69	4.00
Chen [71]	2010	1	0.57	0.44	0.74	7.06	4.59
Liu [74]	2012	1	0.68	0.55	0.83	11.26	4.88
Sasco [97]	1990	2	0.27	0.08	0.96	0.31	1.02
Wang [99]	1993	2	0.5	0.23	1.09	0.79	2.02
Butterfield [98]	1993	2	0.32	0.15	0.67	0.85	2.12
Mayeux [75]	1994	2	0.6	0.3	1.2	0.99	2.32
Morano [100]	1994	2	0.23	0.04	1.2	0.16	0.60
Liou [59]	1997	2	0.43	0.2	0.9	0.84	2.11
Hellenbrand [77]	1997	2	0.3	0.2	0.6	1.58	2.95
Taylor [102]	1999	2	0.82	0.66	0.9	19.82	5.12
Fall [80]	1999	2	0.31	0.11	0.78	0.50	1.47
Vanacore [103]	2000	2	0.32	0.15	0.66	0.87	2.14
Behari [104]	2001	2	0.74	0.46	1.19	2.11	3.34
Paganini-Hill [82]	2001	2	0.42	0.22	0.8	1.14	2.51
Ragonese [85]	2003	2	0.72	0.29	1.01	1.22	2.60
Baldereschi [107]	2003	2	0.6	0.32	1.12	1.21	2.59
Pals [106]	2003	2	0.44	0.23	0.82	1.18	2.55
Tan [105]	2003	2	0.38	0.2	0.72	1.16	2.53
Dong [84]	2003	2	0.35	0.18	0.7	1.03	2.37
Gorell [108]	2004	2	0.42	0.25	0.71	1.75	3.09
Ma [110]	2006	2	3.41	1.2	7.74	0.55	1.58
Evans [109]	2006	2	0.33	0.12	0.92	0.46	1.39
Kamel [18]	2007	2	1	0.4	2.3	0.62	1.73
Hancock [88]	2007	2	0.35	0.2	0.62	1.49	2.87
Petersen [91]	2008	2	0.53	0.26	1.08	0.94	2.25
Powers [90]	2008	2	0.44	0.31	0.64	3.63	3.99
Elbaz [55]	2009	2	0.4	0.2	0.7	1.21	2.59
Nicoletti [95]	2010	2	0.39	0.26	0.6	2.73	3.66
Tanaka [93]	2010	2	0.28	0.15	0.49	1.36	2.75
						100%	100%
**Meta-Analysis (Fixed)**			0.60*	0.56	0.65		
**Meta-Analysis (Random)**			0.49*	0.43	0.57		

*Statistically significant ($P < 0.05$; 95% CI excludes 1.0).

Table C: RRs, 95% CIs and fixed or random effects study weights for all studies: Rural living

Author	Year	Tier	RR	LCL	UCL	Fixed Effects Normalized Study Weight	Random Effects Normalized Study Weight
Baldi [56]	2003	1	1.37	0.56	3.33	0.42	2.10
Wirdefeldt [64]	2005	1	0.92	0.66	1.28	3.03	4.67
Firestone [114]	2005	1	1.31	0.84	2.03	1.71	4.06
Vlajinac [115]	2010	1	3.56	1.96	6.46	0.93	3.25
Tanner [117]	1989	2	0.57	0.33	0.98	1.12	3.51
Ho [116]	1989	2	4.9	1.4	18.2	0.20	1.25
Koller [118]	1990	2	1.88	1.13	3.19	1.23	3.64
Stern [119]	1991	2	1.7	0.9	3.1	0.87	3.14
Jiménez-J. [120]	1992	2	1.07	0.69	1.63	1.80	4.12
Wang [99]	1993	2	0.76	0.49	1.18	1.72	4.07
Hubble [54]	1993	2	2.25	0.6	8.42	0.19	1.19
Butterfield [98]	1993	2	2.35	0.87	6.34	0.34	1.82
Hubble [54]	1993	2	6.49	2.35	17.9	0.32	1.77
Morano [100]	1994	2	1.47	0.79	2.71	0.87	3.15
Martyn [76]	1995	2	1.4	0.82	2.49	1.08	3.45
Seidler [121]	1996	2	0.83	0.34	2	0.42	2.12
Liou [59]	1997	2	2.04	1.23	3.38	1.30	3.71
Marder [124]	1998	2	0.8	0.32	1.98	0.40	2.05
Gorell [122]	1998	2	1.19	0.73	1.93	1.41	3.81
McCann [125]	1998	2	1.7	1.17	2.57	2.15	4.32
De Palma [123]	1998	2	3.62	2.09	6.26	1.10	3.48
Werneck [126]	1999	2	1	0.52	1.95	0.76	2.95
Taylor [102]	1999	2	1.07	0.99	1.15	59.22	5.73
Preux [127]	2000	2	1.67	1	2.5	1.58	3.96
Behari [104]	2001	2	0.94	0.7	1.25	3.95	4.90
Zorzon [128]	2002	2	1.5	1	2.4	1.73	4.08
Wright [129]	2005	2	1.1	0.8	1.3	5.64	5.14
Sanyal [130]	2010	2	4.05	2.53	6.49	1.50	3.89
Das [58]	2011	2	1.05	0.75	1.46	3.00	4.66
						100%	100%
**Meta-Analysis (Fixed)**			1.17*	1.10	1.24		
**Meta-Analysis (Random)**			1.43*	1.22	1.69		

*Statistically significant (P < 0.05; 95% CI excludes 1.0).

Table D: RRs, 95% CIs and fixed or random effects study weights for all studies: Well-water consumption

Author	Year	Tier	RR	LCL	UCL	Fixed Effects Normalized Study Weight	Random Effects Normalized Study Weight
Park [131]	2004	1	0.62	0.29	1.32	0.35	2.10
Park [63]	2005	1	1.71	1.14	2.45	1.36	3.57
Firestone [114]	2005	1	1.81	1.02	3.21	0.61	2.75
Gatto [69]	2009	1	1.21	0.82	1.8	1.29	3.52
Vlajinac [115]	2010	1	2.62	1.4	4.9	0.51	2.54
Tanner [117]	1989	2	0.74	0.41	1.32	0.58	2.70
Koller [118]	1990	2	1.67	1.01	2.79	0.77	3.01
Stern [119]	1991	2	0.8	0.4	1.6	0.41	2.31
Jiménez-J. [120]	1992	2	1.22	0.77	1.94	0.93	3.21
Wang [99]	1993	2	0.59	0.36	0.95	0.85	3.11
Hertzman [132]	1994	2	0.9	0.52	1.55	0.67	2.86
Morano [100]	1994	2	3.28	0.93	11.51	0.13	1.06
Seidler [121]	1996	2	0.8	0.6	1.2	1.66	3.73
De Michele [133]	1996	2	2.17	1.28	3.69	0.71	2.92
Liou [59]	1997	2	1.07	0.19	5.98	0.07	0.63
McCann [125]	1998	2	0.6	0.38	0.92	1.02	3.30
Gorell [122]	1998	2	0.97	0.65	1.4	1.35	3.56
Chan [79]	1998	2	1.04	0.7	1.54	1.28	3.51
Marder [124]	1998	2	1.79	1.04	3.1	0.67	2.86
Smargiassi [134]	1998	2	2.78	1.46	5.28	0.48	2.48
Taylor [102]	1999	2	0.93	0.88	0.98	68.78	4.66
Kuopio [81]	1999	2	0.97	0.59	1.6	0.80	3.05
Werneck [126]	1999	2	1.49	0.74	3.01	0.40	2.28
Preux [127]	2000	2	1.19	0.77	1.84	1.05	3.33
Engel [135]	2001	2	0.9	0.6	1.5	0.95	3.23
Behari [104]	2001	2	1.94	1.33	2.8	1.44	3.61
Zorzon [128]	2002	2	2	1.1	3.6	0.57	2.67
Tsai [83]	2002	2	10.9	1.77	67.5	0.06	0.58
Dong [84]	2003	2	0.96	0.48	1.92	0.41	2.31
Wright [129]	2005	2	8.3	2.5	27.6	0.14	1.14
Dick [136]	2007	2	1.23	1	1.52	4.55	4.28
Hancock [137]	2008	2	1.08	0.77	1.5	1.79	3.78
Elbaz [55]	2009	2	1	0.7	1.5	1.37	3.57
Sanyal [130]	2010	2	4.5	2.1	9.9	0.33	2.04
Das [58]	2011	2	2.5	1.77	3.54	1.66	3.73
						100%	100%
**Meta-Analysis (Fixed)**			1.02	0.98	1.07		
**Meta-Analysis (Random)**			1.30*	1.12	1.51		

*Statistically significant (P < 0.05; 95% CI excludes 1.0).

Table E: RRs, 95% CIs and fixed or random effects study weights for all studies: Farming

Author	Year	Tier	RR	LCL	UCL	Fixed Effects Normalized Study Weight	Random Effects Normalized Study Weight
Park [131]	2004	1	0.27	0.06	1.1	0.05	0.45
Frigerio [138]	2005	1	1.5	0.8	2.5	0.35	1.94
Park [63]	2005	1	1.64	0.96	2.81	0.39	2.08
Ascherio [139]	2006	1	1.6	0.9	2.7	0.37	2.03
Firestone [57]	2010	1	1.06	0.85	3.01	0.28	1.70
Vlajinac [115]	2010	1	1.31	0.68	2.52	0.40	2.09
Skeie [140]	2010	1	1.75	1.03	3	0.26	1.63
Feldman [73]	2011	1	0.9	0.6	1.4	0.63	2.66
Ho [116]	1989	2	5.2	1.6	17.7	0.08	0.63
Koller [118]	1990	2	1.33	0.85	2.11	0.16	1.12
Hertzman [141]	1990	2	2.98	1.28	6.97	0.55	2.48
Tanner [142]	1990	2	3	1	9.8	0.09	0.69
Semchuk [143]	1992	2	1.94	1.12	3.34	0.38	2.04
Hertzman [132]	1994	2	0.72	0.22	2.3	0.08	0.66
Chaturvedi [19]	1995	2	1.42	0.86	2.37	0.44	2.22
Rocca [144]	1996	2	0.6	0.3	1.3	0.21	1.39
Seidler [121]	1996	2	0.9	0.6	1.4	0.63	2.66
Liou [59]	1997	2	1.81	1.25	2.64	0.81	2.96
Chan [79]	1998	2	0.92	0.59	1.43	0.58	2.55
Smargiassi [134]	1998	2	1.25	0.65	2.43	0.73	2.84
Gorell [122]	1998	2	1.3	0.88	1.93	0.04	0.32
Marder [124]	1998	2	13.6	2.4	79.5	0.26	1.61
Tsui [145]	1999	2	0.68	0.32	1.41	0.21	1.41
Fall [80]	1999	2	1.4	0.68	2.9	0.45	2.23
Kuopio [81]	1999	2	1.45	0.88	2.41	0.21	1.37
Preux [127]	2000	2	1.06	0.71	1.59	0.70	2.78
Tuchsen [146]	2000	2	1.32	1.11	1.56	3.90	4.30
Behari [104]	2001	2	0.72	0.48	1.05	0.74	2.85
Engel [135]	2001	2	1	0.5	1.8	0.28	1.67
Lee [148]	2002	2	0.86	0.81	0.92	27.88	4.80
Zorzon [128]	2002	2	7.7	1.4	44.1	0.04	0.33
Baldi [149]	2003	2	0.88	0.44	1.56	0.45	2.24
Dong [84]	2003	2	0.75	0.42	1.32	0.28	1.70
Baldereschi [107]	2003	2	1.2	0.72	1.97	0.34	1.93
Duzcan [150]	2003	2	1.69	0.78	3.65	0.19	1.29
Wright [129]	2005	2	1	0.4	2.8	0.94	3.13
Park [151]	2005	2	1.14	1.08	1.19	48.06	4.83
Goldman [152]	2005	2	3	2.1	4.2	0.12	0.90

Table E: RRs, 95% CIs and fixed or random effects study weights for all studies: Farming (continued)

Author	Year	Tier	RR	LCL	UCL	Fixed Effects Normalized Study Weight	Random Effects Normalized Study Weight
Dick [153]	2007	2	1.02	0.82	1.28	2.28	3.97
Dhillon [92]	2008	2	1.1	0.6	2	0.31	1.81
Hancock [137]	2008	2	1.11	0.8	1.54	1.05	3.25
Tanner [154]	2009	2	1.1	0.78	1.57	0.70	2.79
Elbaz [55]	2009	2	1.9	1.3	2.9	0.92	3.11
Sanyal [130]	2010	2	2.01	0.14	27.93	0.02	0.14
Tanaka [155]	2011	2	0.95	0.41	2.15	1.28	3.46
Das [58]	2011	2	1.08	0.8	1.45	0.21	1.38
Rugbjerg [156]	2011	2	2.47	1.18	5.15	0.16	1.16
Kyrozis [20]	2013	2	0.97	0.61	1.55	0.52	2.42
						100%	100%
**Meta-Analysis (Fixed)**			1.08*	1.04	1.11		
**Meta-Analysis (Random)**			1.24*	1.12	1.37		

*Statistically significant (P < 0.05; 95% CI excludes 1.0).

Table F: RRs, 95% CIs and fixed or random effects study weights for all studies: Pesticide use

Author	Year	Tier	RR	LCI	UCL	Fixed Effects Normalized Study Weight	Random Effects Normalized Study Weight
Baldi [56]	2003	1	5.63	1.47	21.58	0.08	0.74
Baldi [56]	2003	1	1.02	0.22	4.82	0.06	0.58
Ascherio [139]	2006	1	1.8	1.3	2.5	1.37	3.15
Frigerio [157]	2006	1	1.5	0.8	2.9	0.35	1.97
Brighina [158]	2008	1	1.11	0.89	1.38	3.05	3.55
Costello [68]	2009	1	1.52	1.08	2.14	1.26	3.09
Firestone [57]	2010	1	0.6	0.30	1.29	0.28	1.73
Firestone [57]	2010	1	3.9	0.39	39.4	0.03	0.28
Skeie [140]	2010	1	1.06	0.62	1.82	0.51	2.33
Vlajinac [115]	2010	1	3.44	1.81	6.53	0.36	1.98
Feldman [73]	2011	1	0.9	0.5	1.3	0.64	2.55
Ho [116]	1989	2	3.6	1.0	12.9	0.09	0.80
Hertzman [141]	1990	2	1.34	0.71	2.52	0.37	2.01
Koller [118]	1990	2	1.05	0.67	1.65	0.72	2.65
Jiménez-J. [120]	1992	2	1.34	0.85	2.13	0.70	2.62
Semchuk [143]	1992	2	2.25	1.27	3.99	0.45	2.21
Hubble [54]	1993	2	3.42	1.27	7.32	0.19	1.38
Hertzman [132]	1994	2	2.32	1.10	4.88	0.26	1.69
Morano [100]	1994	2	1.73	0.98	3.03	0.46	2.23
Chaturvedi [19]	1995	2	1.81	0.92	3.36	0.35	1.96
Liou [59]	1997	2	2.89	2.28	3.66	2.62	3.49
Chan [79]	1998	2	0.75	0.26	2.22	0.13	1.04
McCann [125]	1998	2	1.2	0.8	1.5	1.49	3.20
Smargiassi [134]	1998	2	1.15	0.56	2.36	0.28	1.76
Fall [80]	1999	2	2.8	0.89	8.7	0.11	0.95
Kuopio [81]	1999	2	1.02	0.63	1.65	0.63	2.54
Taylor [102]	1999	2	1.02	0.90	1.17	8.53	3.81
Werneck [126]	1999	2	2.49	0.53	13.14	0.06	0.55
Preux [127]	2000	2	1.34	0.85	2.10	0.72	2.65
Engel [135]	2001	2	0.8	0.5	1.2	0.77	2.70
Zorzon [128]	2002	2	1.6	1.0	2.4	0.77	2.70
Baldereschi [107]	2003	2	3.68	1.57	8.64	0.20	1.43
Baldi [149]	2003	2	2.20	1.11	4.34	0.32	1.86
Dong [84]	2003	2	1.19	0.54	2.61	0.24	1.58
Duzcan [150]	2003	2	2.96	1.31	6.69	0.22	1.52
Park RM [151]	2005	2	1.14	1.09	1.20	63.51	3.94
Wright [129]	2005	2	1.2	0.3	4.8	0.08	0.70
Dick [136]	2007	2	1.25	0.97	1.61	2.29	3.43
Fong [89]	2007	2	1.68	1.03	2.76	0.60	2.49
Kamel [18]	2007	2	1.3	0.5	3.3	0.16	1.25

Table F: RRs, 95% CIs and fixed or random effects study weights for all studies: Pesticide use (continued)

Author	Year	Tier	RR	LCI	UCL	Fixed Effects Normalized Study Weight	Random Effects Normalized Study Weight
Dhillon [92]	2008	2	4.4	0.5	38.1	0.03	0.32
Hancock [137]	2008	2	1.61	1.13	2.29	1.18	3.04
Petersen [91]	2008	2	6.00	0.62	57.68	0.03	0.29
Elbaz [55]	2009	2	1.7	1.0	2.9	0.52	2.35
Tanner [154]	2009	2	1.90	1.12	3.21	0.53	2.37
Kiyohara [94]	2010	2	0.79	0.57	1.10	1.36	3.14
Sanyal [130]	2010	2	17.12	4.97	58.84	0.10	0.84
Das [58]	2011	2	6.18	3.71	10.29	0.56	2.43
Rugbjerg [156]	2011	2	1.18	0.65	2.14	0.41	2.13
						100%	100%
**Meta-Analysis (Fixed)**			1.22*	1.18	1.27		
**Meta-Analysis (Random)**			1.56*	1.37	1.77		

*Statistically significant (P < 0.05; 95% CI excludes 1.0).

Table G: RRs, 95% CIs and fixed or random effects study weights for all studies: Herbicide use

Author	Year	Tier	RR	LCL	UCL	Fixed Effects Normalized Study Weight	Random Effects Normalized Study Weight
Firestone [114]	2005	1	1.41	0.51	3.88	1.52	2.56
Frigerio [157]	2006	1	1.2	0.4	3.9	1.21	2.11
Brighina [158]	2008	1	1.25	0.94	1.66	19.39	10.87
Vlajinac [115]	2010	1	1.8	0.8	4.04	2.39	3.67
Stern [119]	1991	2	0.9	0.6	1.5	7.47	7.54
Semchuk [143]	1992	2	2.91	1.06	8.01	1.53	2.58
Hertzman [132]	1994	2	1.19	0.57	2.45	2.95	4.28
Seidler [121]	1996	2	1.65	1.17	2.33	13.22	9.63
Gorell [122]	1998	2	4.1	1.37	12.24	1.31	2.26
Taylor [102]	1999	2	1.06	0.68	1.65	7.98	7.79
Kuopio [81]	1999	2	1.4	0.79	2.48	4.79	5.90
Behari [104]	2001	2	0.5	0.28	0.88	4.78	5.90
Engel [135]	2001	2	0.9	0.6	1.3	10.49	8.81
Dhillon [92]	2008	2	0.8	0.4	1.4	4.00	5.27
Hancock [137]	2008	2	1.59	1	2.54	7.22	7.42
Elbaz [55]	2009	2	1.35	0.76	2.37	4.85	5.95
Tanaka [155]	2011	2	0.87	0.39	1.88	2.54	3.83
Rugbjerg [156]	2011	2	1.16	0.51	2.6	2.36	3.64
						100%	100%
**Meta-Analysis (Fixed)**			1.20*	1.06	1.36		
**Meta-Analysis (Random)**			1.20	1.00	1.43		

*Statistically significant ($P < 0.05$; 95% CI excludes 1.0).

Table H: RRs, 95% CIs and fixed or random effects study weights for all studies: Fungicide use

Author	Year	Tier	RR	LCL	UCL	Fixed Effects Normalized Study Weight	Random Effects Normalized Study Weight
Firestone [114]	2005	1	0.38	0.07	2.05	1.91	2.38
Brighina [158]	2008	1	0.83	0.44	1.59	13.20	13.95
Vlajinac [115]	2010	1	2.03	0.4	10.22	2.07	2.58
Semchuk [143]	1992	2	1.63	0.81	3.29	11.09	12.06
Hertzman [132]	1994	2	0.52	0.25	1.08	10.18	11.21
Gorell [122]	1998	2	1.6	0.47	5.45	3.63	4.40
Engel [135]	2001	2	0.8	0.6	1.3	36.44	29.35
Elbaz [55]	2009	2	1.5	0.8	3	12.47	13.31
Tanaka [155]	2011	2	0.94	0.34	2.47	5.54	6.53
Rugbjerg [156]	2011	2	0.95	0.27	3.31	3.47	4.22
						100%	100%
**Meta-Analysis (Fixed)**			0.94	0.75	1.19		
**Meta-Analysis (Random)**			0.96	0.74	1.25		

Table I: RRs, 95% CIs and fixed or random effects study weights for all studies: Insecticide use

Author	Year	Tier	RR	LCL	UCL	Fixed Effects Normalized Study Weight	Random Effects Normalized Study Weight
Firestone [114]	2005	1	0.88	0.44	1.76	4.20	6.53
Frigerio [157]	2006	1	2.5	0.6	9.8	1.03	3.84
Brighina [158]	2008	1	0.95	0.74	1.22	32.26	8.16
Vlajinac [115]	2010	1	3.22	1.32	7.87	2.53	5.67
Stern [119]	1991	2	0.5	0.2	1.1	2.77	5.84
Semchuk [143]	1992	2	2.05	1.03	4.07	4.27	6.56
Hertzman [132]	1994	2	0.33	0.12	0.9	1.99	5.20
Seidler [121]	1996	2	1.6	0.07	3.4	0.53	2.54
Gorell [122]	1998	2	3.55	1.75	7.18	4.05	6.48
Fall [80]	1999	2	2.2	0.48	9	0.94	3.64
Behari [104]	2001	2	0.73	0.45	1.17	8.83	7.43
Engel [135]	2001	2	0.9	0.6	1.5	9.60	7.50
Hancock [137]	2008	2	1.83	1.2	2.81	11.14	7.62
Dhillon [92]	2008	2	2.2	0.4	11.4	0.72	3.10
Elbaz [55]	2009	2	2.2	1.1	4.3	4.34	6.58
Rugbjerg [156]	2011	2	0.86	0.38	1.93	3.05	6.02
Das [58]	2011	2	6.18	3.71	10.29	7.75	7.30
						100%	100%
**Meta-Analysis (Fixed)**			1.32*	1.14	1.52		
**Meta-Analysis (Random)**			1.46*	1.01	2.11		

*Statistically significant (P < 0.05; 95% CI excludes 1.0).

Table J: RRs, 95% CIs and fixed or random effects study weights for all studies: Paraquat use

Author	Year	Tier	RR	LCL	UCL	Fixed Effects Normalized Study Weight	Random Effects Normalized Study Weight
Firestone [57]	2010	1	0.9	0.14	5.43	0.76	3.25
Hertzman [132]	1994	2	1.25	0.34	4.63	1.49	5.30
Liou [59]	1997	2	3.22	2.41	4.31	30.03	14.03
Kuopio [81]	1999	2	1.21	0.28	5.13	1.20	4.58
Engel [135]	2001	2	0.8	0.5	1.3	11.12	12.24
Kamel [18]	2007	2	1	0.5	1.9	8.41	11.49
Dhillon [92]	2008	2	3.5	0.4	31.6	5.69	10.27
Elbaz [55]	2009	2	1.2	0.7	2.1	0.53	2.43
Tanner [154]	2009	2	2.8	0.81	9.72	1.64	5.65
Rugbjerg [156]	2011	2	1.01	0.2	5.01	0.98	3.95
Tanner [160]	2011	2	2.5	1.4	4.7	6.92	10.90
Tomenson [165]	2011	2	0.32	0.01	1.76	0.38	1.82
Lee [161]	2012	2	1.36	1.02	1.81	30.85	14.06
						100%	100%
**Meta-Analysis (Fixed)**			1.69*	1.44	1.98		
**Meta-Analysis (Random)**			1.47*	1.01	2.13		

*Statistically significant (P < 0.05; 95% CI excludes 1.0).
